# Birth companionship in a government health system: a pilot study in Kigoma, Tanzania

**DOI:** 10.1186/s12884-021-03746-0

**Published:** 2021-04-16

**Authors:** Paul Chaote, Nguke Mwakatundu, Sunday Dominico, Alex Mputa, Agnes Mbanza, Magdalena Metta, Samantha Lobis, Michelle Dynes, Selemani Mbuyita, Shanon McNab, Karen Schmidt, Florina Serbanescu

**Affiliations:** 1 President’s Office - Regional Administration and Local Government, Dodoma, Tanzania; 2 Thamini Uhai, Dar es Salaam, Tanzania; 3 Vital Strategies, New York City, USA; 4 U.S. Centers for Disease Control and Prevention, Division of Reproductive Health, Atlanta, USA; 5 ICAP, Dar es Salaam, Tanzania; 6 Averting Maternal Death and Disability Program, Mailman School of Public Health, Columbia University, New York City, USA

**Keywords:** Birth companionship, Maternal and newborn health, Quality of care, Respectful care

## Abstract

**Background:**

Having a companion of choice throughout childbirth is an important component of good quality and respectful maternity care for women and has become standard in many countries. However, there are only a few examples of birth companionship being implemented in government health systems in low-income countries. To learn if birth companionship was feasible, acceptable and led to improved quality of care in these settings, we implemented a pilot project using 9 intervention and 6 comparison sites (all government health facilities) in a rural region of Tanzania.

**Methods:**

The pilot was developed and implemented in Kigoma, Tanzania between July 2016 and December 2018. Women delivering at intervention sites were given the choice of having a birth companion with them during childbirth. We evaluated the pilot with: (a) project data; (b) focus group discussions; (c) structured and semi-structured interviews; and (d) service statistics.

**Results:**

More than 80% of women delivering at intervention sites had a birth companion who provided support during childbirth, including comforting women and staying by their side. Most women interviewed at intervention sites were very satisfied with having a companion during childbirth (96–99%). Most women at the intervention sites also reported that the presence of a companion improved their labor, delivery and postpartum experience (82–97%). Health providers also found companions very helpful because they assisted with their workload, alerted the provider about changes in the woman’s status, and provided emotional support to the woman. When comparing intervention and comparison sites, providers at intervention sites were significantly more likely to: respond to women who called for help (*p* = 0.003), interact in a friendly way (*p* < 0.001), greet women respectfully (*p* < 0.001), and try to make them more comfortable (*p* = 0.003). Higher proportions of women who gave birth at intervention sites reported being “very satisfied” with the care they received (*p* < 0.001), and that the staff were “very kind” (*p* < 0.001) and “very encouraging” (*p* < 0.001).

**Conclusion:**

Birth companionship was feasible and well accepted by health providers, government officials and most importantly, women who delivered at intervention facilities. The introduction of birth companionship improved women’s experience of birth and the maternity ward environment overall.

**Supplementary Information:**

The online version contains supplementary material available at 10.1186/s12884-021-03746-0.

## Background

Despite progress in Tanzania, maternal and perinatal mortality rates remain high. The maternal mortality ratio in Tanzania is 556 maternal deaths per 100,000 live births and the neonatal mortality rate is 25 per 1000 live births [1]. The Government of Tanzania is committed to reducing maternal and neonatal mortality and has developed evidence-based strategies and targets in support of that goal [2–4]. The current strategy aims to reduce maternal and neonatal mortality by increasing institutional deliveries to 80% and increasing the coverage of good-quality emergency obstetric and newborn care (EmONC) services at all levels of the health system by 2020 [2–4].

Projects to improve the quality of maternal and newborn care, implemented by Thamini Uhai/Vital Strategies and partners in collaboration with the Government of Tanzania, have been in place in the Kigoma region since 2006. Kigoma, a mostly rural region in the Western Zone, has an estimated 92,000 births per year [5, 6] with maternal, reproductive, and neonatal health indicators that have lagged behind other regions in Tanzania [1, 7]. While partner efforts across the region likely contributed to increasing facility deliveries (48% in 2011 to 55% in 2015) [8], many women continued to deliver outside of health facilities [9].

Barriers found to influence place of delivery in low- and middle-income countries include traditional and family influences, distance to facility, cost, and a lack of supportive attendance and comforting care at facilities [10]. When women do seek facility delivery, many experience neglect and disrespectful care from the few health providers present [11–14]; in Tanzania, women report that provider attitudes, disrespect and abuse are important factors in their decision whether or not to seek facility delivery [15].

In Tanzania, with severe provider shortages [16], low job satisfaction among providers [17], and restrictions around bringing a companion of their choice into labor and delivery wards [18], women may not receive one-on-one supportive care during and after childbirth. The literature shows, however, that in general, women most value giving birth to a healthy baby in a safe environment with support from birth companions and skilled, kind staff [19].

The World Health Organization recognizes companionship of choice during childbirth as an important component of good quality and respectful maternity care [20–22]. Companionship, including continuous emotional and social support during childbirth, improves maternal and newborn health outcomes as well as women’s satisfaction with care [23]. Companions provide women with informational, practical and emotional support and can serve as advocates for women [23, 24] and their use has become standard in many high-income countries. However, there are few examples of birth companionship being integrated into health systems in low-income countries [24–29].

In recognition of current evidence and global recommendations, Thamini Uhai/Vital Strategies and the Government of Tanzania implemented a pilot project and collaborated with the U.S. Centers for Disease Control and Prevention, Division of Reproductive Health (CDC/DRH) and the Averting Maternal Death and Disability Program (AMDD) at Columbia University (through Ifakara Health Institute and ICAP Tanzania) to study the feasibility and acceptability of introducing birth companionship into the government health system, and its influence on facility use, quality of care, and women’s experience of care.

## Methods

### Pilot description

**Fig. 1:**
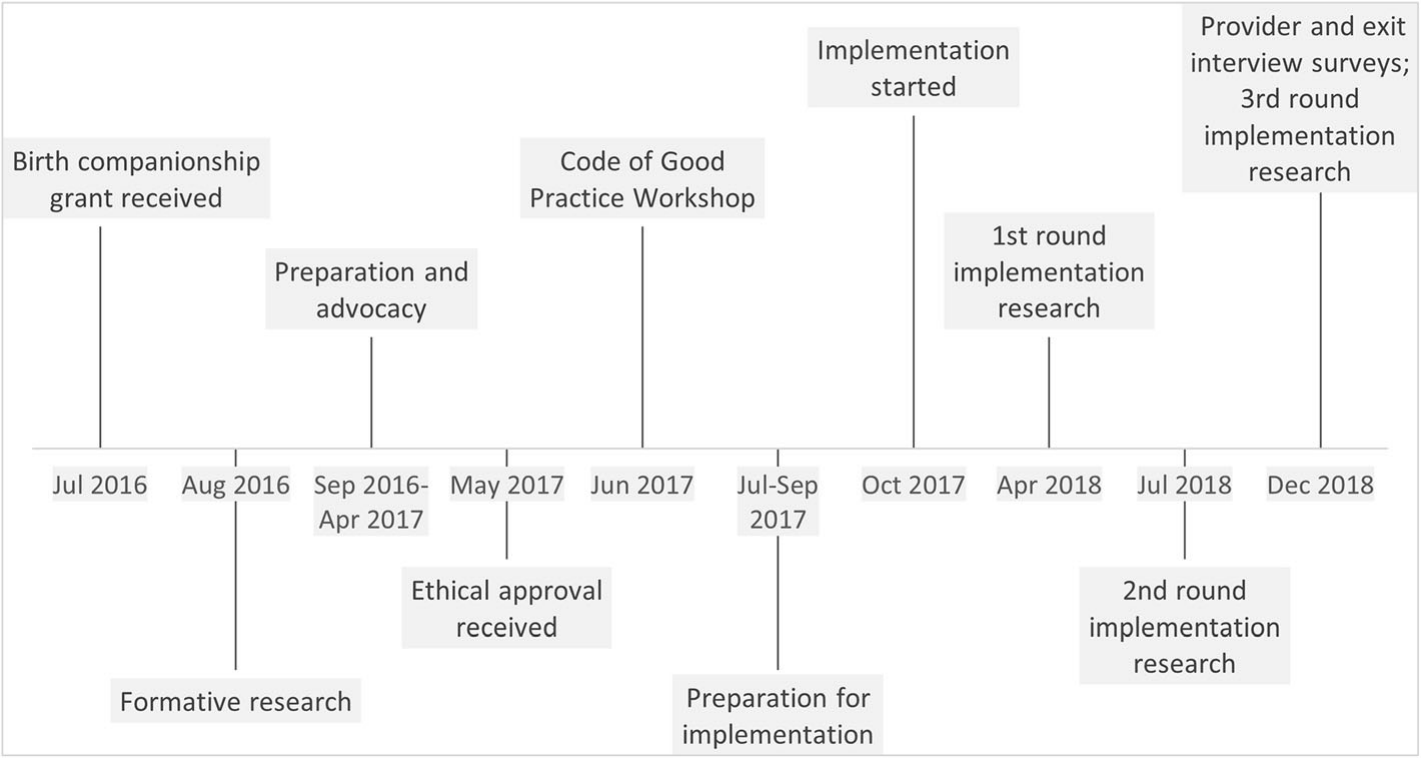
Timeline of the birth companionship pilot, July 2016–December 2018

Partners developed and implemented the birth companionship pilot between July 2016 and December 2018. Intervention and comparison sites were 15 health facilities (hospitals and health centers) that had been upgraded and supported by partners to provide good-quality comprehensive EmONC, including obstetric surgery. Birth companionship was implemented in 9 intervention sites, including 1 district hospital (urban) and 8 health centers (7 rural and 1 urban), which were selected based on facility layout. The remaining project facilities, including 2 hospitals and 4 health centers, were designated as comparison sites (Fig. 1).

### Development phase (Fig. 2)

Partners used a participatory approach to design the project and to ensure that the introduction of birth companionship responded to the needs and concerns of women and health providers, and strengthened rather than stressed relationships between communities and the health system [30]. Partners conducted formative research before the pilot to gain a deep understanding of the perceptions and norms around childbirth and birth companionship and to identify potential barriers to and facilitators for implementation. Thirty-one key informant interviews and seven focus group discussions were conducted with women who had recently delivered at facilities or at home, health care providers and administrators, community leaders, and traditional birth attendants. The responses were mixed. Women and providers could both imagine some benefits. Women thought that companions could help support them during labor, and providers saw the value of companions helping them with their workload. On the other hand, providers raised concerns about introducing someone new into the birthing “experience.” They mentioned concerns about privacy, limited space, infection prevention, fear of companions conducting unsafe practices, accountability, interpersonal relations and the potential to increase their workload. Women shared many of these concerns but were most vocal about auditory and visual privacy and a worry that other women’s companions might gossip about how they handled childbirth.

**Fig. 2:**
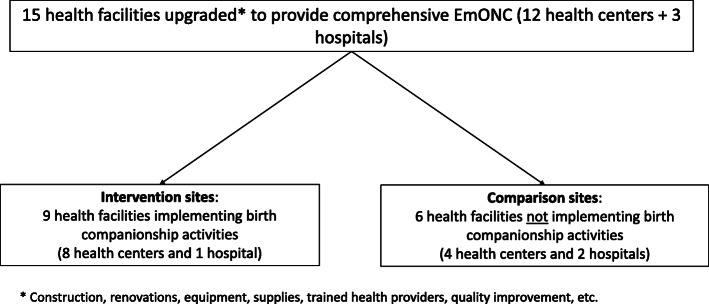
Pilot intervention and comparison sites. * Construction, renovations, equipment, supplies, trained health providers, quality improvement, etc.

Thamini Uhai and AMDD presented lessons from the formative research to a group of diverse stakeholders (i.e., community members, health providers, local officials) in a 4-day workshop during which a “Code of Good Practice” was developed. The Code of Good Practice identified the rights (e.g., right to human dignity, right to protection), roles (e.g., ensuring that women are not left alone, comforting, soothing and encouraging women during labor and delivery), responsibilities, and limitations (e.g., not giving medication, not touching medical instruments, not performing any medical procedure) of birth companions in the Kigoma context [31]. Participants defined 2 types of birth companions: (a) “desired birth companions” (DBC) selected by women during pregnancy and brought from their home or village to the facility; and (b) “on-call birth companions” (OBC) selected by communities and based at intervention facilities. OBCs were an option for women who did not bring a companion to facilities at the time of labor and for women who preferred a companion who was a non-family member/friend. All birth companions were female due to limited space and layout of the maternity ward and in respect of women’s privacy.

Pregnant women in intervention sites were informed during antenatal care visits and in community meetings that they could choose to have a DBC of their choice or have an OBC assigned to them. DBCs received 2 orientation sessions and a badge that allowed them entrance into the maternity ward; OBCs (3 per health center, 6 per hospital) received two days of training and monthly supportive supervision. Orientation covered topics such as: roles and responsibilities (e.g., providing continuous emotional, informational and practical support), techniques to reduce women’s stress and make them more comfortable (e.g., encouraging women with kind words, singing and praying, offering to hold their hands), infection prevention standards, and limitations to their role during labor and delivery (e.g., not managing the delivery, not administering medications). DBCs were also instructed to provide support to women at home during pregnancy and to the woman and her baby when they returned home (Table 1).

**Table 1:** Status, training, distribution, and compensation of desired and on-call birth companions

	Desired birth companions	On-call birth companions
**Selection/ recruitment**	Selected by women during their pregnancies.	Candidates nominated by communities and village committees and then selected by health facility management teams in consultation with Thamini Uhai.
**Recognition as “birth companion”**	Recognized and allowed to enter the labor room when they presented a special identification badge, which they received following two sessions of orientation.	Recognized as “on-call birth companions” once they completed training.
**Orientation and training**	• Nurses and community health workers provided orientation during antenatal clinics at pilot facilities and community health workers provided orientation in communities. • Orientation covered: women’s roles as birth companions, what birth companions are not allowed to do, patients’ rights and how birth companions could prevent infecting themselves, the women they accompany, other women in the maternity ward and newborns. • Received a refresher at time of labor/delivery and received tips from on-call birth companions on nonmedical comfort measures.	• Thamini Uhai trained OBCs over 2 days, covering the benefits of continuous support during childbirth and reviewing the Code of Good Practice including their roles and responsibilities, what they were not allowed to do and infection prevention practices. • OBCs also received additional training on nonmedical comfort measures.
**Availability and coverage**	Not applicable	• 3 companions selected and trained in each intervention health center. Due to a very large caseload, the intervention hospital had 6 companions. • A roster ensured 24/7 coverage at facilities.
**Compensation**	None from project	Received a monthly stipend, mobile phones with closed user group connection, monthly airtime recharge, uniform, and on-call/night shift allowance.

Responding to the concerns voiced during the formative research, existing labor/delivery rooms were renovated and divided into individual rooms with full partitions to address privacy concerns. Infection concerns were addressed through the addition of hand-washing stations, uniforms for OBCs, use of sanitized footwear in the maternity ward, and training on the proper handling and disposal of waste. Lastly, to ensure providers and government officials understood the benefits of companionship and supported the pilot, the Thamini Uhai team conducted several orientation meetings with them on a rolling basis.

### Implementation phase

Quality of care and adherence to the Code of Good Practice were assessed throughout implementation. Thamini Uhai conducted in-person monthly supportive supervision and training visits to support birth companions; visits consisted of observations, review of monthly data and discussions on how to improve birth support for women. AMDD also conducted focus group discussions and in-depth interviews with women, birth companions and health providers at key points during implementation; these findings were used to quickly address any problems and to document important lessons. In particular, supervision visits and implementation research ensured that companions were providing continuous support to the women they were accompanying, that there was good communication between health providers and companions, and that companions were not performing medical tasks. Midway through implementation, a U.S.-based certified doula and childbirth educator facilitated refresher trainings on nonmedical comfort measures for OBCs, as well as labor ward in-charges and reproductive and child health in-charges, from the 9 intervention sites. The training covered the benefits of continuous labor support, the connection between reducing stress and better birth outcomes, and demonstration of various nonmedical comfort techniques (e.g., breathing exercises, mobility, changing positions, singing, massage) using locally available resources.

Ongoing community engagement activities were also carried out including a mass media campaign featuring radio ads and interpersonal communication, supported by flyers, posters, billboards, community events and a 12-part radio magazine show. In addition, 49 community health workers were trained to promote birth companionship during antenatal care and household visits, and at community events.

### Data sources and analysis

We used 5 data sources to monitor and evaluate the pilot activities: (a) routine pilot monitoring data (quantitative); (b) implementation research focus group discussions and interviews (qualitative); (c) women’s exit interviews (quantitative); (d) provider interviews (quantitative); and (e) external pregnancy outcome data collected annually (quantitative). Ethical approval to conduct and evaluate this pilot was received from the National Institute for Medical Research in Tanzania. Written consent was obtained from all participants who were interviewed; verbal consent was received from all focus group discussion participants.

### Routine data collection

Woman-level data were collected from facility maternity registers in intervention sites including whether a birth companion was present at any point during labor and delivery and if she was an OBC or DBC. In addition, women were asked questions by OBCs to document the companion’s relationship to the mother (e.g., OBC, sister, mother, and friend). These data were collected in routine monthly reports and analyzed in Excel.

### Implementation research

The team employed qualitative data collection techniques throughout implementation, collecting data from the 9 intervention sites at 3 different time periods (April, July and December 2018; though not all sites were visited each time). In total, AMDD researchers conducted 21 focus group discussions with women of reproductive age who had given birth with a birth companion and 56 in-depth interviews with a range of stakeholders from the community, facility, district and regional teams, and among project staff, to explore experiences and perceptions of the pilot. Focus group discussion and interview participants were purposively sampled (*Using Questionnaire A, B, C, D and F: Implementation research Guides A, B, C, D and F*)

. Data were audio recorded, transcribed, translated to English and analyzed beginning with a set of a priori codes, allowing additional codes to emerge. Inter-coder reliability was established by team members independently coding the same 2 transcripts, and then deliberation of the codes until consensus was reached. NVivo 11 software which was used to code all transcripts into themes and subthemes; narrative scripts were produced with supporting quotes for emphasis and clarification.

### Providers and women exit interview surveys

Facility-based interview surveys were conducted with women who had just given birth and their providers in intervention and comparison sites during the last month of the pilot (Dec. 1 to 21, 2018). Interviews were conducted to (a) document the prevalence of companionship during labor, delivery and postpartum; and (b) describe women’s and providers’ satisfaction with the pilot, and experiences and perceptions of companionship, including quality of care.

All health workers who provided delivery care during the data collection period were interviewed, including: (a) clinicians (medical doctors, specialists, assistant medical officers, clinical officers, assistant clinical officers, and clinical assistants); (b) nurses/midwives; and (c) other staff such as medical attendants and maternal and child health aides. Women were eligible if they were 15 to 49 years of age, delivered at the facility during the data collection period, and had the capacity to understand and respond to questions. Women were approached at the time of discharge; all women approached for inclusion agreed to participate.

Interviewers administered face-to-face questionnaires in Kiswahili *(Questionnaire E and G*: *Client Birth Companion Questionnaire* and *Provider Birth Companion Questionnaire)* that had been initially developed as part of surveys conducted in 2016 and April/May 2018 [32] and amended to add birth companionship questions. All exit interviewers were hired from outside of the facility and trained in quantitative interview techniques and ethical considerations. The key variables used for sample size calculations included companion in labor, companion at the time of birth, and women’s satisfaction. At least 250 women were needed in each sub-group (intervention and comparison health facilities) to detect 10% relative mean differences in variables of interest, with 90% power and an alpha = 0.05, assuming a standard deviation of 10.

**Fig. 3:**
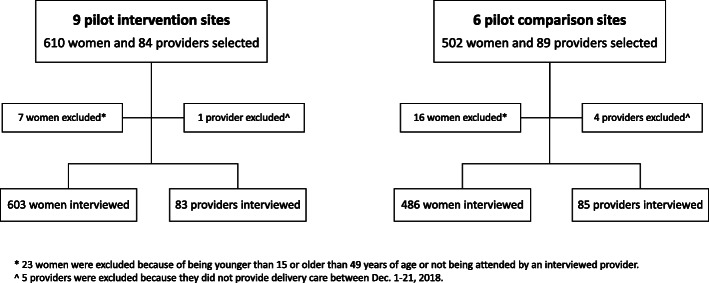
Interviews with women and their providers at the pilot intervention and comparison sites Source: exit interviews and providers survey. * 23 women were excluded because of being younger than 15 or older than 49 years of age or not being attended by an interviewed provider. ^ 5 providers were excluded because they did not provide delivery care between Dec. 1 and 21, 2018

Included in the analysis were: (a) data from providers who had cared for at least 1 woman who completed an interview (*N* = 168; 5 excluded for not providing delivery care); and (b) data from women whose provider was also interviewed (*N* = 1089; 23 excluded due to age or provider not interviewed). Descriptive univariate analyses were performed by site status (intervention/comparison) and facility type (hospital/health center). Analyses were conducted in Stata 14.1. An unpaired Student’s t test was used to identify differences in key variables by intervention and comparison sites; a value of *p* < 0.05 was considered statistically significant (Fig. 3).

### Pregnancy outcomes monitoring surveys

Four Pregnancy Outcomes Monitoring Surveys (POMS)—in 2013, 2016, 2018 and 2019—were conducted in the course of the overall project, collecting retrospective individual-level outcome data on all births that occurred in all health facilities in Kigoma with a minimum of 90 deliveries per year [6, 8, 33]. Using a package of standard tools, CDC/DRH teams obtained comprehensive maternal and newborn health information from labor and delivery registers, operating theater registers, admission and discharge registers, nurses’ report books, and other facility documents. These data were used to compute standard EmONC and maternal and newborn health outcome indicators and track changes over time. For the birth companionship pilot, the POMS data were also used to identify any substantial differences between outcomes of women who delivered during 2 15-month periods (pre-implementation: July 2016–September 2017, and implementation: October 2017–December 2018) in the intervention and comparison sites. POMS mortality indicators included: direct obstetric case fatality rate; facility maternal mortality ratio; intrapartum stillbirth rate; and pre-discharge neonatal mortality rate. Statistical significance of the change in mortality rates and ratios between pre-intervention and intervention periods was evaluated separately for intervention and comparison health facilities using a 2-proportion z-test.

Findings from all 5 data sources are used to show whether birth companionship was feasible and acceptable to women and health providers, and to learn if birth companionship improved women’s experience of care, improved the quality of maternal health services, and/or improved health outcomes.

## Results

### Birth Companionship utilization

Routine monitoring found that 82% of women delivering at intervention sites over the 15 months of implementation had a companion. This was similar to the exit interviews that found that 83% of women delivering at intervention sites had a companion during labor, at the time of birth, or during the postpartum period (Tables 2 and 3). Use of birth companions increased over time, from 59% in October 2017 to 83% in December 2018. At the health center level, 91% of women delivering had a birth companion; while uptake at the intervention hospital was lower (65%). Most women (69%) who had a birth companion learned about the pilot during antenatal care. Other women found out when they arrived at the facility (22–29%) or from a friend or family member (15–18%).

**Table 2:** Utilization of birth companions during implementation

	Facilities	Time period	Number of Women	Any birth companion^c^ N (%)	For women with birth companions	
					DBC %	OBC %
Births in health facilities^a^	9 intervention sites	October 2017–December 2018	16,465	13,551 (82.3)	28.2	71.8
Women interviewed^b^	9 intervention sites	December 2018	603	501 (83.1)	55.1	44.9
Women interviewed^b^	6 comparison sites	December 2018	486	100 (20.6)	100.0	NA

*Abbreviations:* *DBC* Desired birth companion; *OBC* On-call birth companion; *NA* Not applicable

^a^ Source: Routine monitoring data

^b^ Source: exit interviews

^c^ Had a DBC or OBC during labor, at the time of birth, or postpartum

**Table 3:** Companionship during labor, at the time of birth and postpartum by site status

Reported Companionship	Women in intervention sites	Women in comparison sites
	***N*** = 603%	*N* = 486%
Had a companion during at least one period of time: labor, birth or postpartum	83.1	20.6
Had a companion during labor (% yes)	76.5	6.2
Had a companion at the time of birth (% yes)	67.8	2.3
Had a companion during the postpartum period (% yes)	56.9	19.1

Source: exit interviews, December 2018

Among women who had companions, 72% had OBC support (Table 2). Use of OBCs was highest in the intervention hospital (86%) and the one urban health center (96%). Focus group participants said they thought that many women used OBCs because: they did not know they could bring a female companion from home; the DBC they selected was not available at the time of delivery; they did not have someone to bring from home; their DBC did not have the required badge; or they preferred to use an OBC.

Use of DBC support increased over time. In the first month of implementation (October 2017), only 7% of women with companions used DBCs (routine monitoring data). By December 2018, 55% of women with companions used DBCs (Table 2). Throughout implementation, a third of women delivering with companions in health centers (33%) were supported by DBCs, while 14% of women delivering with companions in the intervention hospital used DBCs (routine monitoring data). DBCs in health centers and hospitals were most often female family members (most frequently a mother-in-law, own mother, or sister) or friends (routine monitoring data). When considering whom to select, women in focus group discussions said they wanted someone “close” to them, with whom they felt comfortable, including feeling that they could ask for help, even with embarrassing things like using the bathroom. A “trustworthy” person was most often described as someone who would “keep secrets,” and not tell anyone else in the community how the woman behaved during labor. Many women also described choosing someone who lived nearby, who would be helpful or “sharp,” and not too old, but old enough to have had enough personal childbirth experience to be helpful.

In December 2018, 83% of women interviewed in intervention sites had a companion, including 77% during labor, 68% at the time of delivery, and 57% in the postpartum period. DBCs were present at all stages of childbirth whereas OBCs were present primarily during labor and delivery. In comparison sites, only 6% had a companion in labor, 2% at the time of delivery, and 19% in the postpartum period; one in five women delivering in comparison sites (21%) had a companion during at least one period of time (Table 3).

### Types of Support provided by birth companions

Exit interviews indicated that birth companions provided emotional, practical and informational support to women during labor and delivery and in the postpartum period. During labor, women most commonly reported that companions: gave them advice/instructions (61%); comforted them with kind words, singing, prayer, etc. (57%); gave them fluids to drink (50%); and stayed by their side for the majority of time (43%). OBCs more commonly comforted the women (74% OBCs vs. 50% DBCs), stayed by women’s side for the majority of time (68% OBC vs. 33% DBC) and communicated with staff (56% OBC vs. 33% DBC), while DBCs more commonly gave women fluids to drink (44% OBC vs. 53% DBC) and food to eat (10% OBC vs. 24% DBC).

During delivery, companions provided similar types of support as they did during labor and again we see that OBCs and DBCs provided slightly different types of support. During the postpartum period, birth companions gave women food to eat (89%), cleaned their clothes/linens (81%), and gave them fluids to drink (80%). Many DBCs also helped care for the newborn babies (64%) during the postpartum period (Table 4).

**Table 4:** Women’s reports of types of support given by birth companions at intervention sites

Support type	During labor %			At time of birth %			Postpartum %		
	DBC^a^	OBC^b^	Total	DBC^a^	OBC^b^	Total	DBC^a^	OBC^b^	Total
	*N* = 327	*N* = 134	*N* = 461	*N* = 204	*N* = 205	*N* = 409	*N* = 331	*N* = 12	*N* = 343
Cleaned my clothes/linens	15.9	3.7	12.4	13.7	2.3	8.3	82.8	33.3	81.1
Comforted me with kind words, singing, prayer, etc.	49.9	73.9	56.8	43.1	65.9	54.5	16.9	25.0	17.2
Communicated with family/husband	2.8	3.0	2.8	6.9	3.9	5.4	10.3	8.3	10.2
Communicated with staff	32.7	56.0	39.5	11.8	16.6	33.3	12.7	0.0	12.2
Gave me advice/instructions	51.7	83.6	61.0	51.5	67.3	59.4	18.4	33.3	19.0
Gave me fluids to drink	52.9	44.0	50.3	12.3	6.3	9.3	81.6	41.7	80.2
Gave me food to eat	23.6	9.7	19.5	6.4	2.3	4.7	90.0	50.0	88.6
Helped care for the baby	NA	NA	NA	NA	NA	NA	64.4	41.7	63.6
Helped me change position	6.7	17.2	9.8	5.9	19.0	12.5	6.0	0.0	5.8
Helped me walk around	23.9	30.6	25.8	5.4	6.8	6.1	6.7	8.3	6.7
Helped the staff	12.5	20.2	14.8	37.3	33.2	35.2	1.8	8.3	2.0
Other (help bathing, etc.)	1.5	1.5	1.5	0.5	1.5	1.0	8.5	8.3	8.5
Rubbed my back	7.0	14.2	9.1	28.4	38.1	14.2	1.2	8.3	1.5
Stayed by my side for majority of time	33.3	67.9	43.4	38.2	59.0	48.7	35.4	50.0	35.9
Supported breastfeeding	NA	NA	NA	NA	NA	NA	21.2	16.7	21.0

*Abbreviations:* ^a^*DBC* Desired birth companion; ^b^*OBC* On-call birth companion; *NA* Not applicable

Source: exit interviews, December 2018

Note: Additional elements of support reported by less than 10% of women who had a companion: helped ensure privacy; helped me reduce my pain (nonmedical); nothing; other (bathing, etc.); talked with me about family planning

Focus group and qualitative interview data indicated similar findings as exit interviews: birth companions comforted women, provided encouragement, reduced their worries and gave them hope, gave them massages, held their hand, and alerted providers when women needed help. The research also found that DBCs provided some support before the woman arrived at the facility for delivery, as well as after she returned home, whereas OBCs only provided support while the woman was at the facility. OBCs performed some tasks within the facility that desired companions did not do, such as promoting breastfeeding and advising on family planning (Table 5).

**Table 5:** Type of support provided by birth companions to women and providers

Support to women			Support to providers
Emotional	Informational	Practical/instrumental	
• Comfort/support • Encouragement (“sweet words”; will deliver safe by the will of God) • Reduce worries and give hope • Talk to women • Stay with women all the time • Help women feel “free” • Becoming a friend^a^	• Give advice • Remind women about hygiene • Translation • Educate women on breastfeeding, family planning, how to care for newborn^a^	• Massages • Help women exercise • Hold hand • Help women into bed • Support to urinate/vomit • Hold legs/shoulders during delivery • Support women to walk after delivery • Accompany to antenatal care^b^ • Encourage good diet • Help pack/carry things from home^b^ • Secure transport^b^ • Bring tea and food • • Clean women/help them get dressed after birth • Wash clothes • Carry the baby or belongings, and help women to postnatal ward after delivery • Help contact family • Carry things home once discharged^b^ • Cook for women^b^	• Alert providers when women need help/are ready to push • Keep women calm • Prepare delivery bed, clean bed after delivery • Help bring water/support providers to clean women • Reduce provider workload/give them time to do other things • Explain/reinforce provider instructions to women • Relay information to health providers (e.g., previous fistula) • Act as a link between providers and relatives • Welcome women to ward, collect antenatal care cards, show them beds^a^ • Help/remind nurses to take medical history and complete register^a^ • Do light cleaning tasks in labor ward^a^ • Hold trays and bring supplies to providers, sometimes fetching from other wards or store^a^ • Tell DBCs not to give local herbs or tell women to push too soon^a^ • Provide company/become a friend to providers^a^

*Abbreviations:* *DBC* Desired birth companion; *OBC* On-call birth companion

Source: Focus group discussions and key informant interviews (April, July and December 2018)

^a^OBC only

^b^DBC only

### Providers’ perspectives on birth Companionship

Quantitative surveys showed that providers at intervention sites were significantly more likely than providers at comparison sites to report supporting the use of birth companions (Table 6). All providers at intervention sites (*N* = 83; 100%) reported allowing companions during labor (vs. 15% of providers at comparison sites) and 87% reported allowing companions at the time of birth and postpartum (vs. 4% at the time of birth and 29% postpartum at comparison sites). Providers at intervention sites who reported not allowing companions at the time of birth (*N* = 11) cited privacy concerns (63%), and that they considered companionship distracting to the woman (36%). Providers at intervention sites who reported not allowing companions in the postpartum period (*N* = 11) reported the room was too small (73%) and they wanted to keep the room clean/reduce risk for infection (46%).

**Table 6:** Providers’ reports on attitudes toward birth companionship by site status

Attitude	Intervention sites	Comparison sites	Between-site comparison ***p***-value
	*N* = 83%	***N*** = 85%	
Reports allowing a companion in labor (% yes)	100.0	15.3	^a^ < 0.001
Reports allowing a companion at the time of birth (% yes)	86.8	3.5	^a^ < 0.001
Reports allowing a companion in the postpartum period (% yes)	86.8	29.4	^a^ < 0.001

^a^ An unpaired Student’s t test was used to identify differences in key variables by intervention and comparison sites; a value of *p* < 0.05 was considered statistically significant

Source: providers survey, December 2018

Providers at intervention sites who reported allowing companionship during labor or at the time of birth said they did so because companions help the provider with their workload (65 and 58%, respectively), tell the provider if there is a change in the woman’s status or a problem (64 and 42%, respectively), provide emotional support (54 and 54%, respectively), and help a woman feel more comfortable (52 and 47%, respectively), among other responses. In the postpartum period, providers in intervention sites reported allowing companions because they get the woman what she needs (74%), tell the provider if the woman’s condition changes (68%), and help care for the baby (68%). Providers who reported allowing companionship reported companions were “very helpful” (83–92%) and improved their ability to give good-quality care (86–93%) (Table 7).

**Table 7:** Providers’ perceptions of birth companionship at intervention sites among providers who allowed companionship

Opinion	During labor *N* = 83%	At time of birth ***N*** = 83%	Postpartum *N* = 83%
**Why do you allow a woman to have a companion?**			
*Helps provider with workload*	65.1	58.3	27.8
*Tells provider if change or problem (woman)*	63.9	41.7	68.1
*Gets woman what she needs*	55.4	40.3	73.6
*Gives woman emotional support*	54.2	54.2	29.2
*Helps woman feels more comfortable*	51.8	47.2	26.4
*Gives woman advice*	28.9	34.7	22.2
*Allows provider to be with other women*	25.3	18.1	20.8
*Facility policy allows it*	18.1	27.8	9.7
*Government policy allows it*	1.2	0.0	0.0
*Other*	3.6	2.8	2.7
*Helps care for the baby*	NA	NA	68.1
*Helps with breastfeeding*	NA	NA	26.4
*Tells provider if baby change/problem*	NA	NA	55.6
**Would you say that allowing companions has been satisfying or dissatisfying for you as a provider?**			
*Very satisfying*	92.8	87.5	88.9
*A little satisfying*	7.2	8.3	8.3
*Neither satisfying nor dissatisfying*	0.0	4.2	2.8
*A little dissatisfying*	0.0	0.0	0.0
*Very dissatisfying*	0.0	0.0	0.0
**Would you say that allowing companions has been helpful or unhelpful for you as a provider?**			
*Very helpful*	91.6	83.3	90.3
*A little helpful*	8.4	9.7	6.9
*Neither helpful nor unhelpful*	3.6	6.9	2.8
*A little unhelpful*	1.2	0.0	0.0
*Very unhelpful*	0.0	0.0	0.0
**Would you say that allowing companions has made it harder, has not changed your ability or has improved your ability to give good quality care?**			
*Improved ability to give good quality care*	92.8	86.1	88.9
*Has not changed ability to give good quality care*	7.2	13.9	11.1
**Would you say that allowing companions has not met your expectations, met your expectations, or exceeded your expectations?**			
*Exceeded expectations*	25.3	20.8	16.7
*Met expectations*	71.1	76.4	80.6
*Did not meet expectations*	3.6	2.8	2.8

Source: providers survey, December 2018

Focus group discussions and key informant interviews with providers also showed consensus across all stakeholder groups that birth companions made providers’ work easier, and that providers valued this support. The principal reason for this was that companions stayed by women’s sides and called providers when they were needed. Furthermore, all the various types of support that companions provided to women and providers are tasks that would otherwise fall on the providers; having someone else in the facility to help them therefore reduced their workloads. Providers also described ways in which they felt that birth companions made providers’ work easier: by helping women better understand providers’ instructions; helping to reassure and calm women; and helping women with movement (e.g., changing positions, going to the bathroom). Occasionally, companions interpreted between providers and women who were not able to speak Kiswahili fluently. For the most part, companions provided the types of support expected of them. But, in a few occasions, providers reported that companions did tasks that were outside companions’ scope of work, as defined by the Code of Good Practice [31], such as light cleaning around labor and delivery, but that the companions felt were necessary to make women more comfortable (e.g., if the woman vomited) (Table 5).

Health workers also appreciated when companions provided information that was relevant to the woman’s clinical management, such as whether she took traditional medicines before admission or had a repaired fistula. Some providers also valued companions for their more indirect roles; for example, providers reported that having companions present meant they would be less likely to be blamed for poor outcomes or accused of mistreating women, because companions could attest to what happened and could explain how hard providers tried to help women and their babies. In addition, 1 provider and 2 OBCs described how providers appreciated the company that OBCs gave them, especially during night shifts. (Key informant interviews).

### Women’s opinions on birth Companionship

The majority of women interviewed at intervention sites in December 2018 were very satisfied with having a companion during labor (97%), at the time of delivery (96%), and postpartum (99%). Most women at the intervention sites also reported that the presence of a companion improved their labor, delivery and postpartum experience (82–97%) (Table 8). Focus group discussions and interviews also showed that women were very happy to have had a birth companion.

**Table 8:** Women’s satisfaction with and perceptions of birth companionship among women with companions at intervention sites

Opinion	During labor *N* = 461%	At time of birth ***N*** = 409%	Postpartum ***N*** = 343%
**Level of satisfaction with having a companion**			
*Very satisfied*	97.2	96.3	99.1
*A little satisfied*	2.2	3.7	0.3
*Neither satisfied nor dissatisfied*	0.4	0.0	0.3
*A little dissatisfied*	0.2	0.0	0.3
*Very dissatisfied*	0.0	0.0	0.0
**Influence of having a labor companion on experience**			
*Made experience better*	82.4	86.1	96.8
*Did not change experience*	17.4	13.9	3.2
*Don’t know*	0.2	0.0	0.0
**Influence of having a companion on future use of the facility**			
*Increased chance of returning*	92.4	99.3	95.3
*Did not change chance of returning*	6.9	8.1	4.7
*Don’t know*	0.7	0.7	0.0
**Influence of having a companion on recommending the facility to family and friends**			
*Increased chance of recommending facility*	92.2	92.4	94.5
*Did not change chance of recommending facility*	7.8	7.6	5.3
*Decrease chance of recommending facility*	0.0	0.0	0.3

Source: exit interviews, December 2018

*“My birth companion comforted me, massaged me at the back and told me to be patient, God is with you, you will get better soon, she was telling me sweet words and then she was singing gospel songs for me so I gave birth without feeling any pain.*” —Focus group discussion: Women with DBCs

*“To be honest, having a birth companion makes you feel really good, we really thank you for bringing us the birth companion because the previous births you were staying there in the labor room alone, the minute you feel like you are pushing is when you call the nurse to help you now, right now at least you just heard they have called for, if she has been called for emergency the birth companion is working to call the nurse, so you really feel comfortable when you are with a birth companion.” —*Focus group discussion: Wom*e*n with DBCs

### Women’s overall experience of care

When comparing intervention with comparison sites, women reported that providers at intervention sites were significantly more likely to respond to women who called for help compared to providers in comparison sites (*p* = 0.003), to interact in a friendly way (*p* < 0.001), to greet them respectfully (*p* < 0.001), to try to make them more comfortable (*p* = 0.003), and to encourage them to have a companion (*p* < 0.001). However, women at intervention sites were significantly less likely to feel comfortable asking questions than women at comparison sites (*p* < 0.001). While uncommonly reported, women at intervention sites were less likely to report experiencing emotional abuse (defined as being spoken to in an angry or condescending way that made the woman feel bad about herself, degraded, embarrassed or sad; *p* = 0.023) and physical abuse (defined as being hit, slapped, pushed, pinched, kicked or receiving any other type of physical force; *p* = 0.006) from providers (Table 9).

**Table 9:** Women’s experience of elements of respectful maternity care by site status

Experience	Intervention Sites *N* = 603%	Comparison Sites ***N*** = 486%	Between-site comparison ***p***-value^a^
**Did the provider […**]?			
*Attend if woman calls for help*	98.7	96.1	0.003
*Interact in a friendly way*	97.7	91.2	< 0.001
*Greet respectfully*	97.0	88.3	< 0.001
*Pay close attention throughout delivery*	87.6	89.5	0.160
*Try to make more comfortable*	85.6	79.2	0.003
*Introduce themselves*	41.8	43.4	0.295
*Encourage you to have a companion*	46.6	11.9	< 0.001
**Did the woman […]?**			
*Feel comfortable to ask questions*	65.8	90.7	< 0.001
**Experience abusive behavior from provider**			
*Emotional abuse*	1.3	3.1	0.023
*Physical abuse*	0.0	1.0	0.006

^a^ An unpaired Student’s t test was used to identify differences in key variables by intervention and comparison sites; a value of *p* < 0.05 was considered statistically significant

Source: exit interviews, December 2018

During the focus group discussions, women described their experiences receiving respectful care, which they attributed to the introduction of birth companions. As was found in the exit interviews, women in focus group discussions reported a faster response time from the health providers, since their companions were able to alert the providers immediately when changes in health status occurred. This was discussed as a measure to ensure that women received the timely care that they needed, as well as to provide peace of mind to the women, who knew that someone was looking out for them. Having someone to go get the nurse when a woman was ready to push was mentioned by all informant types as being one of the main reasons they appreciated the intervention.

Women also talked about being treated well and spoken to kindly by the nurses in the presence of their birth companions. Women said the nurses were “kind” and “good” and “received me well.” One woman in a focus group discussion suggested that she felt less pain during labor because of the sweet words that the nurse said to her. Women spoke of this change in 2 ways: some described an overall shift in the facility culture which led to happier nurses, whereas others guessed that it had more to do with fear that the birth companion would ultimately report the nurse.

Lastly, when birth companions talked about their role and how it may have affected providers’ treatment of the women they were supporting, they most often mentioned being outsiders and therefore serving as “witnesses” to providers’ behavior and being advocates for women’s rights and wishes. As one DBC said, her presence simply *“helps the nurse remember her responsibilities when she sees you.”* She further explained that she felt the nurses worked harder because of her simply being there:

*“ … because at that time when they see you that you are close they try to work hard, because they know that this person is with a companion if I do wrong she is investigating, I may find myself given a bad or good report, so you find a nurse is careful at that moment because when she sees me she is trying her best at her ability.” —* Key informant interview: DBC

Women in intervention sites were significantly more likely to report being “very satisfied” with the care they received (*p* < 0.001), and that the staff were “very kind” to them (*p* < 0.001) and “very encouraging” (*p* < 0.001). When asked what they were most satisfied with, women at intervention sites were more likely to report that staff were kind in the way they were giving treatment (*p* < 0.001), used encouraging words (*p* < 0.001), and were attentive to their needs (*p* < .001) (Table 10).

**Table 10:** Women’s satisfaction with care by site status

Satisfaction with care	Intervention sites ***N*** = 603%	Comparison sites ***N*** = 486%	Between-site comparison ***p***-value^a^
**How would you rate your overall level of satisfaction with the care you received?**			
*Very satisfied with care*	93.4	81.3	< 0.001
*A little satisfied/neither satisfied nor dissatisfied/a little dissatisfied/very dissatisfied*	6.6	18.7	
**How would you rate the staff’s kindness?**			
*Very kind*	94.0	80.0	< 0.001
*A little kind/neither kind nor unkind/a little unkind/very unkind*	6.0	20.0	
**How would you rate the staff’s encouragement?**			
*Very encouraging*	94.4	81.9	< 0.001
*A little encouraging/neither encouraging nor discouraging/a little discouraging/very discouraging*	5.6	18.1	
**What about your care were you satisfied with?**			
*Staff was kind in the way they treated me*	83.6	54.9	< 0.001
*Staff used encouraging words*	59.4	40.3	< 0.001
*Staff came when I called*	47.4	44.4	0.163
*Staff was attentive to my needs*^a^	26.4	16.7	< 0.001
*Staff stayed with me*	27.2	15.2	0.001
**What about your care were you dissatisfied with?**			
*Nothing*	77.9	52.7	< 0.001
*Staff did not allow me to have a birth companion*	0.2	7.0	< 0.001
**Would you return to the facility for care in the future? (% yes)**	99.0	97.3	0.029
**Would you recommend this facility to friends and family? (% yes)**	99.7	96.1	< 0.001

^a^ An unpaired Student’s t test was used to identify differences in key variables by intervention and comparison sites; a value of *p* < 0.05 was considered statistically significant

Source: exit interviews, December 2018

Interestingly, one of the first things most women mentioned in the focus group discussions was their improved sense of privacy and confidentiality due to the renovations made. Though this was not due to the incorporation of birth companions alone, it is important to note that while women valued the inclusion of a supportive companion, the importance of preserving their privacy and dignity behind walls or partitions was something that had great importance to their birth experience.

Almost all women across intervention and comparison sites said they would return to the facility for care in the future (99 and 97%, respectively). Women at intervention sites were significantly more likely to report that they would recommend the facility to friends and family compared to women at comparison sites (< 0.001) (Table 10).

### Outcome indicators

Between October 2017 and December 2018, a total of 16,789 women gave birth in the 9 intervention facilities and 13,424 women gave birth in the comparison facilities. Compared to the 15 months prior to the implementation of the companionship project (July 2016 to September 2017), the number of deliveries increased by 2% in intervention sites and decreased by 6% in comparison sites (Table 11).

**Table 11:** Birth outcomes before and during pilot implementation by site status

	Intervention Sites (***N*** = 9)				Comparison Sites (*N* = 6)			
Indicator	Before Pilot^a^	During Pilot^b^	% Change	Significance level of % change^c^	Before Pilot^a^	During Pilot^b^	% Change	Significance level of % change^c^
Deliveries in health facilities	16,410	16,789	2.3%	NA	14,291	13,424	−6.1%	NA
Live births in health facilities	16,189	16,618	2.6%	NA	14,003	13,196	−5.8%	NA
Obstetric complications treated	3199	3369	5.3%	NA	3873	3598	−7.1%	NA
Direct obstetric maternal deaths	51	41	−19.6%	NA	74	64	−13.5%	NA
Intrapartum stillbirths	287	226	−21.3%	NA	294	231	−21.4%	NA
Pre-discharge neonatal deaths	239	243	1.7%	NA	367	323	−12.0%	NA
Direct obstetric case fatality rate (including deaths due to first trimester complications)	1.6	1.2	−23.7%	0.198	1.9	1.8	−6.9%	0.675
Institutional MMR (per 100,000 live births)	315.0	246.7	−21.7%	0.244	528.5	485.0	−8.2%	0.615
Intrapartum stillbirth rate per 1000 births	17.2	13.1	−23.6%	0.003	19.7	16.6	−15.8%	0.046
Pre-discharge neonatal death rate per 1000 live births	14.8	14.6	−1.0%	0.916	26.2	24.5	−6.6%	0.370

*Abbreviations:* *MMR*  Maternal Mortality Ratio; *NA* Not applicable

Source: Pregnancy Outcomes Monitoring Surveys: direct obstetric case fatality rate; facility maternal mortality ratio; intrapartum stillbirth rate; and pre-discharge neonatal mortality rate were calculated for the 2 15-month periods, in the intervention and comparison sites

^a^ July 2016–September 2017

^b^ October 2017–December 2018

^c^Significance of the difference between the two periods was tested using two proportion z test

Maternal and neonatal mortality declined in both intervention and comparison sites. While declines were generally larger in the intervention sites than in the comparison sites, changes from pre-intervention to intervention periods were not statistically significant in either group of health facilities. However, there was a significant decline in the intrapartum stillbirth rate in both intervention and comparison sites (from 17.2 to 13.2 per 1000 and from 20.1 to 16.9 per 1000, respectively) (Table 11).

## Discussion

The birth companionship pilot in Kigoma shows that introducing and implementing a birth companionship program into the government health system in a rural region of Tanzania is feasible. Despite initial hesitation and concern, birth companionship became a reality for more than 80% of women delivering at the 9 intervention facilities, and women and providers alike felt that it improved the quality of care.

Concerns about privacy, crowding in the maternity ward and introduction of infection, which were identified in the formative research and cited in other studies [24–26], were overcome in this pilot through: involving health providers, community members and government officials at the design phase and in developing the Code of Good Practice [31]; close implementation support to facilities; and minor maternity ward renovations which provided auditory and visual privacy to women and their companions. Very few health providers, government officials or women in intervention sites maintained these concerns by the end of the project. There were no identified problems with infections as a result of having birth companions in intervention sites. Further, there was a 24% decline in direct obstetric case fatality rate and a 22% decline in the institutional maternal mortality ratio, though not statistically significant.

Uptake of this new intervention over 15 months was quite rapid in large part because of the use of OBCs who were offered to most women arriving at intervention facilities. The use of DBCs took longer because of the orientation process required to receive a DBC badge and because some women were unaware of the process for bringing a companion from home. However, over time, as more women became aware of the birth companionship initiative through various communication strategies, and through minor adjustments to the DBC orientation process, the use of DBCs increased and in the last month of implementation, surpassed the use of OBCs.

As in other studies, women were highly satisfied with having companions and most women reported that the presence of a companion improved their birth experience [24, 25, 27, 34]. What was unique in this pilot was the use and documentation of how 2 types of companions worked in rural government facilities. Women had positive encounters with both OBCs and DBCs but the types of support that they provided, while similar, were not identical. Both types of companions comforted women with kind words, singing or prayer, stayed by the woman’s side for the majority of time during childbirth, and provided other types of emotional and practical support during labor (like helping women walk around and giving them fluids to drink) and at the time of birth (such as giving advice and instructions). However, because OBCs were more experienced, had more training (including on nonmedical comfort measures), understood their roles better, were more familiar with the health facility setting, and had established relationships with health providers at the facilities, their roles differed: OBCs were more likely to provide continuous support, give advice/instructions, and communicate with health providers. DBCs, on the other hand, were able to provide support to women at home, arrange transport, help women with their newborns, give women food to eat and fluids to drink, and clean clothes and linens after the birth. OBCs were not present as much during the postpartum period because after the delivery, especially at high-volume facilities, they would often be called to accompany newly admitted women.

These different roles are important to understand and consider for future planning. DBCs are more cost effective, more likely to be sustained, and with good-quality orientation, have the potential to expand the types of support they are able to provide to women during pregnancy, labor, delivery and postpartum, including at home following discharge. Offering OBCs to all women who deliver at facilities, however, can help get the intervention accepted and launched rapidly, while changed rules about birth companionship and DBC orientation processes are shared with communities. A group of OBCs is also easier to train than an ever-expanding number of DBCs in a community, OBCs’ gained experience from accompanying many women over time can be shared with DBCs, and OBCs’ expertise and confidence may be desirable to some women. Clearly there are benefits to having both OBCs and DBCs together in facilities, as was piloted in Kigoma. One potential, more cost-effective compromise could be to place in facilities trained birth companion coordinators who have experience in a range of culturally and context appropriate comfort measure techniques, and who could provide support to DBCs and serve as a resource for communities on the benefits of companionship.

Health providers in intervention sites were very positive about the addition of birth companionship. From their perspective, birth companions had the dual role of providing emotional support to women and relieving certain aspects of health providers’ jobs, some of which birth companions were not meant to be doing but felt were necessary or urgent to make women more comfortable (e.g., fetching water and doing light cleaning). In a more ideal situation, nurse-midwives would be able to provide a more supportive role during childbirth. But, with current staffing levels and other health system constraints, nurse-midwives are rarely able to spend time providing continuous emotional support to women in their facilities. Further, with increasing caseloads and stagnant staffing levels, nurses and others in the maternity ward are stretched and unable to assist and closely monitor all women at the same time. Therefore, health providers were happy to have birth companions because they relieved providers of some aspects of their heavy workloads and ultimately, they felt that the introduction of birth companions improved their ability to provide good-quality care. However, despite birth companions’ critical role providing support to women during childbirth, birth companionship should not be thought of as a standalone solution to structural problems in the health system, particularly related to human resource shortages, and strong accountability systems need to be in place to help prevent the potential for birth companions to take on (or be given) more roles or tasks than are allowed.

The environment and culture of facilities with birth companionship appear to have changed in positive ways. Women reported that health providers were more responsive, treated them more respectfully, tried to make them more comfortable and were kinder than in comparison sites. Whether this was due to positive changes that were introduced and promoted by the birth companions or because health providers perceived birth companions as witnesses to their behavior and actions, the change is positive. This is a very important finding and provides the field with an example of an intervention that contributes to humanizing maternity care, ensuring women are treated with dignity, and improving quality of care. Another important finding is that women greatly appreciated the full partitions that were constructed in the labor and delivery rooms because they improved their sense of privacy and confidentiality. While not directly related to the birth companionship intervention, this is important to note, and partial partitions are now being included in Tanzanian guidelines for respectful care and maternity ward design. One surprising finding was that women who had a companion in either intervention or comparison sites felt less comfortable asking questions than women without companions. This is an issue that should be explored in future studies.

Over the course of implementation, the number of women delivering at intervention facilities increased slightly. Having more women deliver at health facilities offering good quality care is in line with the government’s objectives [2–4]. The findings that most women reported being satisfied with their facility delivery, would recommend the facility to other women, and would return for future deliveries, indicates that changes have occurred that may lead to sustained increases in utilization (absent other changes around facility functioning and quality of care). Birth companionship, therefore, has the potential in settings such as Kigoma to increase institutional deliveries while improving women’s experience of care.

Intrapartum stillbirth rate was the only outcome indicator that declined significantly in both intervention and comparison sites. Declines in the case fatality rate, institutional maternal mortality ratio and the pre-discharge neonatal mortality rate were larger in the intervention sites than in the comparison sites though comparisons between the 2 groups of sites were not subject to statistical testing. While other clinical quality improvement initiatives were in progress at the same time and comparison facilities also saw declines, it is possible that the increased attention that women received during childbirth from birth companions, who were able to quickly alert health providers when they were needed, could have contributed to these meaningful improvements. However, relying on companions to alert health providers in this way could become problematic if companions are blamed for missing danger signs or are blamed for adverse events; continuous support provided by companions is not the same as close monitoring by nurse-midwives and clinicians—it should be supplementary.

This study is not without limitations that could have affected the results. One limitation relates to selection of intervention and comparison sites. Intervention sites were not randomly assigned but were selected based on the size and layout of maternity wards. In addition, the intervention sites included only 1 hospital whereas the comparison sites included 2, which suggests the possibility of some key differences between the groups of sites before the introduction of birth companionship. Another limitation was the challenge of social desirability bias when women and providers were asked about the quality of services. We tried to minimize this bias by recruiting interviewers who were not associated with the intervention facilities, conducting interviews outside of health facilities and by having Tanzanian social scientists from outside the region facilitate focus group discussions and conduct in-depth interviews. We feel that our use of multiple data sources—focus group discussions, in-depth interviews, and exit interviews—to assess respondents’ satisfaction decreased our risk for this bias and provides more confidence in our results. Despite these limitations, the experiences documented using both quantitative and qualitative methods in our relatively large, government supported pilot provides the field with important implementation guidance and strong evidence in support of implementing birth companionship in places such as Kigoma.

Important implementation lessons were learned in this pilot that should be applied to future birth companionship initiatives in Tanzania and elsewhere. These include the value of: involving all stakeholders in the design and implementation phases; creating a set of guidelines which defined the roles and limitations of companions; incorporating implementation research into the project design; and using multiple communication strategies to ensure health providers and communities understand the intervention and its benefits. Challenges encountered during implementation of this pilot were identified and managed by a dedicated team of implementers and researchers who had strong relationships in the region and this is likely to have contributed to the pilot’s overall success. Future birth companionship projects may consider: focusing more on DBCs and how to deepen their roles during pregnancy and the postpartum period, adding more emphasis on nonmedical comfort measures, and coming up with creative ways to provide privacy before facility renovations are made. Going forward, it will also be important to learn how birth companionship can be implemented in settings without a well-funded and dedicated implementation team and how it can be integrated into routine government health services. A follow-up project to the Kigoma pilot with a focus on sustainability is forthcoming.

## Conclusion

The introduction of birth companionship in participating facilities was feasible and well accepted by health providers, government officials and most importantly, women who delivered at those facilities. Birth companions provided women with continuous emotional, informational and practical support during childbirth and that appears to have contributed to women having better birth experiences in health facilities. Birth companionship also seemed to improve the environment of the maternity wards overall. Based on findings from this pilot, birth companionship would be a beneficial option for all women giving birth in health facilities in Tanzania.

## Supplementary Information

**Additional file 1.**

## Data Availability

The data generated and analyzed for this study are available from Thamini Uhai, CDC and AMDD on reasonable request.

## Abbreviations

AMDD: Averting Maternal Death and Disability Program
CDC/DRH: U.S. Centers for Disease Control and Prevention, Division of Reproductive Health
DBC: Desired birth companion
EmONC: Emergency obstetric and newborn care
MoHCDGEC: Tanzania Ministry of Health, Community Development, Gender, Elderly and Children
OBC: On-call birth companion
POMS: Pregnancy Outcomes Monitoring Surveys

## References

[1] Ministry of Health, Community Development, Gender E and C (2016). Tanzania Demographic and Health Survey and Malaria Indicator Survey 2015–2016.

[2] Ministry of Health Gender E and C. The National Strategic Plan to Improve Reproductive, Maternal, Newborn, Child & Adolescent Health in Tanzania (2016–2020) - One Plan II (with revised baseline and targets). Dar es Salaam; 2018.

[3] Ministry of Health Gender E and C. The National Road Map Strategic Plan to Improve Reproductive, Maternal, Newborn, Child & Adolescent Health in Tanzania (2016–2020) One Plan II, Tanzania. Dar es Salaam; 2015.

[4] Ministry of Health Gender E and C-(MoHCDGEC). Health Sector Strategic Plan July 2015–June 2020 (HSSP IV). Dar es Salaam; 2015.

[5] National Bureau of Statistics, Ministry of Finance TUR of T. 2012 Population and Housing Census: Tanzania Basic Demographic and Socio-Economic Profile [Internet]. Dar es Salaam; 2014. Available from: https://www.nbs.go.tz/index.php/en/census-surveys/population-and-housing-census/164-2012-phc-tanzania-basic-demographic-and-socio-economic-profile.

[6] U.S. Centers for Disease Control and Prevention (CDC). Reducing maternal mortality in Tanzania: Pregnancy outcomes findings from Kigoma Region, Tanzania - September 2018. Atlanta; 2019.

[7] MoHCDGEC (2011). Tanzania Demographic and Health Survey 2010.

[8] U.S. Centers for Disease Control and Prevention (CDC). Reducing Maternal Mortality in Tanzania: Pregnancy Outcomes Findings from Kigoma Region, Tanzania - Selected Findings 2016. Atlanta; 2016.

[9] U.S. Centers for Disease Control and Prevention (CDC). 2016 Kigoma Reproductive Health Survey - Kigoma Region, Tanzania. Atlanta; 2016.

[10] Bohren MA, Hunter EC, Munthe-Kaas HM, Souza JP, Vogel JP, Gülmezoglu AM (2014). Facilitators and barriers to facility-based delivery in low- and middle-income countries: a qualitative evidence synthesis. Reprod Health.

[11] Mselle LT, Moland KM, Mvungi A, Evjen-Olsen B, Kohi TW (2013). Why give birth in health facility? Users’ and providers’ accounts of poor quality of birth care in Tanzania. BMC Health Serv Res.

[12] Freedman LP, Kujawski SA, Mbuyita S, Kuwawenaruwa A, Kruk ME, Ramsey K (2018). Eye of the beholder? Observation versus self-report in the measurement of disrespect and abuse during facility-based childbirth. Reprod Health Matter.

[13] Kruk ME, Kujawski S, Mbaruku G, Ramsey K, Moyo W, Freedman LP. Disrespectful and abusive treatment during facility delivery in Tanzania: a facility and community survey. Health Policy Plan [Internet]. 2018;33(1):e26–e33 [cited 2020 mar 3]. Available from: http://www.ncbi.nlm.nih.gov/pubmed/29304252. 10.1093/heapol/czu079 29304252

[14] Mselle LT, Kohi TW, Dol J. Humanizing birth in Tanzania: a qualitative study on the (mis) treatment of women during childbirth from the perspective of mothers and fathers. BMC Pregnancy Childbirth. 2019;19(1):231. Available from. 10.1186/s12884-019-2385-5. 10.1186/s12884-019-2385-5 PMC6612108 31277609

[15] Kruk ME, Paczkowski M, Mbaruku G, De Pinho H, Galea S. Women’s preferences for place of delivery in rural Tanzania: A population-based discrete choice experiment. Am J Public Health. 2009;99(9):1666–72. 10.2105/AJPH.2008.146209. 10.2105/AJPH.2008.146209 PMC2724466 19608959

[16] MoHCDGEC. Human Resource for Health and Social Welfare Strategic Plan 2014-2019. Dar es Salaam; 2014.

[17] Mselle LT, Kohi TW, Dol J. Barriers and facilitators to humanizing birth care in Tanzania: findings from semi-structured interviews with midwives and obstetricians. Reprod Health. 2018;15(1):137. Available from: doi: 10.1186/s12978-018-0583-7. 10.1186/s12978-018-0583-7 PMC6092851 30107840

[18] Dynes MM, Binzen S, Twentyman E, Nguyen H, Lobis S, Mwakatundu N, et al. Client and provider factors associated with companionship during labor and birth in Kigoma Region, Tanzania. Midwifery. 2019;69:92–101. Available from: doi: 10.1016/j.midw.2018.11.002. 10.1016/j.midw.2018.11.002 PMC11019777 30453122

[19] Downe S, Finlayson K, Oladapo O, Bonet M (2018). Gülmezoglu AM. What matters to women during childbirth: A systematic qualitative review. PLoS One.

[20] World Health Organization. Standards for Improving Quality of Maternal and Newborn Care in Health Facilities. Geneva; 2016. Available from: https://apps.who.int/iris/bitstream/handle/10665/249155/9789241511216-eng.pdf;jsessionid=573855BE349FE7542315983B59D9B177?sequence=1.

[21] World Health Organization. WHO Recommendations on Health Promotion Interventions for Maternal and Newborn Health. Geneva; 2015. Available from: https://apps.who.int/iris/bitstream/handle/10665/172427/9789241508742_report_eng.pdf;jsessionid=657D49F4A9D87EA1E7CD28954898411A?sequence=1. 26180864

[22] World Health Organization. WHO Recommendations for Augmentation of Labour [Internet]. Geneva; 2014. Available from: https://apps.who.int/iris/bitstream/handle/10665/112825/9789241507363_eng.pdf?sequence=1. 25506951

[23] Bohren MA, Hofmeyr GJ, Sakala C, Fukuzawa RK, Cuthbert A. Continuous support for women during childbirth. Cochrane Database Syst Rev. 2017;(7) [cited 2019 Feb 14]. Available from. 10.1002/14651858.CD003766.pub6. 10.1002/14651858.CD003766.pub6 PMC6483123 28681500

[24] Bohren MA, Berger BO, Munthe-Kaas H, Tunçalp Ö. Perceptions and experiences of labour companionship: a qualitative evidence synthesis. Cochrane Database Syst Rev. 2019;(3):18 [cited 2019 May 15]. Available from: http://doi.wiley.com/10.1002/14651858.CD012449.pub2. 10.1002/14651858.CD012449.pub2 PMC6422112 30883666

[25] Afulani P, Kusi C, Kirumbi L, Walker D (2018). Companionship during facility-based childbirth: results from a mixed-methods study with recently delivered women and providers in Kenya. BMC Pregnancy Childbirth.

[26] Kabakian-Khasholian T, Portela A (2017). Companion of choice at birth: factors affecting implementation. BMC Pregnancy Childbirth.

[27] Banda G, Kafulafula G, Nyirenda E, Taulo F, Kalilani L (2010). Acceptability and experience of supportive companionship during childbirth in Malawi. BJOG An Int J Obstet Gynaecol.

[28] Brown H, Hofmeyr GJ, Nikodem VC, Smith H, Garner P (2007). Promoting childbirth companions in South Africa: a randomised pilot study. BMC Med.

[29] Maimbolwa MC, Sikazwe N, Yamba B, Diwan V, Ransjö-Arvidson A-B (2001). Views on Involving a Social Support Person During Labor in Zambian Maternities. J Midwifery Womens Health.

[30] Sohng SSL. Participatory Research and Community Organizing. J Sociol Soc Welf. 1996;23(4) Available from: https://scholarworks.wmich.edu/cgi/viewcontent.cgi?article=2378&context=jssw.

[31] Thamini Uhai. Code of good practice for birth Companionship: Kigoma, Tanzania. Dar es Salaam; 2017. Available from: https://www.thaminiuhai.or.tz/node/182

[32] U.S. Centers for Disease Control and Prevention (CDC). Client and Provider Experiences with Facility-Based Delivery and Reproductive Health Care Services in Kigoma Region, Tanzania, April–May, 2018. Atlanta; 2020.

[33] U.S. Centers for Disease Control and Prevention (CDC). Reducing Maternal Mortality in Tanzania: Pregnancy Outcomes Findings from Kigoma Region, Tanzania - Selected Findings 2013. Atlanta; 2014.

[34] Kabakian-Khasholian T, Portela A (2017). BMC Pregnancy Childbirth.

