# Supplementary material for: Birth companionship in a government health system: a pilot study in Kigoma, Tanzania
Source: BMC Pregnancy Childbirth. 2021 Apr 16;21:304. doi: 10.1186/s12884-021-03746-0 (PMC8052747; doi:10.1186/s12884-021-03746-0)
Supplement: Supplementary file 1 — Additional file 1. [file 12884_2021_3746_MOESM1_ESM.zip › A. Implementation Research IDI_AdministratorsR4.doc]

**Implementation Research IDI Guide**

**Administrators: RMO, DMO**

General Information:

| District Name | Ward | Village/Facility Name |
| --- | --- | --- |
|  |  |  |
| Date of Interview | Time of Commencement | Time of Completion |
|  |  |  |
| Name of the facilitator | Name of Reporter | Name of observer |
|  |  |  |

**ACCEPTABILITY**

1. What have your staff members told you about the experiencesof having birth companions in their facility?
2. Are there any administrative tasks that you have had to take on to help support the introduction of the birth companion program?
   a. IF YES:
      i. What are they?
      ii. How much work has it been for you?

**ROLES OF THE BC**

3. Have you observed first-hand the birth companions in action in any of the health facilities?
a. IF YES:
i. What type of assistance did the birth companions offer to health providers? (What roles did BCs play?)
ii. What type of assistance did the birth companion offer to the women during labor and delivery?

**BENEFITS**

4. From the time of program implementation, what benefits have you:
a. Observed?
b. Heard about?
5. What existing opportunities/ strengths exist within facilities that can better support the birth companionship program?
6. How do you think the renovations and additional items purchased (such as infection prevention materials, uniforms, etc.) have impacted women’s experiences during labour and delivery?
c. How, if at all, have the renovations and additional supplies impacted the providers’ ability to provide good quality care?
7. Are you familiar with the ‘Code of Good Practice’ that defines the roles of birth companions?
d. IF YES:
   i. Do you feel the ‘Code of Good Practice’ has been helpful in actually defining the roles of birth companions?
   ii. Is there anything you would add or change in the ‘Code’?

**CHALLENGES**

8. Are there any challenges that you experienced, as an administrator, because of the presence of birth companions in the selected communities (either through observation or through reports from health facilities)?
   a. IF YES:
      i. What were they? [Probe if anything has been done to address these challenges?]
      ii. Are there any suggestions of dealing with these challenges?
9. What are some of the concerns that you might have regarding the implementation and continuation of the program?
a. Ask are there any challenges regarding thework environment
b. Health providers that might make implementation difficult?

[Probe if anything has been done to address these challenges for both a and b]

10. What are some specific things that the BC did that you feel she should not have done(observed and heard about from staff members)?

**LESSONS LEARNT/SCALE-UP**

11. Would you like to expand birth companionship to the other health facilities in your district / region? Why/why not?
12. Would you recommend implementation of the birth companionship program to other regions/ districts in Tanzania? Why/why not?
13. What have you learned from the program that might be useful for possible scale up? (PROBE: The strengths, obstacles, opportunities, threats -SWOT)
14. How do think the birth companion program can be sustained at facility, district, regional and national level?
15. Do you think that the birth companion program should be continued in the facilities?
    a. IF YES: How can you as health administrators ensure continuation of the program?
16. Are there any otherareas that you feel need to be improved regarding birth companions?
    a. IF YES:What are these areas?
