# Supplementary material for: Birth companionship in a government health system: a pilot study in Kigoma, Tanzania
Source: BMC Pregnancy Childbirth. 2021 Apr 16;21:304. doi: 10.1186/s12884-021-03746-0 (PMC8052747; doi:10.1186/s12884-021-03746-0)
Supplement: Supplementary file 1 — Additional file 1. [file 12884_2021_3746_MOESM1_ESM.zip › B. Implementation Research IDI_Community LeadersR4.doc]

**Implementation Research IDI Guide**

**Community leaders (VEO, WEO, Village Chairman, religious leaders)**

General Information:

| District Name | Ward | Village/Facility Name |
| --- | --- | --- |
|  |  |  |
| Date of Interview | Time of Commencement | Time of Completion |
|  |  |  |
| Name of the facilitator | Name of Reporter | Name of observer |
|  |  |  |

**ACCEPTABILITY**

1. Are you aware of the birth companion program in the surrounding facilities?
   a. What are your opinions about the idea of women having a birth companion by their sides during labor and deliveryin the health facility?
   b. Do you have any role as a community leader in the birth companion program?
      i. IF YES: How are you involved in the program implementation as a community leader?
   c. Normally, in most families, how are decisions surrounding childbirth made, how are these birth companions chosen? [PROBE on who determines how these decisions are made and why? Is it according to individuals, household/family, community (as culture and society dictates), village government (in instances where repeat DBCs/TBAs are pre-selected for the community)
2. In your opinion, is the community aware of the birth companion program?
   a. IF YES:
      i. When and how did the community come to know about this program?
      ii. In your opinion, what do you think the community feels about having birth companions in the health facilities?
      iii. To the best of your knowledge, how do you think birth companions will be selected?
3. Are you aware of the renovations made to the hospitals/health centers?
   a. IF YES:What have you heard about the renovations?

**ROLES**

4. To the best of your knowledge, what type of assistance are the birth companions offering to the women in facilities? [PROBE on psychosocial support if mentioned: How the BC provide this support, for example if a respondent says encourage, or comforting, explore further on how they *actually* did that]
5. To the best of your knowledge, what type of assistance are the birth companions offering to health providers in facilities?
6. Are you familiar with the birth companionship ‘Code of Good Practice’?
   a. IF YES:
      i. Was the document helpful in explaining the roles of birth companions throughout pregnancy and childbirth?
      ii. Is there anything about the current document that needs to be added or changed?

**BENEFITS**

7. Have you heard about or experienced any benefits of having a birth companion during labor and delivery ever since the program was started in October 2017?
   a. IF YES: What are they?

**CHALLENGES**

8. What are some of the concerns that the community has voiced regarding the current program of birth companions? And are there any suggestions for dealing with these challenges?[PROBE: If anything has been done to address these challenges and how were they addressed]

**LESSONS LEARNT/SCALE-UP**

9. Based on what you understand and have heard from the community, would you recommend implementation of the birth companionship program to other facilities in Tanzania? Why/why not?
10. What have you learned from the program that might be useful for possible scale up? (PROBE:What worked?What didn’t?What adaptations were made?)
11. Do you think the community accepts the birth companion program in the health facilities?
    a. IF YES: How can the community ensure continuation of the program to maintain these benefits?
12. What are other areas that you feel need to be improved regarding birth companions?
