# Supplementary material for: Birth companionship in a government health system: a pilot study in Kigoma, Tanzania
Source: BMC Pregnancy Childbirth. 2021 Apr 16;21:304. doi: 10.1186/s12884-021-03746-0 (PMC8052747; doi:10.1186/s12884-021-03746-0)
Supplement: Supplementary file 1 — Additional file 1. [file 12884_2021_3746_MOESM1_ESM.zip › C. Implementation Research Guide _ProvidersR4.doc]

**Implementation Research IDI Guide**

**Health Providers**

General Information:

| District Name | Ward | Village/Facility Name |
| --- | --- | --- |
|  |  |  |
| Date of Interview | Time of Commencement | Time of Completion |
|  |  |  |
| Name of the facilitator | Name of Reporter | Name of observer |
|  |  |  |

1. Are women bringing birth companions into your facility to support them throughout labour?
   a. IF YES:
      i. What has been your experience with having a birth companion support women during labour?
      ii. What type of support do birth companions offer women during labour?
2. Are birth companions supporting women during delivery? (Why/why not)?
   a. IF YES:
      i. What has been your experience with having a birth companion support women during delivery?
      ii. What type of support do birth companions offer women during delivery?

[PROBE: To better understand psychosocial support provided, explore further on *how* the BC provided comfort, encouragement, soothed the women, made them feel not alone, gave the women confidence- what did the BCs do exactly to illustrate the above]

3. What type of assistance/support/help do you get from the birth companion? (What roles did BCs play?)[ PROBE i. Explore further on what each of these BCs did or did not do exactly to offer support to women and providers]

ii. Probe on how the providers worked together with BCs to make the woman cooperative, for example, when it is time to push]

a. Is there a difference in the type of assistance or support between the on-call birth companion and the desired birth companion a woman brings from home?

[PROBE on other roles that OBCs undertook that go beyond assisting the women, for example, orienting DBCs, supervising, assessing and monitoring DBCs competency. Were OBCs asked to assume a supervisory/gatekeeper role over DBCs, or did they take it upon themselves to play these roles?]

4. In your opinion, what were some of the benefits of having a birth companion around during labor and delivery? (PROBE i. For health providers and for women)

ii. Probe further when respondents state that women provide certain information to BCs that they normally withheld from providers and clarify on whether withholding of information is intentional or if she did not think it was not relevant

5. Are there any particular behaviors or things that the birth companion did that you feel she should not have done? Can you state some of them? (PROBE: lack of support, how they interacted with providers)
6. What are your views on the current health facility environment regarding accommodation of a birth companion? (PROBE: physical space, policy, supervision, remuneration, workload, etc.)
7. Did you find the birth companionship ‘Code of Good Practice’ helpful in defining the roles of companions in the facility?
   a. Is there anything you would add or change in the ‘Code’?
8. In your opinion, have the renovations that were made to accommodate birth companions in the health facilities been helpful? (PROBE: How/why?)
   a. Have the additional items purchased to support the implementation been helpful? (PROBE: What was most useful and why? What was unnecessary?)
9. Are there any challenges that you experienced because of the presence of a birth companion? What where they? [PROBE: whether anything has been done to address the challenges] And are there any suggestions of dealing with these challenges?
10. Would you recommend implementation of the birth companionship program to other facilities in Tanzania? Why/why not?
11. Are there other areas that you feel need to be improved regarding birth companions?
