# Supplementary material for: Birth companionship in a government health system: a pilot study in Kigoma, Tanzania
Source: BMC Pregnancy Childbirth. 2021 Apr 16;21:304. doi: 10.1186/s12884-021-03746-0 (PMC8052747; doi:10.1186/s12884-021-03746-0)
Supplement: Supplementary file 1 — Additional file 1. [file 12884_2021_3746_MOESM1_ESM.zip › Client Post Delivery BIRTH COMPANION_ENGLISHR4.doc]

| **CLIENT POST-DELIVERY BIRTH COMPANION EXIT INTERVIEW – KIGOMA REGION, TANZANIA** | | | |
| --- | --- | --- | --- |
|  | Date of Interview: | A. DAY………………………………………………………….…….[ __\|__ ]  B. MONTH…………………………………………………..…..….[ __\|__]  C. YEAR………………………………………………..….[ __\|__\|__\|__ ] |  |
|  | Time at Start of Interview: | 24 HOUR CLOCK…………………………..…….……[ __\|__:__\|__ ] |  |
|  | Interviewer Code: | …………………………………………………….….……..[ __\|__\|__\|__ ] |  |
|  | Supervisor Code: | …………………………………………………….……………..……...[ __\|__] |  |
|  | Unique Provider ID: | ……………………………..……..…………[ 4\|__\|__\|__\|__\|__\|__ ] |  |
|  | Facility Name: | _________________________________________________ |  |
|  | Facility Code: | …………………………………………………….…….…..[ __\|__\|__\|__ ] |  |
|  | Facility Type:  ***CIRCLE FACILITY TYPE*** | HOSPITAL……………………………………………………………………….1  HEALTH CENTER…………………………..………………………………..2 |  |
|  | District:  ***CIRCLE DISTRICT*** | BUHIGWE……………………………………………………………………….1  KIBONDO…………………………………………………..………………....2  KAKONKO…………………………………..………………………..………..3  KASULU…………………………………………………………………..……..4  KIGOMA…………………………………………………………………….…..5  UVINZA………………………………………………………………………..…6 |  |
|  | Time at End of Interview: | 24 HOUR CLOCK…………………………………..… [ __\|__:__\|__ ] |  |
|  | Result Code:  ***CIRCLE RESULT*** | COMPLETED……………………………………………………………………1  INCOMPLETE (SPECIFY RETURN DATE)______________...2  POSTPONED (SPECIFY DATE)______________________...3  OTHER (SPECIFY) _____________________________......7  REFUSED…………………………….…………………………………..……..9 |  |
|  | Date of Data Entry: | A. DAY………………………………………………………………. [ __\|__ ]  B. MONTH……………………………………………………...…. [ __\|__]  C. YEAR………………………………………………..….[ __\|__\|__\|__ ] |  |
|  | Data Entry Clerk Code: | …………………………………………………………………..……... [ __\|__] |  |

| **INTRODUCTION AND CONSENT**  Hello. My name is ___________. I am working with the Ministry of Health, Community Development, Gender, Elderly and Children,ThaminiUhai, and AMCA. We are conducting a survey about health services in Kigoma. The information we collect will help the government to improve the quality of health services. The survey usually takes about 30 minutes. Your answers will be confidential and will not be shared with anyone except members of our survey team. You don’t have to participate, but we hope you will agree since your views are important. You will not benefit directly from participating. There are no risks to you, but you may feel uncomfortable with some questions. If I ask you any question you don’t want to answer, let me know and I will go on to the next question or you can stop the interview at any time. Do you have any questions? May I begin now? | | |
| --- | --- | --- |
| Signature of interviewer: _______________________________________ Date: __________________________ | | |
| Result of Consent | RESPONDENT DOES NOT AGREE TO BE INTERVIEWED……0 RESPONDENT AGREES TO BE INTERVIEWED…………………..1 | END |

| **PARTICIPANT ELIGIBILITY / SCREENING QUESTIONS** | | | |
| --- | --- | --- | --- |
| **Number** | **Question** | **Response Options** | **Skip** |
| E1 | How old were you on your last birthday? | AGE IN YEARS…………………………………………………...[ __\|__ ]  DON’T KNOW / REMEMBER……………….………………………88 | END IF NOT 15-49 |
| E3A | Did you come to the facility for delivery services? | NO……………………………………………….………………….……………0  YES……………………………………………….………….……….………….1 | END |
| E5 | Where was your new baby born?  ***IF DELIVERED AT A DIFFERENT FACILITY, ASK WHERE AND RECORD THE NAME OF THE FACILITY*** | AT THIS FACILITY…………………………………….…………..……….1  AT A DIFFERENT FACILITY_______________________...2  AT A TRADITIONAL BIRTH ATTENDANT’S HOME…………..3  AT HOME………………….…………..…..…………….…………………..4  OTHER (SPECIFY) ____________________.………………….7 | END |

| **SECTION 1. LABOR AND DELIVERY CARE VISIT** | | | | | | | | | |
| --- | --- | --- | --- | --- | --- | --- | --- | --- | --- |
| **Now, I would like to talk to you about the labor and delivery services for which you came to this facility during this visit.** | | | | | | | | | |
| 1.1 | How long ago was your new baby born?  ***RECORD HOURS OR DAYS*** | A. HOURS……………………………………….……………….…1[ __\|__ ]  B. DAYS…………………………………………..……….………..2[ __\|__ ] | | | | | | |  |
| 1.1A | Was your baby alive at birth? A baby is considered live born if he/she makes any movement, cries, or attempts to breath, even if for only moments. | NO…………………………………………………………….…………....…..0  YES……………………………………………………………………………….1 | | | | | | | 1.1C |
| 1.1B | Is your baby alive now? | NO…………………………………………………………………………....…..0  YES………………………………………………..……………………………….1 | | | | | | |  |
| 1.1C | Did you deliver your baby vaginally? | NO……………………………………………………………………………..…..0  YES………………………………………………………………………………….1 | | | | | | | 1.1E |
| 1.1D | Was your baby born by a cesarean section (C/S)? By cesarean section, we mean, did you have surgery to remove your baby through your abdomen? | NO…………………………………………………..…………………….…..…..0  YES…………………………………………………………………………...…….1 | | | | | | | 1.1F |
| 1.1E | Did the baby deliver spontaneously or with the assistance of a vacuum or forceps? | SPONTANEOUS VAGINAL BIRTH………………………………………1  VACUUM ASSISTED VAGINAL BIRTH………………………………..2  FORCEPS ASSISTED VAGINAL BIRTH…………………………………3  DON’T KNOW……………………………………………………………..…..8 | | | | | | |  |
| 1.1F | Did the facility’s maternity ward have walls between you and the other women when you were in labor (before the baby was born)? | NO……………………………………………………………………………..…..0  YES………………………………………………………………………………….1 DON’T KNOW / REMEMBER………………….………………..………8 | | | | | | | 1.1H  1.1H |
| 1.1G | If yes, did the walls…?  **READ EACH QUESTION BELOW** |  | | | **NO** | | **YES** | **DK** |  |
|  | A. Increase your privacy related what you or others could hear | A. Privacy related to what could be heard | | | 0 | | 1 | 8 |  |
|  | B. Increase your privacy related to what you or others could see | B. Privacy related to what could be seen | | | 0 | | 1 | 8 |  |
|  | C. Make you feel more comfortable | C. Feel more comfortable | | | 0 | | 1 | 8 |  |
| 1.1H | Did the facility’s maternity ward have walls between you and the other women at the time of delivery? | NO……………………………………………………………………………..…..0  YES………………………………………………………………………………….1 DON’T KNOW / REMEMBER………………….………………..………8 | | | | | | | 1.3  1.3 |
| 1.1I | If yes, did the walls…?  **READ EACH QUESTION BELOW** |  | | | **NO** | | **YES** | **DK** |  |
|  | A. Increase your privacy related to what you or others could hear | A. Privacy related to what could be heard | | | 0 | | 1 | 8 |  |
|  | B. Increase your privacy related to what you or others could see | B. Privacy related to what could be seen | | | 0 | | 1 | 8 |  |
|  | C. Make you feel more comfortable | C. Feel more comfortable | | | 0 | | 1 | 8 |  |
| **LABOR COMPANION SECTION:**  Now I am going to ask you questions about having a companion / support person with you when you were in labor before the baby was born. A birth companion or support person may be someone you know and brought from the community who may or may not have received a birth companionship orientation and birth companionship ID badge, or it may be a woman wearing a grey uniform who was based at the facility and was by your side throughout labor. A facility staff such as a nurse or midwife is not considered a birth companion. | | | | | | | | | |
| 1.3 | Did you have a companion / support person in the room with you during labor? By labor, I mean during the time you were at the facility before the baby was born. | NO……………………………………………………………………………..…..0  YES………………………………………………………………………………….1 DON’T KNOW / REMEMBER………………….………………………..8 | | | | | | | 1.4M  1.4M |
| 1.4 | Who was your companion / support person(s) in labor?  ***DO NOT READ LIST***  ***MULTIPLE RESPONSES ALLOWED***  ***IF CLIENT SAYS A HEALTHCARE PROVIDER WAS HER COMPANION, CLARIFY IF IT WAS A COMPANION IN A GRAY UNIFORM. GO BACK TO 1.3 AND UPDATE IF NEEDED.*** |  | | | | **NO** | | **YES** |  |
|  |  | A. HUSBAND OR PARTNER | | | | 0 | | 1 |  |
|  |  | B. MOTHER | | | | 0 | | 1 |  |
|  |  | C. SISTER | | | | 0 | | 1 |  |
|  |  | D. MOTHER-IN-LAW | | | | 0 | | 1 |  |
|  |  | E. TRADITIONAL BIRTH ATTENDANT | | | | 0 | | 1 |  |
|  |  | F. NEIGHBOR | | | | 0 | | 1 |  |
|  |  | G. FRIEND | | | | 0 | | 1 |  |
|  |  | H. OTHER (SPECIFY) ____________________ | | | | 0 | | 1 |  |
|  |  | I. A COMPANION PROVIDED BY THE FACILITY (WITH THE GREY UNIFORM) | | | | 0 | | 1 |  |
| 1.4A | Who made the decision about who your companion / support person in labor would be?  ***DO NOT READ LIST***  ***MULTIPLE RESPONSES ALLOWED*** |  | | | | **NO** | | **YES** |  |
|  |  | A. CLIENT HERSELF | | | | 0 | | 1 |  |
|  |  | B. HUSBAND OR PARTNER | | | | 0 | | 1 |  |
|  |  | C. MOTHER | | | | 0 | | 1 |  |
|  |  | D. MOTHER-IN-LAW | | | | 0 | | 1 |  |
|  |  | E. SOMEONE ELSE IN OWN FAMILY (FATHER, SISTER, BROTHER, ETC) | | | | 0 | | 1 |  |
|  |  | F. SOMEONE ELSE IN HUSBAND’S FAMILY (FATHER-IN-LAW, SISTER-IN-LAW, ETC) | | | | 0 | | 1 |  |
|  |  | G. TRADITIONAL BIRTH ATTENDANT | | | | 0 | | 1 |  |
|  |  | H. VILLAGE LEADER | | | | 0 | | 1 |  |
|  |  | I. HEALTH PROVIDER AT FACILITY | | | | 0 | | 1 |  |
|  |  | J. OTHER (SPECIFY) ___________________ | | | | 0 | | 1 |  |
|  |  | K. DON’T KNOW | | | | 0 | | 1 |  |
| 1.4B | Was [INSERT NAME OF COMPANION(S)] the individual(s) who you preferred to have with you as a companion / support person in labor? | NO……………………………………………………………………………..…..0  YES………………………………………………………………………………….1 DON’T KNOW / REMEMBER………………….………………..………8 | | | | | | | 1.4D  1.4D |
| 1.4C | Why did you prefer to have [INSERT NAME OF PREFERRED COMPANION(S)] as a companion / support person during labor?  ***DO NOT READ LIST***  ***MULTIPLE RESPONSES ALLOWED*** |  | | | | **NO** | | **YES** | 1.4F |
|  |  | A. KNOWS ME WELL | | | | 0 | | 1 |  |
|  |  | B. EXPERIENCED WITH LABOR/DELIVERY | | | | 0 | | 1 |  |
|  |  | C. I AM NOT SHY AROUND HER/HIM | | | | 0 | | 1 |  |
|  |  | D. WILL GIVE ME ADVICE | | | | 0 | | 1 |  |
|  |  | E. WILL TREAT ME KINDLY | | | | 0 | | 1 |  |
|  |  | F. I TRUST HER/HIM | | | | 0 | | 1 |  |
|  |  | G. SHE/HE WILL NOT GOSSIP ABOUT ME | | | | 0 | | 1 |  |
|  |  | H. OTHER (SPECIFY) __________________ | | | | 0 | | 1 |  |
|  |  | I. DON’T KNOW | | | | 0 | | 1 |  |
| 1.4D | Who would you have preferred to have as your companion / support person(s) in labor?  ***DO NOT READ LIST***  ***MULTIPLE RESPONSES ALLOWED*** |  | | | | **NO** | | **YES** |  |
|  |  | A. HUSBAND OR PARTNER | | | | 0 | | 1 |  |
|  |  | B. MOTHER | | | | 0 | | 1 |  |
|  |  | C. SISTER | | | | 0 | | 1 |  |
|  |  | D. MOTHER-IN-LAW | | | | 0 | | 1 |  |
|  |  | E. TRADITIONAL BIRTH ATTENDANT | | | | 0 | | 1 |  |
|  |  | F. NEIGHBOR | | | | 0 | | 1 |  |
|  |  | G. FRIEND | | | | 0 | | 1 |  |
|  |  | H. OTHER (SPECIFY) ____________________ | | | | 0 | | 1 |  |
|  |  | I. A COMPANION PROVIDED BY THE FACILITY | | | | 0 | | 1 |  |
|  |  | J. DON’T KNOW | | | | 0 | | 1 | 1.4F |
| 1.4E | Why would you have preferred to have [INSERT NAME OF PREFERRED COMPANION(S)] as a companion / support person during labor?  ***DO NOT READ LIST***  ***MULTIPLE RESPONSES ALLOWED*** |  | | | | **NO** | | **YES** |  |
|  |  | A. KNOWS ME WELL | | | | 0 | | 1 |  |
|  |  | B. EXPERIENCED WITH LABOR/DELIVERY | | | | 0 | | 1 |  |
|  |  | C. I AM NOT SHY AROUND HER/HIM | | | | 0 | | 1 |  |
|  |  | D. WILL GIVE ME ADVICE | | | | 0 | | 1 |  |
|  |  | E. WILL TREAT ME KINDLY | | | | 0 | | 1 |  |
|  |  | F. I TRUST HER/HIM | | | | 0 | | 1 |  |
|  |  | G. SHE/HE WILL NOT GOSSIP ABOUT ME | | | | 0 | | 1 |  |
|  |  | H. OTHER (SPECIFY) __________________ | | | | 0 | | 1 |  |
|  |  | I. DON’T KNOW | | | | 0 | | 1 |  |
| 1.4F | How did you find out that this facility allows a companion / support person during labor?  **DO NOT READ LIST**  **MULTIPLE RESPONSES ALLOWED**  **PORBE: Anything else?** |  | | | | **NO** | | **YES** |  |
|  |  | A. UPON ARRIVAL AT THE FACILITY FOR DELIVERY CARE | | | | 0 | | 1 |  |
|  |  | B. DURING ANTENATAL CARE AT FACILITY | | | | 0 | | 1 |  |
|  |  | C. COMMUNITY HEALTH WORKER | | | | 0 | | 1 |  |
|  |  | D. FRIEND OR FAMILY MEMBER | | | | 0 | | 1 |  |
|  |  | E. RADIO ADVERTISEMENT | | | | 0 | | 1 |  |
|  |  | F. PRINT MATERIALS (FLYERS, POSTERS, BILLBOARD) | | | | 0 | | 1 |  |
|  |  | G. RELIGIOUS GATHERING | | | | 0 | | 1 |  |
|  |  | H. WOMEN’S ASSOCIATION MEETING | | | | 0 | | 1 |  |
|  |  | I. OTHER COMMUNITY MEETING | | | | 0 | | 1 |  |
|  |  | J. OTHER (SPECIFY) ___________________ | | | | 0 | | 1 |  |
|  |  | K. DON’T KNOW | | | | 0 | | 1 |  |
| 1.4G | What did your companion / support person do to support you during labor?  **DO NOT READ LIST**  **MULTIPLE RESPONSES ALLOWED**  **PROBE:** Anything else? |  | | | | **NO** | | **YES** |  |
|  |  | A. GAVE ME ADVICE OR INSTRUCTIONS | | | | 0 | | 1 |  |
|  |  | B. HELPED ME TO WALK AROUND | | | | 0 | | 1 |  |
|  |  | C. HELPED ME TO CHANGE POSITION | | | | 0 | | 1 |  |
|  |  | D. GAVE ME FOOD TO EAT | | | | 0 | | 1 |  |
|  |  | E. GAVE ME FLUIDS TO DRINK | | | | 0 | | 1 |  |
|  |  | F. RUBBED MY BACK | | | | 0 | | 1 |  |
|  |  | G. CLEANED MY CLOTHES OR LINENS | | | | 0 | | 1 |  |
|  |  | H. STAYED WITH ME BY MY SIDE FOR THE MAJORITY OF THE TIME | | | | 0 | | 1 |  |
|  |  | I. COMFORTED ME WITH KIND WORDS, SINGING, PRAYER, ETC | | | | 0 | | 1 |  |
|  |  | J. HELPED ME TRY THINGS TO REDUCE THE PAIN (NON-MEDICAL) | | | | 0 | | 1 |  |
|  |  | K. HELPED THE STAFF | | | | 0 | | 1 |  |
|  |  | L. COMMUNICATED WITH THE STAFF | | | | 0 | | 1 |  |
|  |  | M. COMMUNICATED WITH MY FAMILY/ HUSBAND | | | | 0 | | 1 |  |
|  |  | N. HELPED ENSURE PRIVACY | | | | 0 | | 1 |  |
|  |  | O. OTHER (SPECIFY) ___________________ | | | | 0 | | 1 |  |
|  |  | P. NOTHING | | | | 0 | | 1 |  |
|  |  | Q. DON’T KNOW | | | | 0 | | 1 |  |
| 1.4H | What would you have liked your companion / support person to do for you in labor that she did not do, if anything?  **DO NOT READ LIST**  **MULTIPLE RESPONSES ALLOWED**  **PROBE:** Anything else? |  | | | | **NO** | | **YES** |  |
|  |  | A. GIVE ME ADVICE OR INSTRUCTIONS | | | | 0 | | 1 |  |
|  |  | B. HELP ME TO WALK AROUND | | | | 0 | | 1 |  |
|  |  | C. HELP ME TO CHANGE POSITION | | | | 0 | | 1 |  |
|  |  | D. GIVE ME FOOD TO EAT | | | | 0 | | 1 |  |
|  |  | E. GIVE ME FLUIDS TO DRINK | | | | 0 | | 1 |  |
|  |  | F. RUB MY BACK | | | | 0 | | 1 |  |
|  |  | G. CLEAN MY CLOTHES OR LINENS | | | | 0 | | 1 |  |
|  |  | H. STAY WITH ME BY MY SIDE FOR THE MAJORITY OF THE TIME | | | | 0 | | 1 |  |
|  |  | I. COMFORT ME WITH KIND WORDS, SINGING, PRAYER, ETC | | | | 0 | | 1 |  |
|  |  | J. HELP ME TRY THINGS TO REDUCE THE PAIN (NON-MEDICAL) | | | | 0 | | 1 |  |
|  |  | K. HELP THE STAFF | | | | 0 | | 1 |  |
|  |  | L. COMMUNICATE WITH THE STAFF | | | | 0 | | 1 |  |
|  |  | M. COMMUNICATE WITH MY FAMILY/ HUSBAND | | | | 0 | | 1 |  |
|  |  | N. HELP ENSURE PRIVACY | | | | 0 | | 1 |  |
|  |  | O. OTHER (SPECIFY) ___________________ | | | | 0 | | 1 |  |
|  |  | P. NOTHING | | | | 0 | | 1 |  |
|  |  | Q. DON’T KNOW | | | | 0 | | 1 |  |
| 1.4I | A. Overall, would you say that having a companion / support person in labor was satisfying or dissatisfying? | DISSATISFYING….………………………..………………………………….1  SATISFYING…….…………………………..……………………..…..……..2  NEITHER DISSATISFYING NOR SATISFYING……..………………3  DON’T KNOW………………………….…………….………………….…..8 | | | | | | | 1.4J  1.4J |
|  | B. Would you say it was very […] or a little […]?   **USE ANSWER FROM 1.4I_AIN PLACE OF […] (SATISFIED OR DISSATISFIED)** | VERY DISSATISFYING……………………….………………….…………..1  A LITTLE DISSATISFYING….…………………………..……...………….2  A LITTLE SATISFYING…….………………………………………….…..…4  VERY SATISFYING…….………………………………………….………..…5  DON’T KNOW………………………………………………………………….8 | | | | | | |  |
| 1.4J | Would you say that having a companion /or support person in labor made your labor experience worse, did not change your labor experience, or made your labor experience better? | MADE LABOR EXPERIENCE WORSE……………………………..…..1  DID NOT CHANGE LABOR EXPERIENCE……………………….……2  MADELABOR EXPERIENCE BETTER………………………….………3  DON’T KNOW……………………………………………………….…………8 | | | | | | |  |
| 1.4K | Would you say that having a companion / support person in labor will decrease the chance that you will return to this facility in the future, not change the chance of returning to the facility, or increase the chance that you will return to the facility? | DECREASE CHANCE OF RETURNING…………………….…………..1  NOT CHANGE CHANCE OF RETURNING…………...………………2  INCREASE CHANCE OF RETURNING………………….………………3  DON’T KNOW………………………………………………….……………….8 | | | | | | |  |
| 1.4L | Would you say that having a companion / support person in labor will decrease the chance that you will recommend this facility to friends and family, not change the chance of recommending the facility, or increase the chance that you will recommend the facility? | DECREASE CHANCE OF RECOMMENDING FACILITY………...1  NOT CHANGE CHANCE OF RECOMMENDING FACILITY….…2  INCREASE CHANCE OF RECOMMENDING FACILITY……….…3  DON’T KNOW………………………………………………………………….8 | | | | | | | 1.8 |
| 1.4M | Were you given the option to have a companion / support person with you during labor? | NO……………………………………………………………………………..…..0  YES………………………………………………………………………………….1 DON’T KNOW / REMEMBER………………….………………..………8 | | | | | | |  |
| 1.5 | Would you have liked to have a companion / support person with you during labor? | NO……………………………………………………………………………..…..0  YES………………………………………………………………………………….1 DON’T KNOW / REMEMBER………………….………………..………8 | | | | | | | 1.6B  1.8 |
| 1.5A | Why did you not have a companion / support person with you during labor?  ***DO NOT READ LIST***  ***MULTIPLE RESPONSES ALLOWED*** |  | | | | **NO** | | **YES** |  |
|  |  | A. MY COMPANION FROM HOME WAS NOT AVAILABLE | | | | 0 | | 1 |  |
|  |  | B. MY COMPANION FROM HOME DID NOT HAVE THE REQUIRED ID BADGE | | | | 0 | | 1 |  |
|  |  | C. THE COMPANION AT THE FACILITY (WITH GREY UNIFORM) WAS ALREADY HELPING ANOTHER WOMAN | | | | 0 | | 1 |  |
|  |  | D. THE PROVIDER DID NOT ALLOW IT | | | | 0 | | 1 |  |
|  |  | E. THE FACILITY DID NOT ALLOW IT | | | | 0 | | 1 |  |
|  |  | F. I DID NOT KNOW IT WAS AN OPTION | | | | 0 | | 1 |  |
|  |  | G. OTHER (SPECIFY) __________________ | | | | 0 | | 1 |  |
|  |  | H. DON’T KNOW | | | | 0 | | 1 |  |
| 1.6 | Who would you have liked to have with you as a companion / support person during labor?  ***DO NOT READ LIST***  ***MULTIPLE RESPONSES ALLOWED*** |  | | | | **NO** | | **YES** |  |
|  |  | A. HUSBAND OR PARTNER | | | | 0 | | 1 |  |
|  |  | B. MOTHER | | | | 0 | | 1 |  |
|  |  | C. SISTER | | | | 0 | | 1 |  |
|  |  | D. MOTHER-IN-LAW | | | | 0 | | 1 |  |
|  |  | E. TRADITIONAL BIRTH ATTENDANT | | | | 0 | | 1 |  |
|  |  | F. NEIGHBOR | | | | 0 | | 1 |  |
|  |  | G. FRIEND | | | | 0 | | 1 |  |
|  |  | H. OTHER (SPECIFY) __________________ | | | | 0 | | 1 |  |
|  |  | I. A COMPANION PROVIDED BY THE FACILITY | | | | 0 | | 1 |  |
| 1.6A | Why would you have preferred to have [INSERT NAME OF PREFERRED COMPANION(S)] as a companion / support person during labor?  ***DO NOT READ LIST***  ***MULTIPLE RESPONSES ALLOWED*** |  | | | | **NO** | | **YES** | 1.8 |
|  |  | A. KNOWS ME WELL | | | | 0 | | 1 |  |
|  |  | B. EXPERIENCED WITH LABOR/DELIVERY | | | | 0 | | 1 |  |
|  |  | C. I AM NOT SHY AROUND HER/HIM | | | | 0 | | 1 |  |
|  |  | D. WILL GIVE ME ADVICE | | | | 0 | | 1 |  |
|  |  | E. WILL TREAT ME KINDLY | | | | 0 | | 1 |  |
|  |  | F. I TRUST HER/HIM | | | | 0 | | 1 |  |
|  |  | G. SHE/HE WILL NOT GOSSIP ABOUT ME | | | | 0 | | 1 |  |
|  |  | H. OTHER (SPECIFY) __________________ | | | | 0 | | 1 |  |
|  |  | I. DON’T KNOW | | | | 0 | | 1 |  |
| 1.6B | Why did you not want to have a companion / support person during labor?  ***DO NOT READ LIST***  ***MULTIPLE RESPONSES ALLOWED*** |  | | | | **NO** | | **YES** |  |
|  |  | A. I AM EXPERIENCED WITH LABOR | | | | 0 | | 1 |  |
|  |  | B. COMPANION MAY GOSSIP ABOUT ME | | | | 0 | | 1 |  |
|  |  | C. I AM SHY AROUND A COMPANION | | | | 0 | | 1 |  |
|  |  | D. I DO NOT TRUST A COMPANION | | | | 0 | | 1 |  |
|  |  | E. COMPANION MAY TREAT ME BADLY | | | | 0 | | 1 |  |
|  |  | F. OTHER (SPECIFY) __________________ | | | | 0 | | 1 |  |
|  |  | G. DON’T KNOW | | | | 0 | | 1 |  |
| **DELIVERY COMPANION SUB-SECTION:**  Now I am going to ask you about having a companion / support person with you at the time of delivery. A birth companion or support person may be someone you know and brought from the community who may or may not have received a birth companionship orientation and birth companionship ID badge, or it may be a woman wearing a grey uniform who was based at the facility and was by your side throughout labor. A facility staff such as a nurse or midwife is not considered a birth companion. | | | | | | | | | |
| 1.8 | Did you have a companion / support person in the room with you during delivery? By delivery, I mean the moment when the baby was born (either vaginally or by cesarean section). | NO……………………………………………………………………………..…..0  YES………………………………………………………………………………….1 DON’T KNOW / REMEMBER………………….………………………..8 | | | | | | | 1.9M  1.9M |
| 1.9 | Who was your companion / support person(s) during delivery?  ***DO NOT READ LIST***  ***MULTIPLE RESPONSES ALLOWED***  ***IF CLIENT SAYS A HEALTHCARE PROVIDER WAS HER COMPANION, CLARIFY IF IT WAS A COMPANION IN GRAY UNIFORM. GO BACK TO 1.8 AND UPDATE AS NEEDED.*** |  | | | | **NO** | | **YES** |  |
|  |  | A. HUSBAND OR PARTNER | | | | 0 | | 1 |  |
|  |  | B. MOTHER | | | | 0 | | 1 |  |
|  |  | C. SISTER | | | | 0 | | 1 |  |
|  |  | D. MOTHER-IN-LAW | | | | 0 | | 1 |  |
|  |  | E. TRADITIONAL BIRTH ATTENDANT | | | | 0 | | 1 |  |
|  |  | F. NEIGHBOR | | | | 0 | | 1 |  |
|  |  | G. FRIEND | | | | 0 | | 1 |  |
|  |  | H. OTHER (SPECIFY) ____________________ | | | | 0 | | 1 |  |
|  |  | I. A COMPANION PROVIDED BY THE FACILITY (WITH THE GREY UNIFORM) | | | | 0 | | 1 |  |
| 1.9A | Who made the decision about who your companion / support person during delivery would be?  ***DO NOT READ LIST***  ***MULTIPLE RESPONSES ALLOWED*** |  | | | | **NO** | | **YES** |  |
|  |  | A. CLIENT HERSELF | | | | 0 | | 1 |  |
|  |  | B. HUSBAND OR PARTNER | | | | 0 | | 1 |  |
|  |  | C. MOTHER | | | | 0 | | 1 |  |
|  |  | D. MOTHER-IN-LAW | | | | 0 | | 1 |  |
|  |  | E. SOMEONE ELSE IN OWN FAMILY (FATHER, SISTER, BROTHER, ETC) | | | | 0 | | 1 |  |
|  |  | F. SOMEONE ELSE IN HUSBAND’S FAMILY (FATHER-IN-LAW, SISTER-IN-LAW, ETC) | | | | 0 | | 1 |  |
|  |  | G. TRADITIONAL BIRTH ATTENDANT | | | | 0 | | 1 |  |
|  |  | H. VILLAGE LEADER | | | | 0 | | 1 |  |
|  |  | I. HEALTH PROVIDER AT FACILITY | | | | 0 | | 1 |  |
|  |  | J. OTHER (SPECIFY) ___________________ | | | | 0 | | 1 |  |
|  |  | K. DON’T KNOW | | | | 0 | | 1 |  |
| 1.9B | Was [INSERT NAME OF COMPANION(S)] the individual(s) who you preferred to have with you as a companion / support personduring delivery? | NO……………………………………………………………………………..…..0  YES………………………………………………………………………………….1 DON’T KNOW / REMEMBER………………….………………..………8 | | | | | | | 1.9D  1.9D |
| 1.9C | Why did you prefer to have [INSERT NAME OF PREFERRED COMPANION(S)] as a companion / support person during delivery?  ***DO NOT READ LIST***  ***MULTIPLE RESPONSES ALLOWED*** |  | | | | **NO** | | **YES** | 1.9F |
|  |  | A. KNOWS ME WELL | | | | 0 | | 1 |  |
|  |  | B. EXPERIENCED WITH LABOR/DELIVERY | | | | 0 | | 1 |  |
|  |  | C. I AM NOT SHY AROUND HER/HIM | | | | 0 | | 1 |  |
|  |  | D. WILL GIVE ME ADVICE | | | | 0 | | 1 |  |
|  |  | E. WILL TREAT ME KINDLY | | | | 0 | | 1 |  |
|  |  | F. I TRUST HER/HIM | | | | 0 | | 1 |  |
|  |  | G. SHE/HE WILL NOT GOSSIP ABOUT ME | | | | 0 | | 1 |  |
|  |  | H. OTHER (SPECIFY) __________________ | | | | 0 | | 1 |  |
|  |  | I. DON’T KNOW | | | | 0 | | 1 |  |
| 1.9D | Who would you have preferred to have as your companion / support person(s) during delivery?  ***DO NOT READ LIST***  ***MULTIPLE RESPONSES ALLOWED*** |  | | | | **NO** | | **YES** |  |
|  |  | A. HUSBAND OR PARTNER | | | | 0 | | 1 |  |
|  |  | B. MOTHER | | | | 0 | | 1 |  |
|  |  | C. SISTER | | | | 0 | | 1 |  |
|  |  | D. MOTHER-IN-LAW | | | | 0 | | 1 |  |
|  |  | E. TRADITIONAL BIRTH ATTENDANT | | | | 0 | | 1 |  |
|  |  | F. NEIGHBOR | | | | 0 | | 1 |  |
|  |  | G. FRIEND | | | | 0 | | 1 |  |
|  |  | H. OTHER (SPECIFY) ____________________ | | | | 0 | | 1 |  |
|  |  | I. A COMPANION PROVIDED BY THE FACILITY | | | | 0 | | 1 |  |
|  |  | J. DON’T KNOW | | | | 0 | | 1 | 1.9F |
| 1.9E | Why would you have preferred to have [INSERT NAME OF PREFERRED COMPANION(S)] as a companion / support person during delivery?  ***DO NOT READ LIST***  ***MULTIPLE RESPONSES ALLOWED*** |  | | | | **NO** | | **YES** |  |
|  |  | A. KNOWS ME WELL | | | | 0 | | 1 |  |
|  |  | B. EXPERIENCED WITH LABOR/DELIVERY | | | | 0 | | 1 |  |
|  |  | C. I AM NOT SHY AROUND HER/HIM | | | | 0 | | 1 |  |
|  |  | D. WILL GIVE ME ADVICE | | | | 0 | | 1 |  |
|  |  | E. WILL TREAT ME KINDLY | | | | 0 | | 1 |  |
|  |  | F. I TRUST HER/HIM | | | | 0 | | 1 |  |
|  |  | G. SHE/HE WILL NOT GOSSIP ABOUT ME | | | | 0 | | 1 |  |
|  |  | H. OTHER (SPECIFY) __________________ | | | | 0 | | 1 |  |
|  |  | I. DON’T KNOW | | | | 0 | | 1 |  |
| 1.9F | How did you find out that this facility allows a companion / support person during delivery?  **DO NOT READ LIST**  **MULTIPLE RESPONSES ALLOWED** |  | | | | **NO** | | **YES** |  |
|  |  | A. UPON ARRIVAL AT THE FACILITY FOR DELIVERY CARE | | | | 0 | | 1 |  |
|  |  | B. DURING ANTENATAL CARE AT FACILITY | | | | 0 | | 1 |  |
|  |  | C. COMMUNITY HEALTH WORKER | | | | 0 | | 1 |  |
|  |  | D. FRIEND OR FAMILY MEMBER | | | | 0 | | 1 |  |
|  |  | E. RADIO ADVERTISEMENT | | | | 0 | | 1 |  |
|  |  | F. PRINT MATERIALS (FLYERS, POSTERS, BILLBOARD) | | | | 0 | | 1 |  |
|  |  | G. RELIGIOUS GATHERING | | | | 0 | | 1 |  |
|  |  | H. WOMEN’S ASSOCIATION MEETING | | | | 0 | | 1 |  |
|  |  | I. OTHER COMMUNITY MEETING | | | | 0 | | 1 |  |
|  |  | J. OTHER (SPECIFY) ___________________ | | | | 0 | | 1 |  |
|  |  | K. DON’T KNOW | | | | 0 | | 1 |  |
| 1.9G | What did your companion / support person do to support you during delivery?  **DO NOT READ LIST**  **MULTIPLE RESPONSES ALLOWED**  **PROBE:** Anything else? |  | | | | **NO** | | **YES** |  |
|  |  | A. GAVE ME ADVICE OR INSTRUCTIONS | | | | 0 | | 1 |  |
|  |  | B. HELPED ME TO WALK AROUND | | | | 0 | | 1 |  |
|  |  | C. HELPED ME TO CHANGE POSITION | | | | 0 | | 1 |  |
|  |  | D. GAVE ME FOOD TO EAT | | | | 0 | | 1 |  |
|  |  | E. GAVE ME FLUIDS TO DRINK | | | | 0 | | 1 |  |
|  |  | F. RUBBED MY BACK | | | | 0 | | 1 |  |
|  |  | G. CLEANED MY CLOTHES OR LINENS | | | | 0 | | 1 |  |
|  |  | H. STAYED WITH ME BY MY SIDE FOR THE MAJORITY OF THE TIME | | | | 0 | | 1 |  |
|  |  | I. COMFORTED ME WITH KIND WORDS, SINGING, PRAYER, ETC | | | | 0 | | 1 |  |
|  |  | J. HELPED ME TRY THINGS TO REDUCE THE PAIN (NON-MEDICAL) | | | | 0 | | 1 |  |
|  |  | K. HELPED THE STAFF | | | | 0 | | 1 |  |
|  |  | L. COMMUNICATED WITH THE STAFF | | | | 0 | | 1 |  |
|  |  | M. COMMUNICATED WITH MY FAMILY/ HUSBAND | | | | 0 | | 1 |  |
|  |  | N. HELPED ENSURE PRIVACY | | | | 0 | | 1 |  |
|  |  | O. OTHER (SPECIFY) ___________________ | | | | 0 | | 1 |  |
|  |  | P. NOTHING | | | | 0 | | 1 |  |
|  |  | Q. DON’T KNOW | | | | 0 | | 1 |  |
| 1.9H | What would you have liked your companion / support person to do for you during delivery that she did not do, if anything?  **DO NOT READ LIST**  **MULTIPLE RESPONSES ALLOWED**  **PROBE:** Anything else? |  | | | | **NO** | | **YES** |  |
|  |  | A. GIVE ME ADVICE OR INSTRUCTIONS | | | | 0 | | 1 |  |
|  |  | B. HELP ME TO WALK AROUND | | | | 0 | | 1 |  |
|  |  | C. HELP ME TO CHANGE POSITION | | | | 0 | | 1 |  |
|  |  | D. GIVE ME FOOD TO EAT | | | | 0 | | 1 |  |
|  |  | E. GIVE ME FLUIDS TO DRINK | | | | 0 | | 1 |  |
|  |  | F. RUB MY BACK | | | | 0 | | 1 |  |
|  |  | G. CLEAN MY CLOTHES OR LINENS | | | | 0 | | 1 |  |
|  |  | H. STAY WITH ME BY MY SIDE FOR THE MAJORITY OF THE TIME | | | | 0 | | 1 |  |
|  |  | I. COMFORTED ME WITH KIND WORDS, SINGING, PRAYER, ETC | | | | 0 | | 1 |  |
|  |  | J. HELP ME TRY THINGS TO REDUCE THE PAIN (NON-MEDICAL) | | | | 0 | | 1 |  |
|  |  | K. HELP THE STAFF | | | | 0 | | 1 |  |
|  |  | L. COMMUNICATE WITH THE STAFF | | | | 0 | | 1 |  |
|  |  | M. COMMUNICATE WITH MY FAMILY/ HUSBAND | | | | 0 | | 1 |  |
|  |  | N. HELP ENSURE PRIVACY | | | | 0 | | 1 |  |
|  |  | O. OTHER (SPECIFY) ___________________ | | | | 0 | | 1 |  |
|  |  | P. NOTHING | | | | 0 | | 1 |  |
|  |  | Q. DON’T KNOW | | | | 0 | | 1 |  |
| 1.9I | A. Overall, would you say that having a companion / support person during delivery was satisfying or dissatisfying? | DISATISFYING….………………………..……………………………..…….1  SATISFYING…….…………………………..……………………..…………..2  NEITHER DISSATISFYING NOR SATISFYING……..……….………3  DON’T KNOW………………………….…………….………………………..8 | | | | | | | 1.9J  1.9J |
|  | B. Would you say it was very […] or a little […]?   **USE ANSWER FROM 1.9I_A IN PLACE OF […] (SATISFIED OR DISSATISFIED)** | VERY DISSATISFYING……………………….………………….………….1  A LITTLE DISSATISFYING….…………………………..……....………..2  A LITTLE SATISFYING…….…………………………………….….………4  VERY SATISFYING…….………………………………………………………5  DON’T KNOW………………………………………………………………….8 | | | | | | |  |
| 1.9J | Would you say that having a companion / support person during delivery made your delivery experience worse, did not change your delivery experience, or made your delivery experience better? | MADE DELIVERY EXPERIENCE WORSE……………………………..1  DID NOT CHANGE DELIVERY EXPERIENCE…………..……………2  MADE DELIVERY EXPERIENCE BETTER………………..……………3  DON’T KNOW………………………………………………….………………8 | | | | | | |  |
| 1.9K | Would you say that having a companion / support person during delivery will decrease the chance that you will return to this facility in the future, not change the chance of returning to the facility, or increase the chance that you will return to the facility? | DECREASE CHANCE OF RETURNING……………….……..………..1  NOT CHANGE CHANCE OF RETURNING……………………………2  INCREASE CHANCE OF RETURNING……………….………..………3  DON’T KNOW……………………………………………….………….…….8 | | | | | | |  |
| 1.9L | Would you say that having a companion / support person during delivery will decrease the chance that you will recommend this facility to friends and family, not change the chance of recommending the facility, or increase the chance that you will recommend the facility? | DECREASE CHANCE OF RECOMMENDING FACILITY………...1  NOT CHANGE CHANCE OF RECOMMENDING FACILITY….…2  INCREASE CHANCE OF RECOMMENDING FACILITY……….…3  DON’T KNOW………………………………………………………………….8 | | | | | | | 1.12A |
| 1.9M | Were you given the option to have a companion / support person with you during delivery? | NO……………………………………………………………………………..…..0  YES………………………………………………………………………………….1 DON’T KNOW / REMEMBER………………….………………..………8 | | | | | | |  |
| 1.10 | Would you have liked to have a companion / support person with you during delivery? | NO……………………………………………………………………………..…..0  YES………………………………………………………………………………….1 DON’T KNOW / REMEMBER………………….………………..………8 | | | | | | | 1.11B  1.12A |
| 1.10A | Why did you not have a companion / support person with you during delivery?  ***DO NOT READ LIST***  ***MULTIPLE RESPONSES ALLOWED*** |  | | | | **NO** | | **YES** |  |
|  |  | A. MY COMPANION FROM HOME WAS NOT AVAILABLE | | | | 0 | | 1 |  |
|  |  | B. MY COMPANION FROM HOME DID NOT HAVE THE REQUIRED ID BADGE | | | | 0 | | 1 |  |
|  |  | C. THE COMPANION AT THE FACILITY (WITH GREY UNIFORM) WAS ALREADY HELPING ANOTHER WOMAN | | | | 0 | | 1 |  |
|  |  | D. THE PROVIDER DID NOT ALLOW IT | | | | 0 | | 1 |  |
|  |  | E. THE FACILITY DID NOT ALLOW IT | | | | 0 | | 1 |  |
|  |  | F. I DID NOT KNOW IT WAS AN OPTION | | | | 0 | | 1 |  |
|  |  | G. OTHER (SPECIFY) __________________ | | | | 0 | | 1 |  |
|  |  | H. DON’T KNOW | | | | 0 | | 1 |  |
| 1.11 | Who would you have liked to have with you as a companion / support person during delivery?  ***DO NOT READ LIST***  ***MULTIPLE RESPONSES ALLOWED*** |  | | | | **NO** | | **YES** |  |
|  |  | A. HUSBAND OR PARTNER | | | | 0 | | 1 |  |
|  |  | B. MOTHER | | | | 0 | | 1 |  |
|  |  | C. SISTER | | | | 0 | | 1 |  |
|  |  | D. MOTHER-IN-LAW | | | | 0 | | 1 |  |
|  |  | E. TRADITIONAL BIRTH ATTENDANT | | | | 0 | | 1 |  |
|  |  | F. NEIGHBOR | | | | 0 | | 1 |  |
|  |  | G. FRIEND | | | | 0 | | 1 |  |
|  |  | H. OTHER (SPECIFY) __________________ | | | | 0 | | 1 |  |
|  |  | I. A COMPANION PROVIDED BY THE FACILITY | | | | 0 | | 1 |  |
| 1.11A | Why would you have preferred to have [INSERT NAME OF PREFERRED COMPANION(S)] as a companion / support person during delivery?  ***DO NOT READ LIST***  ***MULTIPLE RESPONSES ALLOWED*** |  | | | | **NO** | | **YES** | 1.12A |
|  |  | A. KNOWS ME WELL | | | | 0 | | 1 |  |
|  |  | B. EXPERIENCED WITH LABOR/DELIVERY | | | | 0 | | 1 |  |
|  |  | C. I AM NOT SHY AROUND HER/HIM | | | | 0 | | 1 |  |
|  |  | D. WILL GIVE ME ADVICE | | | | 0 | | 1 |  |
|  |  | E. WILL TREAT ME KINDLY | | | | 0 | | 1 |  |
|  |  | F. I TRUST HER/HIM | | | | 0 | | 1 |  |
|  |  | G. SHE/HE WILL NOT GOSSIP ABOUT ME | | | | 0 | | 1 |  |
|  |  | H. OTHER (SPECIFY) __________________ | | | | 0 | | 1 |  |
|  |  | I. DON’T KNOW | | | | 0 | | 1 |  |
| 1.11B | Why did you not want to have a companion / support person during delivery?  ***DO NOT READ LIST***  ***MULTIPLE RESPONSES ALLOWED*** |  | | | | **NO** | | **YES** |  |
|  |  | A. I AM EXPERIENCED WITH DELIVERY | | | | 0 | | 1 |  |
|  |  | B. COMPANION MAY GOSSIP ABOUT ME | | | | 0 | | 1 |  |
|  |  | C. I AM SHY AROUND A COMPANION | | | | 0 | | 1 |  |
|  |  | D. I DO NOT TRUST A COMPANION | | | | 0 | | 1 |  |
|  |  | E. COMPANION MAY TREAT ME BADLY | | | | 0 | | 1 |  |
|  |  | F. OTHER (SPECIFY) __________________ | | | | 0 | | 1 |  |
|  |  | G. DON’T KNOW | | | | 0 | | 1 |  |
| **POSTPARTUM COMPANION SUB-SECTION:**  Now I am going to ask you about having a companion or support person with you after the baby was born. We call this the postpartum period. A birth companion or support person may be someone you know and brought from the community who may or may not have received a birth companionship orientation and birth companionship ID badge, or it may be a woman wearing a grey uniform who was based at the facility and was by your side. A facility staff such as a nurse or midwife is not considered a companion. | | | | | | | | | |
| 1.12A | Did you have a companion / support person in the room with you in the postpartum period? By postpartum, I mean in the time after the baby was born. | NO……………………………………………………………………………..…..0  YES………………………………………………………………………………….1 DON’T KNOW / REMEMBER………………….………………………..8 | | | | | | | 1.12O  1.12O |
| 1.12B | Who was your companion / support person(s) after the baby was born?  ***DO NOT READ LIST***  ***MULTIPLE RESPONSES ALLOWED***  ***IF CLIENT SAYS A HEALTHCARE PROVIDER WAS HER COMPANION, CLARIFY IF IT WAS A COMPANION IN GRAY UNIFORM. GO BACK TO 1.12A AND UPDATE AS NEEDED.*** |  | | | | **NO** | | **YES** |  |
|  |  | A. HUSBAND OR PARTNER | | | | 0 | | 1 |  |
|  |  | B. MOTHER | | | | 0 | | 1 |  |
|  |  | C. SISTER | | | | 0 | | 1 |  |
|  |  | D. MOTHER-IN-LAW | | | | 0 | | 1 |  |
|  |  | E. TRADITIONAL BIRTH ATTENDANT | | | | 0 | | 1 |  |
|  |  | F. NEIGHBOR | | | | 0 | | 1 |  |
|  |  | G. FRIEND | | | | 0 | | 1 |  |
|  |  | H. OTHER (SPECIFY) ____________________ | | | | 0 | | 1 |  |
|  |  | I. A COMPANION PROVIDED BY THE FACILITY (WITH THE GREY UNIFORM) | | | | 0 | | 1 |  |
| 1.12C | Who made the decision about who your companion / support person after the baby was born would be?  ***DO NOT READ LIST***  ***MULTIPLE RESPONSES ALLOWED*** |  | | | | **NO** | | **YES** |  |
|  |  | A. CLIENT HERSELF | | | | 0 | | 1 |  |
|  |  | B. HUSBAND OR PARTNER | | | | 0 | | 1 |  |
|  |  | C. MOTHER | | | | 0 | | 1 |  |
|  |  | D. MOTHER-IN-LAW | | | | 0 | | 1 |  |
|  |  | E. SOMEONE ELSE IN OWN FAMILY (FATHER, SISTER, BROTHER, ETC) | | | | 0 | | 1 |  |
|  |  | F. SOMEONE ELSE IN HUSBAND’S FAMILY (FATHER-IN-LAW, SISTER-IN-LAW, ETC) | | | | 0 | | 1 |  |
|  |  | G. TRADITIONAL BIRTH ATTENDANT | | | | 0 | | 1 |  |
|  |  | H. VILLAGE LEADER | | | | 0 | | 1 |  |
|  |  | I. HEALTH PROVDER AT FACILITY….……….…… | | | | 0 | | 1 |  |
|  |  | J. OTHER (SPECIFY) ___________________ | | | | 0 | | 1 |  |
|  |  | K. DON’T KNOW | | | | 0 | | 1 |  |
| 1.12D | Was [INSERT NAME OF COMPANION(S)] the individual(s) who you preferred to have with you as a companion / support person after the baby was born? | NO……………………………………………………………………………..…..0  YES………………………………………………………………………………….1 DON’T KNOW / REMEMBER………………….………………..………8 | | | | | | | 1.12F  1.12F |
| 1.12E | Why did you prefer to have [INSERT NAME OF PREFERRED COMPANION(S)] as a companion / support person after the baby was born?  ***DO NOT READ LIST***  ***MULTIPLE RESPONSES ALLOWED*** |  | | | | **NO** | | **YES** | 1.12H |
|  |  | A. KNOWS ME WELL | | | | 0 | | 1 |  |
|  |  | B. EXPERIENCED WITH LABOR/DELIVERY | | | | 0 | | 1 |  |
|  |  | C. I AM NOT SHY AROUND HER/HIM | | | | 0 | | 1 |  |
|  |  | D. WILL GIVE ME ADVICE | | | | 0 | | 1 |  |
|  |  | E. WILL TREAT ME KINDLY | | | | 0 | | 1 |  |
|  |  | F. I TRUST HER/HIM | | | | 0 | | 1 |  |
|  |  | G. SHE/HE WILL NOT GOSSIP ABOUT ME | | | | 0 | | 1 |  |
|  |  | H. OTHER (SPECIFY) __________________ | | | | 0 | | 1 |  |
|  |  | I. DON’T KNOW | | | | 0 | | 1 |  |
| 1.12F | Who would you have preferred to have as your companion / support person(s) after the baby was born?  ***DO NOT READ LIST***  ***MULTIPLE RESPONSES ALLOWED*** |  | | | | **NO** | | **YES** |  |
|  |  | A. HUSBAND OR PARTNER | | | | 0 | | 1 |  |
|  |  | B. MOTHER | | | | 0 | | 1 |  |
|  |  | C. SISTER | | | | 0 | | 1 |  |
|  |  | D. MOTHER-IN-LAW | | | | 0 | | 1 |  |
|  |  | E. TRADITIONAL BIRTH ATTENDANT | | | | 0 | | 1 |  |
|  |  | F. NEIGHBOR | | | | 0 | | 1 |  |
|  |  | G. FRIEND | | | | 0 | | 1 |  |
|  |  | H. OTHER (SPECIFY) ____________________ | | | | 0 | | 1 |  |
|  |  | I. A COMPANION PROVIDED BY THE FACILITY | | | | 0 | | 1 |  |
|  |  | J. DON’T KNOW | | | | 0 | | 1 | 1.12H |
| 1.12G | Why would you have preferred to have [INSERT NAME OF PREFERRED COMPANION(S)] as a companion / support person after the baby was born?  ***DO NOT READ LIST***  ***MULTIPLE RESPONSES ALLOWED*** |  | | | | **NO** | | **YES** |  |
|  |  | A. KNOWS ME WELL | | | | 0 | | 1 |  |
|  |  | B. EXPERIENCED WITH POSTPARTUM | | | | 0 | | 1 |  |
|  |  | C. I AM NOT SHY AROUND HER/HIM | | | | 0 | | 1 |  |
|  |  | D. WILL GIVE ME ADVICE | | | | 0 | | 1 |  |
|  |  | E. WILL TREAT ME KINDLY | | | | 0 | | 1 |  |
|  |  | F. I TRUST HER/HIM | | | | 0 | | 1 |  |
|  |  | G. SHE/HE WILL NOT GOSSIP ABOUT ME | | | | 0 | | 1 |  |
|  |  | H. OTHER (SPECIFY) __________________ | | | | 0 | | 1 |  |
|  |  | I. DON’T KNOW | | | | 0 | | 1 |  |
| 1.12H | How did you find out that this facility allows a companion / support person after the baby is born?  **DO NOT READ LIST**  **MULTIPLE RESPONSES ALLOWED** |  | | | | **NO** | | **YES** |  |
|  |  | A. UPON ARRIVAL AT THE FACILITY FOR DELIVERY CARE | | | | 0 | | 1 |  |
|  |  | B. DURING ANTENATAL CARE AT FACILITY | | | | 0 | | 1 |  |
|  |  | C. COMMUNITY HEALTH WORKER | | | | 0 | | 1 |  |
|  |  | D. FRIEND OR FAMILY MEMBER | | | | 0 | | 1 |  |
|  |  | E. RADIO ADVERTISEMENT | | | | 0 | | 1 |  |
|  |  | F. PRINT MATERIALS (FLYERS, POSTERS, BILLBOARD) | | | | 0 | | 1 |  |
|  |  | G. RELIGIOUS GATHERING | | | | 0 | | 1 |  |
|  |  | H. WOMEN’S ASSOCIATION MEETING | | | | 0 | | 1 |  |
|  |  | I. OTHER COMMUNITY MEETING | | | | 0 | | 1 |  |
|  |  | J. OTHER (SPECIFY) ___________________ | | | | 0 | | 1 |  |
|  |  | K. DON’T KNOW | | | | 0 | | 1 |  |
| 1.12I | What did your companion / support person do to support you after the baby was born?  **DO NOT READ LIST**  **MULTIPLE RESPONSES ALLOWED**  **PROBE:** Anything else? |  | | | | **NO** | | **YES** |  |
|  |  | A. GAVE ME ADVICE OR INSTRUCTIONS | | | | 0 | | 1 |  |
|  |  | B. HELPED ME TO WALK AROUND | | | | 0 | | 1 |  |
|  |  | C. HELPED ME TO CHANGE POSITION | | | | 0 | | 1 |  |
|  |  | D. GAVE ME FOOD TO EAT | | | | 0 | | 1 |  |
|  |  | E. GAVE ME FLUIDS TO DRINK | | | | 0 | | 1 |  |
|  |  | F. RUBBED MY BACK | | | | 0 | | 1 |  |
|  |  | G. CLEANED MY CLOTHES OR LINENS | | | | 0 | | 1 |  |
|  |  | H. STAYED WITH ME BY MY SIDE FOR THE MAJORITY OF THE TIME | | | | 0 | | 1 |  |
|  |  | I. COMFORTED ME WITH KIND WORDS, SINGING, PRAYER, ETC | | | | 0 | | 1 |  |
|  |  | J. HELPED ME TRY THINGS TO REDUCE THE PAIN (NON-MEDICAL) | | | | 0 | | 1 |  |
|  |  | K. HELPED THE STAFF | | | | 0 | | 1 |  |
|  |  | L. COMMUNICATED WITH THE STAFF | | | |  | |  |  |
|  |  | M. COMMUNICATED WITH MY FAMILY/ HUSBAND | | | | 0 | | 1 |  |
|  |  | N. HELPED ENSURE PRIVACY | | | | 0 | | 1 |  |
|  |  | O. TALKED WITH ME ABOUT FAMILY PLANNING | | | | 0 | | 1 |  |
|  |  | P. HELPED CARE FOR THE BABY | | | | 0 | | 1 |  |
|  |  | Q. SUPPORTED BREASTFEEDING | | | | 0 | | 1 |  |
|  |  | R. OTHER (SPECIFY) ___________________ | | | | 0 | | 1 |  |
|  |  | S. NOTHING | | | | 0 | | 1 |  |
|  |  | T. DON’T KNOW | | | | 0 | | 1 |  |
| 1.12J | What would you have liked your companion / support person to do for you after the baby was born that she did not do, if anything?  **DO NOT READ LIST**  **MULTIPLE RESPONSES ALLOWED**  **PROBE:** Anything else? |  | | | | **NO** | | **YES** |  |
|  |  | A. GIVE ME ADVICE OR INSTRUCTIONS | | | | 0 | | 1 |  |
|  |  | B. HELP ME TO WALK AROUND | | | | 0 | | 1 |  |
|  |  | C. HELP ME TO CHANGE POSITION | | | | 0 | | 1 |  |
|  |  | D. GIVE ME FOOD TO EAT | | | | 0 | | 1 |  |
|  |  | E. GIVE ME FLUIDS TO DRINK | | | | 0 | | 1 |  |
|  |  | F. RUB MY BACK | | | | 0 | | 1 |  |
|  |  | G. CLEAN MY CLOTHES OR LINENS | | | | 0 | | 1 |  |
|  |  | H. STAY WITH ME BY MY SIDE FOR THE MAJORITY OF THE TIME | | | | 0 | | 1 |  |
|  |  | I. COMFORTED ME WITH KIND WORDS, SINGING, PRAYER, ETC | | | | 0 | | 1 |  |
|  |  | J. HELP ME TRY THINGS TO REDUCE THE PAIN (NON-MEDICAL) | | | | 0 | | 1 |  |
|  |  | K. HELP THE STAFF | | | | 0 | | 1 |  |
|  |  | L. COMMUNICATE WITH THE STAFF | | | | 0 | | 1 |  |
|  |  | M. COMMUNICATE WITH MY FAMILY/ HUSBAND | | | | 0 | | 1 |  |
|  |  | N. HELP ENSURE PRIVACY | | | | 0 | | 1 |  |
|  |  | O. TALK WITH ME ABOUT FAMILY PLANNING | | | | 0 | | 1 |  |
|  |  | P. HELP CARE FOR THE BABY | | | | 0 | | 1 |  |
|  |  | Q. SUPPORT BREASTFEEDING | | | | 0 | | 1 |  |
|  |  | R. OTHER (SPECIFY) ___________________ | | | | 0 | | 1 |  |
|  |  | S. NOTHING | | | | 0 | | 1 |  |
|  |  | T. DON’T KNOW | | | | 0 | | 1 |  |
| 1.12K | A. Overall, would you say that having a companion / support person after the baby was born was satisfying or dissatisfying? | DISSATISFYING….………………………..…………………………….…….1  SATISFYING…….…………………………..……………………..…………...2  NEITHER DISSATISFYING NOR SATISFYING……..……….………3  DON’T KNOW………………………….…………….………………………..8 | | | | | | | 1.12L  1.12L |
|  | B. Would you say it was very […] or a little […]?   **USE ANSWER FROM 1.12K_A IN PLACE OF […] (SATISFIED OR DISSATISFIED)** | VERY DISSATISFYING……………………….………………….……….….1  A LITTLE DISSATISFYING….…………………………..……...……..…..2  A LITTLE SATISFYING…….………………………………………….………4  VERY SATISFYING…….………………………………………….……..……5  DON’T KNOW………………………………………………………………….8 | | | | | | |  |
| 1.12L | Would you say that having a companion / support person after the baby was born made your postpartum experience worse, did not change your postpartum experience, or made your postpartum experience better? | MADE POSTPARTUM EXPERIENCE WORSE……………………..1  DID NOT CHANGE POSTPARTUM EXPERIENCE…………..……2  MADE POSTPARTUM EXPERIENCE BETTER………………..……3  DON’T KNOW………………………………………………………….………8 | | | | | | |  |
| 1.12M | Would you say that having a companion / support person after the baby was born decreases the chance that you will return to this facility in the future, does not change the chance of returning to the facility, or increases the chance that you will return to the facility? | DECREASES CHANCE OF RETURNING……………………….……..1  NOT CHANGE CHANCE OF RETURNING……………………..……2  INCREASES CHANCE OF RETURNING………………………….……3  DON’T KNOW…………………………………………………………..…….8 | | | | | | |  |
| 1.12N | Would you say that having a companion / support person after the baby was born decreases the chance that you will recommend this facility to friends and family, does not change the chance of recommending the facility, or increases the chance that you will recommend the facility? | DECREASES CHANCE OF RECOMMENDING FACILITY……….1  NOT CHANGE CHANCE OF RECOMMENDING FACILITY……2  INCREASES CHANCE OF RECOMMENDING FACILITY……..…3  DON’T KNOW………………………………………………………………….8 | | | | | | | 1.13 |
| 1.12O | Were you given the option to have a companion / support person with you after the baby was born? | NO……………………………………………………………………………..…..0  YES………………………………………………………………………………….1 DON’T KNOW / REMEMBER………………….………………..………8 | | | | | | |  |
| 1.12P | Would you have liked to have a companion / support person with you after the baby was born? | NO……………………………………………………………………………..…..0  YES………………………………………………………………………………….1 DON’T KNOW / REMEMBER………………….………………..………8 | | | | | | | 1.12T  1.13 |
| 1.12Q | Why did you not have a companion / support person with you after the baby was born?  ***DO NOT READ LIST***  ***MULTIPLE RESPONSES ALLOWED*** |  | | | | **NO** | | **YES** |  |
|  |  | A. MY COMPANION FROM HOME WAS NOT AVAILABLE | | | | 0 | | 1 |  |
|  |  | B. MY COMPANION FROM HOME DID NOT HAVE THE REQUIRED ID BADGE | | | | 0 | | 1 |  |
|  |  | C. THE COMPANION AT THE FACILITY (WITH GREY UNIFORM) WAS ALREADY HELPING ANOTHER WOMAN | | | | 0 | | 1 |  |
|  |  | D. THE PROVIDER DID NOT ALLOW IT | | | | 0 | | 1 |  |
|  |  | E. THE FACILITY DID NOT ALLOW IT | | | | 0 | | 1 |  |
|  |  | F. I DID NOT KNOW IT WAS AN OPTION | | | | 0 | | 1 |  |
|  |  | G. OTHER (SPECIFY) __________________ | | | | 0 | | 1 |  |
|  |  | H. DON’T KNOW | | | | 0 | | 1 |  |
| 1.12R | Who would you have liked to have with you as a companion / support person after the baby was born?  ***DO NOT READ LIST***  ***MULTIPLE RESPONSES ALLOWED*** |  | | | | **NO** | | **YES** |  |
|  |  | A. HUSBAND OR PARTNER | | | | 0 | | 1 |  |
|  |  | B. MOTHER | | | | 0 | | 1 |  |
|  |  | C. SISTER | | | | 0 | | 1 |  |
|  |  | D. MOTHER-IN-LAW | | | | 0 | | 1 |  |
|  |  | E. TRADITIONAL BIRTH ATTENDANT | | | | 0 | | 1 |  |
|  |  | F. NEIGHBOR | | | | 0 | | 1 |  |
|  |  | G. FRIEND | | | | 0 | | 1 |  |
|  |  | H. OTHER (SPECIFY) __________________ | | | | 0 | | 1 |  |
|  |  | I. A COMPANION PROVIDED BY THE FACILITY | | | | 0 | | 1 |  |
| 1.12S | Why would you have preferred to have [INSERT NAME OF PREFERRED COMPANION(S)] as a companion / support person after the baby was born?  ***DO NOT READ LIST***  ***MULTIPLE RESPONSES ALLOWED*** |  | | | | **NO** | | **YES** | 1.13 |
|  |  | A. KNOWS ME WELL | | | | 0 | | 1 |  |
|  |  | B. EXPERIENCED WITH POSTPARTUM | | | | 0 | | 1 |  |
|  |  | C. I AM NOT SHY AROUND HER/HIM | | | | 0 | | 1 |  |
|  |  | D. WILL GIVE ME ADVICE | | | | 0 | | 1 |  |
|  |  | E. WILL TREAT ME KINDLY | | | | 0 | | 1 |  |
|  |  | F. I TRUST HER/HIM | | | | 0 | | 1 |  |
|  |  | G. SHE/HE WILL NOT GOSSIP ABOUT ME | | | | 0 | | 1 |  |
|  |  | H. OTHER (SPECIFY) __________________ | | | | 0 | | 1 |  |
|  |  | I. DON’T KNOW | | | | 0 | | 1 |  |
| 1.12T | Why did you not want to have a companion / support person after the baby was born?  ***DO NOT READ LIST***  ***MULTIPLE RESPONSES ALLOWED*** |  | | | | **NO** | | **YES** |  |
|  |  | A. I AM EXPERIENCED WITH POSTPARTUM | | | | 0 | | 1 |  |
|  |  | B. COMPANION MAY GOSSIP ABOUT ME | | | | 0 | | 1 |  |
|  |  | C. I AM SHY AROUND A COMPANION | | | | 0 | | 1 |  |
|  |  | D. I DO NOT TRUST A COMPANION | | | | 0 | | 1 |  |
|  |  | E. COMPANION MAY TREAT ME BADLY | | | | 0 | | 1 |  |
|  |  | F. OTHER (SPECIFY) __________________ | | | | 0 | | 1 |  |
|  |  | G. DON’T KNOW | | | | 0 | | 1 |  |
| 1.13 | **During this visit, did the health care provider…** | | **NO** | **YES** | | **NA** | | **DK** |  |
|  | A. Ask the reason for your visit? | | 0 | 1 | | 6 | | 8 |  |
|  | B. Measure your blood pressure? | | 0 | 1 | | 6 | | 8 |  |
|  | C. Take your temperature? | | 0 | 1 | | 6 | | 8 |  |
|  | D. Perform an abdominal exam? | | 0 | 1 | | 6 | | 8 |  |
|  | E. Perform a vaginal exam? | | 0 | 1 | | 6 | | 8 |  |
|  | F. Listen for the baby’s heart beat during labor? | | 0 | 1 | | 6 | | 8 |  |
|  | G. Counsel you about danger signs you should look for in yourself such as too much bleeding, fever, or breast pain? | | 0 | 1 | | 6 | | 8 |  |
|  | H. Counsel you about danger signs you should look for in your baby such as refusing to breastfeed, fever, or convulsions (fits)? | | 0 | 1 | | 6 | | 8 |  |
|  | I. Tell you what to do if you or the baby have any problems? | | 0 | 1 | | 6 | | 8 |  |
|  | J. Counsel you on good body hygiene to prevent infections? | | 0 | 1 | | 6 | | 8 |  |
|  | K. Counsel you on breastfeeding? | | 0 | 1 | | 6 | | 8 |  |
|  | L. Counsel you about exclusive breastfeeding (not using any other fluid / food except breastmilk)? | | 0 | 1 | | 6 | | 8 |  |
|  | M. Ask about your reproductive goals? | | 0 | 1 | | 6 | | 8 |  |
|  | N. Counsel you on when you can have sex with your husband / partner? | | 0 | 1 | | 6 | | 8 |  |
|  | O. Counsel you on when you can bear another pregnancy? | | 0 | 1 | | 6 | | 8 |  |
|  | P. Counsel you on the risks of sexually transmitted infections (STIs), including HIV? | | 0 | 1 | | 6 | | 8 |  |
|  | Q. Counsel you on how to prevent sexually transmitted infections (STIs), including HIV? | | 0 | 1 | | 6 | | 8 |  |
|  | R. Tell you when to return for a follow-up visit? | | 0 | 1 | | 6 | | 8 |  |
|  | ***IF YES TO Q1.13R, ASK:***   S. When did the health care provider ask you to return for a follow-up visit?   ***DO NOT READ LIST***  ***MULTIPLE RESPONSES ALLOWED*** |  | | | | **NO** | | **YES** |  |
|  |  | a. IN 1-2 DAYS | | | | 0 | | 1 |  |
|  |  | b. IN 1 WEEK | | | | 0 | | 1 |  |
|  |  | c. IN 2 WEEKS | | | | 0 | | 1 |  |
|  |  | d. IN 4 WEEKS | | | | 0 | | 1 |  |
|  |  | e. IN 6 WEEKS | | | | 0 | | 1 |  |
|  |  | f. AFTER 6 WEEKS | | | | 0 | | 1 |  |
|  |  | g. OTHER (SPECIFY) ___________________ | | | | 0 | | 1 |  |
| 1.17 | Did you have any complications during your labor and delivery? | NO……………………………………..……………………………………..…..0  YES…………………………………………..…….………………………….….1  DON’T KNOW………………………………………………………….…….8 | | | | | | | 2.1  2.1 |
| 1.18A | What complication(s) did you have?  ***RECORD IN SPACE PROVIDED***  ***MULTIPE RESPONSES POSSIBLE*** |  | | | | **NO** | | **YES** |  |
|  |  | A. POSTPARTUM HEMORRHAGE (BLEEDING TOO MUCH AFTER DELIVERY) | | | | 0 | | 1 |  |
|  |  | B. PUERPURAL SEPSIS (INFECTION AFTER DELIVERY) | | | | 0 | | 1 |  |
|  |  | C. RETAINED PLACENTA | | | | 0 | | 1 |  |
|  |  | D. OBSTRUCTED/PROLONGED LABOR | | | | 0 | | 1 |  |
|  |  | E. UTERINE RUTURE | | | | 0 | | 1 |  |
|  |  | F. PREMATURE RUPTURE OF MEMBRANES (WATER BREAKING BEFORE LABOR) | | | | 0 | | 1 |  |
|  |  | G. PRE-ECLAMPSIA/ECLAMPSIA (FITS) | | | | 0 | | 1 |  |
|  |  | H. HYPERTENSION (HIGH BLOOD PRESSURE) | | | | 0 | | 1 |  |
|  |  | I. BLEEDING DURING PREGNANCY | | | | 0 | | 1 |  |
|  |  | J. PERINEAL TEAR OR EPISIOTOMY | | | | 0 | | 1 |  |
|  |  | K. OTHER (SPECIFY) ______________ | | | | 0 | | 1 |  |
| **SECTION 2. CLIENT SATISFACTION WITH LABOR AND DELIVERY CARE VISIT** | | | | | | | | | |
| **I would like to ask you some questions about the services you received during this visit. Please refer to the health care provider that gave you the most information during your visit. The provider will not learn of your responses, so please be honest. This information will help improve delivery services. We are asking about the health providers and NOT the companion you had from home or the companions based at the health facility with the grey uniforms.** | | | | | | | | | |
| 2.1 | **When you came to the facility during this visit, did the staff…** | | **NO** | **YES** | | **NA** | | **DK** |  |
|  | A. Encourage you to have a support person with you throughout labor and delivery? | | 0 | 1 | | 6 | | 8 |  |
|  | B. Introduce themselves? | | 0 | 1 | | 6 | | 8 |  |
|  | C. Greet you respectfully? | | 0 | 1 | | 6 | | 8 |  |
|  | E. Ask if you have questions? | | 0 | 1 | | 6 | | 8 |  |
|  | F. Explain to you what will happen during your labor and delivery? | | 0 | 1 | | 6 | | 8 |  |
|  | G. Explain procedures or exams to you before proceeding? | | 0 | 1 | | 6 | | 8 |  |
|  | H. Get your consent before proceeding with procedures or exams? | | 0 | 1 | | 6 | | 8 |  |
|  | I. Inform you of the findings from procedures or exams? | | 0 | 1 | | 6 | | 8 |  |
|  | J. Provide privacy during counseling or exams such as using a private room, screens, curtains, or cloths to cover you? | | 0 | 1 | | 6 | | 8 |  |
|  | ***IF YES TO Q2.1J, PROBE:***   K. What was used to provide privacy?   ***DO NOT READ LIST***  ***MULTIPLE RESPONSES ALLOWED*** |  | | | | **NO** | | **YES** |  |
|  |  | a. PRIVATE ROOM WITH DOOR CLOSED | | | | 0 | | 1 |  |
|  |  | b. WARD SCREEN / CURTAINS AROUND BED | | | | 0 | | 1 |  |
|  |  | c. CLOTHS / DRAPES TO COVER BODY | | | | 0 | | 1 |  |
|  |  | d. OTHER (SPECIFY) ___________________ | | | | 0 | | 1 |  |
|  |  | e. DON’T KNOW / REMEMBER | | | | 0 | | 1 |  |
| 2.2 | When meeting with the health care provider during the visit, do you think other clients could hear what you said? | NO……………………………………………………………………………..…..0  YES………………………………………………………………………………….1 DON’T KNOW / REMEMBER……………….…………………………..8 | | | | | | |  |
| 2.3 | Did you feel comfortable to ask questions during the visit? | NO……………………………………………………………………………..…..0  YES………………………………………………………………………………….1 DON’T KNOW / REMEMBER……………….…………………………..8 | | | | | | |  |
| 2.4 | Do you believe that the information that you shared about yourself with the health care provider will be kept confidential? | NO……………………………………………………………………………..…..0  YES………………………………………………………………………………….1 DON’T KNOW / REMEMBER……………….…………………………..8 | | | | | | |  |
| 2.5 | Did you feel the information given to you during your visit was too little, just about right, or too much? | TOO LITTLE………………………………….………………………………….1  ABOUT RIGHT……………………………….………………………………..2 TOO MUCH……………………………………...….…………………………3  DON’T KNOW / REMEMBER…………….….………………………….8 | | | | | | |  |
| 2.6 | **During this visit, did the staff…** | | **NO** | **YES** | | **NA** | | **DK** |  |
|  | A. Interact with you in a friendly way? | | 0 | 1 | | 6 | | 8 |  |
|  | B. Pay close attention to you throughout labor and delivery? | | 0 | 1 | | 6 | | 8 |  |
|  | C. Come to attend to you if you called for help? | | 0 | 1 | | 6 | | 8 |  |
|  | D. Advise you on what you could do to make yourself more comfortable when you were in pain? | | 0 | 1 | | 6 | | 8 |  |
|  | E. Try to make you more comfortable? | | 0 | 1 | | 6 | | 8 |  |
|  | ***IF YES TO 2.6E, PROBE:***   F. What type of comfort measures did the staff provide to make you more comfortable?   ***DO NOT READ LIST***  ***MULTIPLE RESPONSES ALLOWED*** |  | | | | **NO** | | **YES** |  |
|  |  | A. RUBBED BACK | | | | 0 | | 1 |  |
|  |  | B. OFFERED FLUIDS TO DRINK | | | | 0 | | 1 |  |
|  |  | C. OFFERED FOOD TO EAT | | | | 0 | | 1 |  |
|  |  | D. ASSISTED CLIENT IN CHANGING POSITION | | | | 0 | | 1 |  |
|  |  | E. HELPED CLIENT TO WALK AROUND | | | | 0 | | 1 |  |
|  |  | F. USED ENCOURAGING WORDS | | | | 0 | | 1 |  |
|  |  | G. OTHER (SPECIFY) __________________ | | | | 0 | | 1 |  |
| 2.7 | A. Overall, how would you rate the staff’s kindness in the way they spoke to you during this visit? Would you say they were kind or unkind? | UNKIND………….………………………..…………………………….……….1  KIND ………………………………………..……………………..……………..2  NEITHER UNKIND NOR KIND……………………..………….…………3  DON’T KNOW………………………….…………….………………………..8 | | | | | | | 2.8  2.8 |
|  | B. Would you say they were very […] or a little […]?   **USE ANSWER FROM 2.7A IN PLACE OF […] (UNKIND OR KIND)** | VERY UNKIND………….…………………….………………….…………….1  A LITTLE UNKIND………………………………………..……...…………..2  A LITTLE KIND………………………………………………………….………4  VERY KIND………………………………………………………….……………5 | | | | | | |  |
| 2.8 | A. Overall, how would you rate the staff’s level of encouragement during labor and delivery? Would you say they were discouraging or encouraging? | DISCOURAGING………………………..…………………………………….1  ENCOURAGING………………………………………………..……………..2  NEITHER DISCOURAGING NOR ENCOURAGING……………….3  DON’T KNOW………………………….…………….…………………..……8 | | | | | | | 2.9  2.9 |
|  | B. Would you say they were very […] or a little […]?   ***USE ANSWER FROM 2.8A IN PLACE OF […] (DISCOURAGING OR ENCOURAGING)*** | VERY DISCOURAGING………….……….………..……………………….1  A LITTLE DISCOURAGING…………………………..……….…………..2  A LITTLE ENCOURAGING………………………………..……….………4  VERY ENCOURAGING………………………………………………………5 | | | | | | |  |
| 2.9 | A. How would you rate the facility’s level of cleanliness? Would you say it is dirty or clean? | DIRTY………………………..…………………………………….………………1  CLEAN……………………………………………………………………………..2  SATISFACTORY / NEITHER DIRTY NOR CLEAN…….…………….3  DON’T KNOW………………………….…………….……….…………….…8 | | | | | | | 2.10  2.10 |
|  | B. Would you say it is very […] or a little […]?   ***USE ANSWER FROM 2.9A IN PLACE OF […] (DIRTY OR CLEAN)*** | VERY DIRTY………….……….………..……………..……….……………….1  A LITTLE DIRTY…………………………..…………….……….……………..2  A LITTLE CLEAN……………………………………….……………….………4  VERY CLEAN…………………………………………….………………………5 | | | | | | |  |
| 2.10 | A. How would you rate your overall satisfaction with the care you received during labor and delivery? Would you say you are dissatisfied or satisfied? | DISSATISFIED………………………………………………….……………….1  SATISFIED………………………………………………………………………..2  NEITHER DISSATISFIED NOR SATISFIED……………………..…….3  DON’T KNOW………………………….…………….………………………..8 | | | | | | | 2.11  2.11 |
|  | B. Would you say you are very […] or a little […]?   ***USE ANSWER FROM 2.10A IN PLACE OF […] (DISSATISFIED OR SATISFIED)*** | VERY DISSATISFIED………….……….…….……………...……………….1  A LITTLE DISSATISFIED…………………….….………..….……………..2  A LITTLE SATISFIED………………………………………………….………4  VERY SATISFIED…………………………………………….…………………5 | | | | | | |  |
| 2.11 | Did any of the health facility staff ever emotionally abuse you during your visit? By emotional abuse, we mean, did they speak or act in an angry or condescending way that made you feel badly about yourself, degraded, embarrassed, or sad? | NO………………………………………………………………………………….0  YES………………………………………………………………………………….1  DON’T KNOW / REMEMBER…………………………………………….8  REFUSED……………………………………………………………………..….9 | | | | | | |  |
| 2.12 | Did any of the health facility staff every physically abuse you during your visit? By physical abuse, we mean, did they hit, slap, push, pinch, kick you, or use any other type of physical force against you? | NO………………………………………………………………………………….0  YES………………………………………………………………………………….1  DON’T KNOW / REMEMBER…………………………………………….8  REFUSED………………………………………………………………………...9 | | | | | | |  |
| 2.13A | What about your care are you satisfied with?  **MORE THAN ONE RESPONSE POSSIBLE** |  | | | | | **NO** | **YES** |  |
|  |  | A. STAFF WAS KIND IN THE WAY THEY TREATED ME | | | | | 0 | 1 |  |
|  |  | B. STAFF WAS ATTENTIVE TO MY NEEDS | | | | | 0 | 1 |  |
|  |  | C. STAFF WAS KNOWLEDGEABLE | | | | | 0 | 1 |  |
|  |  | D. STAFF WAS SKILLED IN PROVIDING SERVICES | | | | | 0 | 1 |  |
|  |  | E. STAFF CAME WHEN I CALLED | | | | | 0 | 1 |  |
|  |  | F. STAFF USED ENCOURAGING WORDS WHEN SPEAKING TO ME | | | | | 0 | 1 |  |
|  |  | G. STAFF COMFORTED ME WHEN I WAS IN PAIN | | | | | 0 | 1 |  |
|  |  | H. STAFF GAVE ME PAIN MEDICATION | | | | | 0 | 1 |  |
|  |  | I. STAFF GAVE ME OTHER MEDICATION OR TREATMENT (E.G., ANTIBIOTIC, IV FLUIDS, BLOOD TRANSFUSION) | | | | | 0 | 1 |  |
|  |  | J. STAFF GAVE ME FOOD TO EAT AND/OR FLUIDS TO DRINK | | | | | 0 | 1 |  |
|  |  | K. STAFF STAYED WITH ME | | | | | 0 | 1 |  |
|  |  | L. STAFF ALLOWED ME TO HAVE A BIRTH COMPANION | | | | | 0 | 1 |  |
|  |  | M. FACILITY WAS CLEAN | | | | | 0 | 1 |  |
|  |  | N. OTHER (SPECIFY): _____________________ | | | | | 0 | 1 |  |
|  |  | O. NOTHING | | | | | 0 | 1 |  |
|  |  | P. DON’T KNOW | | | | | 0 | 1 |  |
| 2.14A | What about your care are you dissatisfied with?  **MORE THAN ONE RESPONSE POSSIBLE** |  | | | | | **NO** | **YES** |  |
|  |  | A. STAFF WAS UNKIND – PHYSICALLY ABUSED ME | | | | | 0 | 1 |  |
|  |  | B. STAFF REFUSED TO GIVE ME OTHER MEDICATION OR TREATMENT (E.G., ANTIBIOTIC, IV FLUIDS, BLOOD TRANSFUSION) | | | | | 0 | 1 |  |
|  |  | C. STAFF DID NOT STAY WITH ME | | | | | 0 | 1 |  |
|  |  | D. STAFF WAS UNKIND – EMOTIONALLY ABUSED ME | | | | | 0 | 1 |  |
|  |  | E. STAFF WAS NOT ATTENTIVE TO MY NEEDS | | | | | 0 | 1 |  |
|  |  | F. STAFF WAS NOT KNOWLEDGEABLE | | | | | 0 | 1 |  |
|  |  | G. STAFF WAS NOT SKILLED IN PROVIDING SERVICES | | | | | 0 | 1 |  |
|  |  | H. STAFF DID NOT COME WHEN I CALLED | | | | | 0 | 1 |  |
|  |  | I. STAFF USED HARSH WORDS WHEN SPEAKING TO ME | | | | | 0 | 1 |  |
|  |  | J. STAFF DID NOT HELP ME WHEN I WAS IN PAIN | | | | | 0 | 1 |  |
|  |  | K. STAFF REFUSED TO GIVE ME PAIN MEDICATION | | | | | 0 | 1 |  |
|  |  | L. STAFF REFUSED TO GIVE ME FOOD TO EAT AND/OR FLUIDS TO DRINK | | | | | 0 | 1 |  |
|  |  | M. STAFF DID NOT ALLOW ME TO HAVE A BIRTH COMPANION | | | | | 0 | 1 |  |
|  |  | N. FACILITY WAS DIRTY | | | | | 0 | 1 |  |
|  |  | O. OTHER (SPECIFY): ____________________ | | | | | 0 | 1 |  |
|  |  | P. NOTHING | | | | | 0 | 1 |  |
|  |  | Q. DON’T KNOW | | | | | 0 | 1 |  |
| **SECTION 3. CLIENT FACILITY EXPERIENCES** | | | | | | | | | |
| 3.1 | About how long did you wait between the time you first arrived at this facility and the time you saw a staff person for a consultation?  ***RECORD MINUTES OR HOURS*** | A. MINUTES………………..….…………………………..………1 [ __\|__ ]  B. HOURS….………………………………………………..…..….2 [ __\|__ ]  DON’T KNOW / REMEMBER………………….……….………………88 | | | | | | |  |
| 3.2 | Do you feel that your waiting time was reasonable or too long? | NO WAITING TIME, WAS SEEN IMMEDIATELY………..……….1  REASONABLE AMOUNT OF TIME……………………………..……..2  TOO LONG………………………………………………………………..…….3  DON’T KNOW / REMEMBER………………….……………………...…8 | | | | | | |  |
| 3.3 | Did the health care staff visit you regularly during the course of labor?***REMEMBER, A BIRTH COMPANION IS NOT A HEALTH CARE PROVIDER.*** | NO……………………………………………………………………………..…..0  YES………………………………………………………………………………….1 DON’T KNOW / REMEMBER………………….…………………..……8 | | | | | | |  |
| 3.4 | Was a health care provider with you at the moment of delivery?  ***REMEMBER, A BIRTH COMPANION IS NOT A HEALTH CARE PROVIDER*.** | NO……………………………………………………………………………..…..0  YES………………………………………………………………………………….1 DON’T KNOW / REMEMBER………………….…………………..……8 | | | | | | |  |
| 3.22 | If you needed the services in the future, would you return to this health facility for maternal health services? | NO……………………………………………………………………………..…..0  YES………………………………………………………………………………….1 DON’T KNOW / REMEMBER………………….………..………………8 | | | | | | |  |
| 3.23 | Will you recommend this health facility to a friend or family member for maternal health services? | NO……………………………………………………………………………..…..0  YES………………………………………………………………………………….1 DON’T KNOW / REMEMBER……………….………………..…………8 | | | | | | |  |
| **SECTION 4. FAMILY PLANNING CARE AS PART OF POST-DELIVERY CARE** | | | | | | | | | |
| **I am going to ask you some questions about family planning; this is about contraceptive methods that couples use to delay or avoid pregnancy.** | | | | | | | | | |
| 4.1 | During this visit, did you receive any information about family planning? | NO……………………………………………………………………………..…..0  YES………………………………………………………………………………….1  DON’T KNOW…………………….………………….………………..………8 | | | | | | | 4.5  4.5 |
| 4.2 | How did you get this information?  ***DO NOT READ LIST***  ***MULTIPLE RESPONSES ALLOWED***  ***PROBE: Anything else?*** |  | | | | **NO** | | **YES** |  |
|  |  | A. PROVIDER SPOKE ABOUT FAMILY PLANNING | | | | 0 | | 1 |  |
|  |  | B. CLIENT ASKED ABOUT FAMILY PLANNING | | | | 0 | | 1 |  |
|  |  | C. SHOWN A VIDEO | | | | 0 | | 1 |  |
|  |  | D. PARTICIPATED IN A HEALTH EDUCATION TALK | | | | 0 | | 1 |  |
|  |  | E. SAW POSTERS / FLYERS | | | | 0 | | 1 |  |
|  |  | F. OTHER (SPECIFY) _________________ | | | | 0 | | 1 |  |
|  |  | G. DON’T KNOW / REMEMBER | | | | 0 | | 1 |  |
| 4.3 | Which methods were described in the information you received?  ***DO NOT READ LIST***  ***MULTIPLE RESPONSES ALLOWED***  ***PROBE: Anything else?*** |  | | | | **NO** | | **YES** |  |
|  |  | A. COMBINED ORAL PILL | | | | 0 | | 1 |  |
|  |  | B. PROGESTIN-ONLY PILL | | | | 0 | | 1 |  |
|  |  | C. PILL (TYPE UNSPECIFIED) | | | | 0 | | 1 |  |
|  |  | D. PROGESTIN-ONLY INJECTABLE (2-3 MONTHLY) | | | | 0 | | 1 |  |
|  |  | E. MALE CONDOM | | | | 0 | | 1 |  |
|  |  | F. FEMALE CONDOM | | | | 0 | | 1 |  |
|  |  | G. IUD / IUCD | | | | 0 | | 1 |  |
|  |  | H. IMPLANT | | | | 0 | | 1 |  |
|  |  | I. EMERGENCY CONTRACEPTION | | | | 0 | | 1 |  |
|  |  | J. FEMALE STERILIZATION (TUBAL LIGATION) | | | | 0 | | 1 |  |
|  |  | K. MALE STERILIZATION (VASECTOMY) | | | | 0 | | 1 |  |
|  |  | L. WITHDRAWAL | | | | 0 | | 1 |  |
|  |  | M. CYCLE BEADS FOR STANDARD DAYS METHOD (SDM) | | | | 0 | | 1 |  |
|  |  | N. NATURAL METHODS (PERIODIC ABSTINENCE) | | | | 0 | | 1 |  |
|  |  | O. LACTATIONAL AMENORRHEA (LAM) | | | | 0 | | 1 |  |
|  |  | P. OTHER (SPECIFY) _________________ | | | | 0 | | 1 |  |
|  |  | Q. DON’T KNOW / REMEMBER | | | | 0 | | 1 |  |
| 4.5 | Did you receive a contraceptive method, or a prescription, or a referral for a method during this visit? | NO, …………………………………………..…………………………………...0  YES, RECEIVED METHOD………………………………………………….1  YES, RECEIVED PRESCRIPTION…………………………………………2  YES, RECEIVED REFERRAL………………………………………………..3 | | | | | | | 6.1 |
| 4.6 | What method did you receive? | COMBINED ORAL PILL………………………………………………..…...1  PROGESTIN-ONLY PILL………………………………………..……..……2  PILL (TYPE UNSPECIFIED) ………………………………………….....…3  PROGESTIN-ONLY INJECTABLE (2-3 MONTHLY)…….…….....4  MALE CONDOM………………………………………………………………5  FEMALE CONDOM……………………………………………...……..…..6  IUD / IUCD…………………………………………………………………...…7  IMPLANT……………………………………………………………………..….8  EMERGENCY CONTRACEPTION………………………………….…...9  FEMALE STERILIZATION (TUBAL LIGATION)………………......10  MALE STERILIZATION (VASECTOMY)………………………..….…11  OTHER (SPECIFY) _________________________……..……77  DON’T KNOW / REMEMBER………………….…………………….…88 | | | | | | |  |

| **SECTION 6. DEMOGRAPHIC INFORMATION** | | | | | | | |
| --- | --- | --- | --- | --- | --- | --- | --- |
| 6.1 | In what month and year were you born?  ***IF KNOWN, ENTER BOTH MONTH AND YEAR*** | A. MONTH………………………………………….…..…….…. [ __\|__ ]  B. YEAR……………………………………………….… [ __\|__\|__\|__ ]  DON’T KNOW MONTH / REMEMBER………….….……………88  DON’T KNOW YEAR / REMEMBER……………...……………8888 | | | | |  |
| 6.2 | Have you ever attended school? | NO……………………………………………………………………………..…..0  YES………………………………………………………………………………….1 | | | | | 6.7 |
| 6.3 | What is the highest level of education you attended? | PRIMARY…………………………………………………………………………1  POST-PRIMARY / VOCATIONAL……………………………………….2  SECONDARY……………………………………………..…………………….3  COLLEGE (MIDDLE LEVEL)……………………………………………….4  UNIVERSITY…………………………………………………….………………5 | | | | |  |
| 6.7 | Do you know how to read or write? | NO……………………………………………………………………………..…..0  YES, READ AND WRITE…………………………………………………...1  YES, READ ONLY……………………………………………………………..2 | | | | |  |
| 6.8 | How often do you attend religious services? | DOES NOT ATTEND AT ALL……………………………………………..0  SEVERAL TIMES A DAY…………………………………………………….1 ONCE A DAY……………………………………………………………..…….2 AT LEAST ONCE A WEEK………………………………………………….3 AT LEAST ONCE A MONTH………………………………………………4 LESS THAN ONCE A MONTH……………………………………………5 ONLY FOR SPECIAL OCCASIONS………………………………………6  DON’T KNOW / REMEMBER……………….………………………..…8  DECLINED TO ANSWER……………………………………………....….9 | | | | |  |
| 6.9 | What is the town and village where you currently live? | A. NAME OF TOWN ________________________________  B. NAME OF VILLAGE ______________________________ | | | | |  |
| 6.10 | What is the main material of the floor in your household? | EARTH, SAND, DUNG……….……………………………………….…….1  WOOD, PLANKS, BAMBOO, PALM…………………………….…….2  PARQUET OR POLISHED WOOD………………………………………3  VINYL OR ASPHALT STRIPS………………………………………….…..4  CERAMIC TILES, TERRAZZO……………………………………………..5  CEMENT………………………………………………………………………….6  CARPET…………………………………………………………………………..7  OTHER (SPECIFY) ____________________________.......77 | | | | |  |
| 6.11 | What is the wall material of your household? | GRASS……………………..……….…………………………………………….1  POLES AND MUD…………………………………………………………….2  SUN-DRIED BRICKS………………….………………………………………3  BACKED BRICKS………………………………………………………….…..4  WOOD, TIMBER……………….……………………………………………..5  CEMENT BLOCKS…………………………………………………………….6  STONES…………………………………………………………………………..7  OTHER (SPECIFY) ____________________________......77 | | | | |  |
| 6.12 | What is the roofing material of your household? | GRASS, THATCH, MUD…………………..……………………………….1  IRON SHEETS……….……………………..………………………………….2  TILES…………………..………………….………………………………………3  CONCRETE…………..…………………………………………………….…..4  ASBESTOS……………………….……………………………………………..5  OTHER (SPECIFY) ____________________________........7 | | | | |  |
| 6.13 | How many rooms in your household are used for sleeping? | ROOMS…………………………………………………..….……. [ __\|__ ] | | | | |  |
| 6.14 | **Does anyone of your household own:** | | | **NO** | **YES** | **DK** |  |
|  | A. A watch? | | | 0 | 1 | 8 |  |
|  | B. A bicycle? | | | 0 | 1 | 8 |  |
|  | C. A motorcycle or motor scooter? | | | 0 | 1 | 8 |  |
|  | D. A car or truck? | | | 0 | 1 | 8 |  |
|  | E. A traditional or mobile (e.g., M-pesa) bank account? | | | 0 | 1 | 8 |  |
| 6.15 | What kind of toilet facility do members of your household usually use? | FLUSH / POUR FLUSH TO PIPED SEWER SYSTEM…………….1  FLUSH / POUR FLUSH TO PIPED SEPTIC TANK…………….…..2  FLUSH / POUR FLUSH TO ELSEHWERE………………………….…3  VENTILATED IMPROVED PIT LATRINE (VIP)…………….……….4  PIT LATRINE WITH SLAB………………………………………………….5  PIT LATRINE WITHOUT SLAB / OPEN PIT……………………..….6  COMPOSTING TOILET / ECOSAN……………………………….…...7  BUCKET(S).……………………………………………………………….….…8  NO FACILITY / BUSH / FIELD…………………………………….……..9  OTHER (SPECIFY) ____________________________......77 | | | | |  |
| 6.16 | Do you share this toilet facility with other households? | NO……………………………………………………………………………..…..0  YES………………………………………………………………………………….1 DON’T KNOW / REMEMBER………………….………………..………8 | | | | | 6.18  6.18 |
| 6.17 | How many households use this toilet facility? | HOUSEHOLDS……………………………………………………. [ __\|__ ]  DON’T KNOW / REMEMBER………………….………………………88 | | | | |  |
| 6.18 | **Does your household have:** | | **NO** | | **YES** | **DK** |  |
|  | A. Electricity? | | 0 | | 1 | 8 |  |
|  | B. A paraffin lamp? | | 0 | | 1 | 8 |  |
|  | C. A radio? | | 0 | | 1 | 8 |  |
|  | D. A television? | | 0 | | 1 | 8 |  |
|  | E. A mobile telephone? | | 0 | | 1 | 8 |  |
|  | F. A non-mobile telephone (land line)? | | 0 | | 1 | 8 |  |
|  | G. An iron (charcoal or electric)? | | 0 | | 1 | 8 |  |
|  | H. A refrigerator? | | 0 | | 1 | 8 |  |
| 6.19 | What type of fuel does your household mainly use for cooking? | NO FOOD COOKED IN HOUSEHOLD………….………...….….….0  ELECTRICITY……………………………………………….………...….…...1  BOTTLED GAS…………………………………………….………...….…...2  PARAFFIN / KEROSENE…………………………….….…………….…..3  CHARCOAL……………………………………………….….…………….….4  FIREWOOD……………………………………………….….…..….……..…5  CROP RESIDUALS, STRAW, GRASS…………….….……….…....…6  ANIMAL DUNG………………………………………….…..…..….….…..7  OTHER (SPECIFY) ____________________________.....77 | | | | |  |
| 6.20 | What is the main source of energy for lighting in your household? | ELECTRICITY………………………………….…………………….….…..…1  SOLAR……….…………………………………………………….…….………2  GAS…………………………………………………………..…………..………3  PARAFFIN-HURRICANE LAMP…..…..………………………….…...4  PARAFFIN-PRESSURE LAMP……….….…….………………..…...…5  PARAFFIN-WICK LAMP………………………….………….……...……6  FIREWOOD…………………………………………….……………....….…7  CANDLES………………………………………………….………...……...…8  OTHER (SPECIFY) _____________________________...77 | | | | |  |
| 6.21 | Are you currently married or living with a man as if married? | NO, NOT IN A UNION…………………………………….……………...0  YES, CURRENTLY MARRIED………………………………….…..….…1  YES, LIVING WITH A MAN………………………………….…….…….2 | | | | | 8.1 |
| 6.22 | Have you ever been married or lived together with a man as if married? | NO………………………….…………………………………………......….….0  YES, FORMERLY MARRIED……………..…………………….…...…..1  YES, LIVED WITH A MAN…………………………..…………...…...…2 | | | | | 8.1 |
| 6.23 | What is your marital status now: Are you widowed, divorced, or separated? | WIDOWED…………………………………………………………..……...…1  DIVORCED…………………………………………………………...……..…2  SEPARATED………………………………………………………….….....…3 | | | | |  |
| **SECTION 8. PREGNANCY HISTORY** | | | | | | | |
| 8.1 | Including this pregnancy, how many times have you been pregnant? | NUMBER OF PREGNANCIES……………..…….…..……… [ __\|__ ] | | | | |  |
| 8.3 | How many times have you had a stillbirth, ectopic pregnancy, miscarriage, or induced abortion?  ***RECORD NUMBER FOR EACH CATEGORY*** | A. NUMBER OF STILLBIRTHS………………….………...….[ __\|__ ]  B. NUMBER OF ECTOPIC PREGNANCIES……………… [ __\|__ ]  C. NUMBER OF MISCARRIAGES OR ABORTIONS….. [ __\|__ ] | | | | |  |
| 8.4 | How many children have you given birth to who live with you now? | NUMBER OF CHILDREN LIVING WITH YOU…………. [ __\|__ ] | | | | |  |
| 8.5 | How many children have you given birth to who live somewhere else? | NUMBER OF CHILDREN LIVING ELSEWHERE………. [ __\|__ ] | | | | |  |
| 8.6 | Have you ever had a child born alive who later died, including those who may have died in the first hours or days after birth? | NO………………………………………………………………….......……..…0  YES…………………………………………………………………..………….…1 | | | | | 8.8 |
| 8.7 | How many children have you given birth to who later died? | NUMBER OF CHILDREN WHO DIED…………….……… [ __\|__ ] | | | | |  |
| 8.8 | So altogether you had a total of ***(ADD NUMBER OF CHILDREN FROM Q8.4+Q8.5+Q8.7)*** live births? | LIVE BIRTHS…………………………………………………..…… [ __\|__ ] | | | | |  |

Thank you for taking the time to speak to me today.
