# Supplementary material for: Birth companionship in a government health system: a pilot study in Kigoma, Tanzania
Source: BMC Pregnancy Childbirth. 2021 Apr 16;21:304. doi: 10.1186/s12884-021-03746-0 (PMC8052747; doi:10.1186/s12884-021-03746-0)
Supplement: Supplementary file 1 — Additional file 1. [file 12884_2021_3746_MOESM1_ESM.zip › D. Implementation Research Guide_Women with BC_UPDATEDR4.doc]

**Implementation Research FGD Guide**

**Women who delivered with a companion**

General Information:

| District Name | Ward | Village/Facility Name |
| --- | --- | --- |
|  |  |  |
| Date of Interview | Time of Commencement | Time of Completion |
|  |  |  |
| Name of the facilitator | Name of Reporter | Name of observer |
|  |  |  |

1. How were you informed about birth companions in your community? (PROBE: Who communicated it to you and how? At ANC, through community leaders, CHWs, friends, relatives, radio ads, billboards, or posters, meetings, etc.?)
2. Can you please tell me who your birth companion was during labor and delivery?
   a. What is your relationship with the companion?
3. How did you choose this companion?[PROBE: why they chose this particular person-including why some women choose repeat DBCs/TBAs

ii. Probe on whether the companion was chosen based on ability to keep secrets/confidentiality- (beyond relying on the person for financial support or knowledge of childbirth)

a. Who was involved in the decision-making, and why where these people involved?[ PROBE on how the decision is made and who determines how these decisions are made-is it according to individuals, families, communities, or village governments especially in instances where repeat DBAs/TBAs are pre-selected)

4. Was the role of the birth companion discussed with you? (PROBE: When did you learn the rules of what a companion could do or could not do?)
5. At what stage in your pregnancy did you choose to have a birth companion? (PROBE:At ANC, which visit, during labor and delivery?)
6. How did you feel having this birth companion around during labor and delivery? (PROBE:Did the person make youfeel comfortable or uncomfortable? Did the BC do things that you appreciated?)
7. What, if any,type of assistance did you get from the birth companion:[Let the participants explain what the BCs *actually* did to support them]
a. During pregnancy?
b. Duringlabor?
c. During delivery?
d. After delivery, pre-discharge?
e. After delivery, post-discharge?

[PROBE: i. on *how* the BC provided comfort, encouragement, soothed the women, made them feel not alone, gave the women confidence

[ii. Probe on what support women appreciated/valued and why?]

8. In your opinion, how did the providers interact with the birth companions?
   a. Did your birth companion assist the providers at any point in your labour and/or delivery?[PROBE: what did they do exactly? how did they work together to make the woman more cooperative, for example when it was time to push?]
9. Several renovations were made to help provide more privacy for women, providers and birth companions – what did you think of these new renovations and were they useful to you during your labour and delivery? (PROBE: Adequate space, heat/temperature, privacy, etc.)
10. What is your opinion on the supplies and equipment provided for birth companions – for infection prevention measure, uniforms for on-call companions, etc.? (PROBE: Were they used, useful, clean, etc.?)
11. Are there any things that the BC did that you feel she should not have done? (PROBE: Lack of support, how they interacted with providers?)
12. What would you tell your friend about having a birth companion?
13. What could the facility do to make it easier for the companion to provide the support you need during childbirth?
14. Are there other areas which you feelstill need to be improved regarding birth companions?[If supported by DBCs, probe further on possible obstacles for instance issues of lack of IDs, lack of training or unpreparedness, unavailable on the day of delivery]
15. If you decide to have more children, would you want to have a birth companion again? Why?
