# Supplementary material for: Birth companionship in a government health system: a pilot study in Kigoma, Tanzania
Source: BMC Pregnancy Childbirth. 2021 Apr 16;21:304. doi: 10.1186/s12884-021-03746-0 (PMC8052747; doi:10.1186/s12884-021-03746-0)
Supplement: Supplementary file 1 — Additional file 1. [file 12884_2021_3746_MOESM1_ESM.zip › F. Implementation Research IDI_BCsR4.doc]

**IDI Guide**

**Birth Companions**

General Information:

| District Name | Ward | Village/Facility Name |
| --- | --- | --- |
|  |  |  |
| Date of Interview | Time of Commencement | Time of Completion |
|  |  |  |
| Name of Facilitator | Name of Reporter | Name of Observer |
|  |  |  |

Participant:

| Age | Sex | Education Level | Marital Status | Number of Children |
| --- | --- | --- | --- | --- |
|  |  |  |  |  |

**Questions if Birth Companions are Facility-Based (On-Call):**

1. How long have you been working as a birth companion?
   a. How did you become a birth companion?
   b. What motivated you to become a birth companion?
2. What do you think the role of a birth companion is?
3. Did you receive any training?

[IF YES:]

a. When and where did you receive this training?
b. For how long, and how frequently?
c. What was the content of your training?[Probe OBCs about the doula/comfort measure support training they received, and if/how they are able to incorporate these method]

4. Is there any information/skills/knowledge that youdo not have, butfeel would be helpful to know when supporting women? If so, please elaborate.
5. What has been your experience supporting women during labor and delivery?
   a. Are you present the whole time a woman is in labor?
   b. Are you present during delivery?

[IF NO:]

i. Why not?
c. What type of support do you offer women?[PROBE on psychosocial support provided, explore further on *how* the BC provided comfort, encouragement, soothed the women, made them feel not alone, gave the women confidence]

ii. Probe on what exactly the OBCs did or did not do to offer support]

d. Can you describe your interactions with the health facility staff?[Probe on how BCs work together with providers to make the woman cooperative, for example when it is time to push]
   i. How do they treat you?
   ii. Are there any particular behaviors or things that service providers have done that you feel they should not have done?

[IF YES:]

1. Can you state some of them?
e. Are there any limitations on where you can enter or not enter in the facility? (For example, labor ward or maternity ward?)
f. Do you help service providers in any way beyond supporting women during labor and delivery?Explore further, on what other roles OBCS are doing which go beyond assisting the women.[IF YES:]
   i. Can you give examples of the kind of support you provide to service providers?[Probe on supervisory roles to DBCs, monitoring and assessing DBCs competency, checking for IDs and Screening]

Probe on whether OBCs were asked to assume a supervisory/gatekeeper role over DBCs, or did they take it upon themselves to play these roles. (OBCs, providers)

6. In your opinion, what are some of the benefits of having a birth companion around during labor and delivery? [PROBE: For women? For health providers?]

Probe further when respondents state that *women provide certain information* to BCs that they normally withheld from providers. Probe on whether withholding of information is intentional or if she did not think it was not relevant

7. What aspects of the facility environment do or do not accommodate birth companions? [PROBE: Physical environment? Patient workflow?]
8. Are there any challenges that you have experienced in fulfilling your role as a birth companion?

[IF YES:]

a. What were they?[PROBE if anything has been done to address the challenges]
b. Are there any suggestions for dealing with these challenges?

9. Would you recommend implementation of the birth companionship program to other facilities in Tanzania? Why or why not?
10. Are there other areas that you feel need to be improved regarding birth companions?

**Questions ifBirth Companions are Family Members/Friends:**

11. I understand you accompanied a woman to the facility for labor and delivery. I would like to ask you about that experience. Can we begin with you telling me what your relationship is with the woman you accompanied to the facility?
12. How many times have you supported women during labor/delivery in the last six months?
    a. How many women in facilities?
    b. How many women at home?
13. Before you were selected as a birth companion, had you heard that women were allowed to have a birth companion with them in facilities?

[IF YES:]

a. How did you hear about it?
b. What did you hear about it?

14. How were you chosen as a birth companion to accompany the woman you supported?
    a. Was it a planned or spontaneous decision?[How was the ultimate decision made?]
    b. Who selected you? Please describe everyone included in the decision-making process. [Who determines how these decisions are made-is it according to individuals, household/family, community (as culture and society dictates), and village government (in instances where repeat DBCs/TBAs are pre-selected for the community)?
15. What do you think the role of a birth companion is?
16. Did you receive any orientation prior to supporting the woman you helped?

[IF YES:][PROBE from whom]

a. When and where did you receive this orientation?
b. For how long, and how frequently?
c. What topics were covered?

17. Is there any information/skills/knowledge that you did not have, but feel would have been helpful to know before the birth? If so, please elaborate.
18. What was your experience when you got to the facility?
    a. Were you there the whole time the woman you supported was in labor?
    b. Were you there during her delivery?
    c. What did you do to support the woman?[PROBE on*psychosocial support*-*how* the BC provided comfort, encouragement, soothed the women, made them feel not alone, gave the women confidence ( for the women, providers)

ii Probe further on what exactly the DBCs did or did not to do to offer support to women

d. Can you describe your interactions with the health facility staff?[Probe on how BCs work together with providers to make the woman cooperative, for example when it is time to push]
   i. How did they treat you?
   ii. Were there any particular behaviors or things that service providers did that you feel they should not have done?

[IF YES:]

1. Can you state some of them?
e. What were your interactions with the other birth companions who sit in the facility?
   i. Did they help or guide you in any way?[PROBE on “rapid training” assessment and checking of IDs, supervised or monitored by OBCs]
f. Were there any limitations on where you could enter or not enter in the facility? (For example, labor ward or maternity ward?)
g. Did you help service providers in any way beyond supporting the woman during labor and delivery?

[IF YES:]

i. Can you give examples of the kind of support you provided to service providers?

19. In your opinion, what are some of the benefits of having a birth companion around during labor and delivery? [PROBE: For women? For health providers?]

Probe further when respondents state that women provide certain information to BCs that they normally withheld from providers.

- Probe on whether withholding of information is intentional or if she didn’t think it wasn’t relevant

20. Are there other areas that you feel need to be improved regarding birth companions?
