# Supplementary material for: Birth companionship in a government health system: a pilot study in Kigoma, Tanzania
Source: BMC Pregnancy Childbirth. 2021 Apr 16;21:304. doi: 10.1186/s12884-021-03746-0 (PMC8052747; doi:10.1186/s12884-021-03746-0)
Supplement: Supplementary file 1 — Additional file 1. [file 12884_2021_3746_MOESM1_ESM.zip › G.Provider BIRTH COMPANION_ENGLISHR4.doc]

| **PROVIDER INTERVIEW – KIGOMA REGION, TANZANIA** | | | |
| --- | --- | --- | --- |
|  | Date of Interview: | A. DAY…………………………………………………………..…. [ __\|__ ]  B. MONTH……………………………………………………..…. [ __\|__]  C. YEAR……………………………………………..…. [ __\|__\|__\|__ ] |  |
|  | Time at Start of Interview: | 24 HOUR CLOCK………………………………..…… [ __\|__:__\|__ ] |  |
|  | Interviewer Code: | …………………………………………………….……….. [ __\|__\|__\|__ ] |  |
|  | Supervisor Code: | …………………………………………………………………..……... [ __\|__] |  |
|  | Unique Provider ID: | ……………………………………………… [ 4\| __\|__\| __\|__\|__\|__ ] |  |
|  | Facility Name: | _______________________________________________ |  |
|  | Facility Code: | ………………………………………………………….….. [ __\|__\|__\|__ ] |  |
|  | Facility Type: | HOSPITAL……………………………………………………………………..1  HEALTH CENTER……………………………………………………….….2 |  |
|  | District: | BUHIGWE……………………………………………………………………..1  KABONDO……………………………………………..……………………..2  KAKONKO……………………………………………………………………..3  KASULU………………………………………………………………………..4  KIGOMA………………………………………………………………..……..5  UVINZA…………………………………………………………………………6 |  |
|  | Time at End of Interview: | 24 HOUR CLOCK………….…………………..…… [ __\|__:__\|__ ] |  |
|  | Result Code: | COMPLETED…………………………………………………………………1  INCOMPLETE (SPECIFY RETURN DATE)______________.2  POSTPONED (SPECIFY DATE)_____________________..3  REFUSED…………………………….………………………………………..9  OTHER (SPECIFY) _____________________________....7 |  |
|  | Date of Data Entry: | A. DAY………………………………………………………….…. [ __\|__ ]  B. MONTH………………………………………………..…..…. [ __\|__]  C. YEAR……………………………………………..…. [ __\|__\|__\|__ ] |  |
|  | Data Entry Clerk Code: | ………………………………………………………………..……... [ __\|__] |  |

| **INTRODUCTION AND CONSENT**  Hello. My name is ___________. I am working with the Ministry of Health, Community Development, Gender, Elderly and Children, ThaminiUhai, and AMCA. We are conducting a survey about health services in Kigoma. The information we collect will help the government to improve the quality of health services. The survey usually takes about 30minutes. Your answers will be confidential and will not be shared with anyone except members of our survey team. You don’t have to participate, but we hope you will agree since your views are important. You will not benefit directly from participating. There are no risks to you, but you may feel uncomfortable with some questions. If I ask you any question you don’t want to answer, let me know and I will go on the next question or you can stop the interview at any time. Do you have any questions? May I begin now? | | |
| --- | --- | --- |
| **SIGNATURE OF INTERVIEWER:** _______________________________________ DATE: __________________________ | | |
| Result of Consent | RESPONDENT DOES NOT AGREE TO BE INTERVIEWED……0 RESPONDENT AGREES TO BE INTERVIEWED……………………1 | END |

| **PARTICIPANT ELIGIBILITY / SCREENING QUESTIONS** | | | |
| --- | --- | --- | --- |
| **Number** | **Question** | **Response Options** | **Skip** |
| E1 | How old were you on your last birthday? | AGE IN YEARS…………………………………….... [ __\|__ ]  DON’T KNOW / REMEMBER………………….……..…88 | END IF LESS THAN 18 YEARS |
| E2 | Are you a health care provider at this facility? | NO………………………………………………………….…......0  YES…………………………………………………………..….….1 | END |
| E3 | What is your current occupational category or qualification (or cadre)?  ***ONLY READ THE FOLLOWING IF PROVIDER DOES NOT IMMEDIATELY RESPOND TO THE QUESTION***  **For example are you a registered nurse, midwife, or generalist medical doctor.** | MEDICAL DOCTOR…………………………………….…….1  MEDICAL DOCTOR, SPECIALIST………………….…….2  ASSISTANT MEDICAL OFFICER (AMO)………….…..3  CLINICAL OFFICER (CO) / CLINICAL ASSISTANT...4  NURSE OFFICER / ASSISTANT NURSE OFFICER / REGISTERED NURSE (RN) / ENROLLED NURSE.…5  NURSE MIDWIFE……………………………………………..6  MCH AIDE…………………………………………………..…..7  NURSE / MEDICAL ASSISTANT….………….………....8  NURSE / MEDICAL ATTENDANT…………………….…9  MEDICAL RESIDENT……………………………………...10  NURSING RESIDENT…………………………….….….…11  OTHER (SPECIFY) ____________________..…12 |  |

| **SECTION 1. DEMOGRPAHIC INFORMATION** | | | | | | | | | | | | |
| --- | --- | --- | --- | --- | --- | --- | --- | --- | --- | --- | --- | --- |
| **Number** | **Question** | | **Response Options** | | | | | | | | | **Skip** |
| 1.0 | What is the sex of the provider? | | Male………………………………………………………………………..……….0  Female…………………………………………………………………….……….1 | | | | | | | | |  |
| 1.1 | What year did you graduate or complete this qualification?  ***IF NO TECHNICAL QUALIFICATION, ASK:***  **What year did you complete any basic training for your current occupational category?** | | GRADUATION YEAR………………………... [___] [___] [___] [___]  DON’T KNOW YEAR / REMEMBER………………….……………8888 | | | | | | | | |  |
| 1.2 | What is the highest level of school you attended? | | PRIMARY………………………………………………………………..…………1  POST-PRIMARY / VOCATIONAL………………………………..……….2  SECONDARY……………………………………………..………………..…….3  COLLEGE (MIDDLE LEVEL)……………………………………………..….4  UNIVERSITY……………………………………………………….…………..…5 | | | | | | | | |  |
| 1.3 | What is the highest level of school you completed? | | PRIMARY…………………………………………………………………..………1  POST-PRIMARY / VOCATIONAL…………………………………..…….2  SECONDARY……………………………………………..…………………..….3  COLLEGE (MIDDLE LEVEL)……………………………………………..….4  UNIVERSITY……………………………………………………….…………..…5 | | | | | | | | |  |
| 1.4 | In what month and year did you start working at this facility as a **[insert cadre from QE3]?** | | A. MONTH………………………………………………………..…. [ __\|__]  B. YEAR………………………………………………..…. [ __\|__\|__\|__ ]  DON’T KNOW MONTH / REMEMBER………………….…………88  DON’T KNOW YEAR / REMEMBER………………….….………8888 | | | | | | | | |  |
| 1.5 | Do you provide the following services on a regular basis…  ***READ LIST*** | | | | **NO** | | **YES** | | **DK** | | |  |
|  | A. Family planning? | | | | 0 | | 1 | | 8 | | |  |
|  | B. Antenatal care? | | | | 0 | | 1 | | 8 | | |  |
|  | C. Labor and delivery? | | | | 0 | | 1 | | 8 | | |  |
|  | D. Postnatal care? | | | | 0 | | 1 | | 8 | | |  |
|  | E. Post-abortion care? | | | | 0 | | 1 | | 8 | | |  |
|  | F. Newborn or well-baby care? | | | | 0 | | 1 | | 8 | | |  |
| **SECTION 3. LABOR AND DELIVERY CARE** | | | | | | | | | | | | |
| **Number** | | **Question** | | **Response Options** | | | | | | | **Skip** | |
| 3.3 | | How many deliveries did you attend last month? | | DELIVERIES………………………….………………….…. [ __ ] [ __ ]  DON’T KNOW / REMEMBER, BUT LESS THAN 10……..66  DON’T KNOW /REMEMBER, BUT 10 OR MORE……..…77  DON’T KNOW / REMEMBER……………….……………………88 | | | | | | |  | |
| 3.3A | | Did your facility’s maternity ward undergo renovations to improve privacy for women in labor/delivery? | | NO………………………………………………………………….……..…..0  YES………………………………………………………………….………….1 DON’T KNOW / REMEMBER……………….…………….…..……8 | | | | | | | 3.4  3.4 | |
| 3.3B | | ***IF YES:*** Did the renovations:  ***READ EACH QUESTION BELOW*** | |  | | **NO** | | **YES** | | **DK** |  | |
|  |  | A. Increase privacy related to what could be heard? | | A. INCREASE AUDIO PRIVACY | | 0 | | 1 | | 8 |  | |
|  |  | B. Increase privacy related to what could be seen? | | B. INCREASE VISUAL PRIVACY | | 0 | | 1 | | 8 |  | |
|  |  | C. Make your work easier? | | C. MAKE WORK EASIER | | 0 | | 1 | | 8 |  | |
|  |  | D. Make your work more difficult? | | D. MAKE WORK MORE DIFFICULT | | 0 | | 1 | | 8 |  | |
|  |  | E. Make your clients happy? | | E. MAKE YOUR CLIENTS HAPPY | | 0 | | 1 | | 8 |  | |

| ***READ:***Now I am going to ask you questions about birth companions during labor, before the baby is born. | | | | | | | | | | | | | |
| --- | --- | --- | --- | --- | --- | --- | --- | --- | --- | --- | --- | --- | --- |
| 3.4 | Do you allow women to bring a companion / support person into the labor room? | | | NO………………………………………………………………….……..…..0  YES………………………………………………………………….………….1 DON’T KNOW / REMEMBER……………….…………….…..……8 | | | | | | | | | 3.7  3.7 |
| 3.5 | Do you allow a female companion / support person, a male companion / support person, or both? | | | FEMALE ONLY…………………………………………………….……….1  MALE ONLY………………………………………………………..….……2  BOTH MALE AND FEMALE……………………………………….….3  DON’T KNOW / REMEMBER……………….……………….………8 | | | | | | | | |  |
| 3.5A | During labor before the baby is born, do you allow women to have: | | |  | | | | **NO** | | | **YES** | **DK** |  |
|  | A. A companion or support person who the woman knows and brought from the community, who received a birth companionship orientation and ID badge | | | A. BROUGHT FORM HOME WITH ORIENTATION AND BADGE | | | | 0 | | | 1 | 8 |  |
|  | **ONLY ASK THIS QUESTION IF THE FACILITY HAS THE BIRTH COMPANION PROJECT**   B. A companion or support person who the woman knows and brought from the community, who did not receive a birth companionship orientation or ID badge | | | B. BROUGHT FORM HOME WITHOUT ORIENTATION AND BADGE | | | | 0 | | | 1 | 8 |  |
|  | C. An on-call companion, based at your facility, who wears a grey uniform | | | C. ON-CALL WITH GRAY UNIFORM | | | | 0 | | | 1 | 8 |  |
|  | D. Other | | | D. OTHER SPECIFY: _________________________ | | | | 0 | | | 1 | 8 |  |
| 3.5B | Why do you allow a woman to bring a companion / support person into the labor room?  ***CIRCLE ALL SPONTANEOUS ANSWERS AND PROBE:* Anything else?** | | |  | | | | | | **NO** | | **YES** |  |
|  |  |  |  | A. HELPS CLIENT FEEL MORE COMFORTABLE | | | | | | 0 | | 1 |  |
|  |  |  |  | B. ALLOWS PROVIDER TO BE WITH OTHER CLIENTS | | | | | | 0 | | 1 |  |
|  |  |  |  | C. HELPS PROIVDER WITH WORKLOAD | | | | | | 0 | | 1 |  |
|  |  |  |  | D. TELLS PROVIDER IF THERE IS A CHANGE OR A PROBLEM | | | | | | 0 | | 1 |  |
|  |  |  |  | E. GOVERNMENT POLICY ALLOWS IT | | | | | | 0 | | 1 |  |
|  |  |  |  | F. FACILITY POLICY ALLOWS IT | | | | | | 0 | | 1 |  |
|  |  |  |  | G. GETS THE CLIENT WHAT SHE NEEDS (EG, FOOD, DRINK) | | | | | | 0 | | 1 |  |
|  |  |  |  | H. GIVES CLIENT EMOTIONAL SUPPORT | | | | | | 0 | | 1 |  |
|  |  |  |  | I. GIVES CLIENT ADVICE | | | | | | 0 | | 1 |  |
|  |  |  |  | J. OTHER (SPECIFY) ________________ | | | | | | 0 | | 1 |  |
|  |  |  |  | K. DON’T KNOW / REMEMBER | | | | | | 0 | | 1 |  |
| 3.5C | A. Would you say that allowing companions in labor has been satisfying or dissatisfying for you as a provider? | | | DISSATISFYING………………………………………………….….……….1  SATISFYING………………………………………………………….………..2  NEITHER DISSATISFYING NOR SATISFYING…………….……...3  DON’T KNOW………………………….…………………………….………8 | | | | | | | | | 3.5D  3.5D |
|  | B. Would you say it has been very *[…]* or a little *[…]*?   ***USE ANSWER FROM 3.5C_A IN PLACE OF […] (DISSATISFIED OR SATISFIED)*** | | | VERY DISSATISFIED………….…….…………………...……..….…….1  A LITTLE DISSATISFIED……………………..………..….…..….……..2  A LITTLE SATISFIED……………………………………………..…..……4  VERY SATISFIED………………………………………….…………………5  DON’T KNOW………………………….……..……………………….……8 | | | | | | | | |  |
| 3.5D | A. Would you say that allowing companions in labor has been unhelpful to you as a provider or helpful to you? | | | UNHELPFUL………………………………………………….…….….…….1  HELPFUL……………………………………………………………….……..2  NEITHER UNHELPFUL NOR HELPFUL…………………….……...3  DON’T KNOW………………………….……………………………………8 | | | | | | | | | 3.5E  3.5E |
|  | B. Would you say it has been very *[…]* or a little *[…]*?   ***USE ANSWER FROM 3.5D_A IN PLACE OF […] (UNHELPFUL OR HELPFUL)*** | | | VERY UNHELPFUL………….…….…………………...……….…….….1  A LITTLE UNHELPFUL……………………..………..…..……………..2  A LITTLE HELPFUL……………………………………….……….………4  VERY HELPFUL………………………………………….….………………5  DON’T KNOW………………………….……..……………………………8 | | | | | | | | |  |
| 3.5E | Would you say that allowing companions in labor has made it harder for you to give good quality maternity care, has not changed your ability to give good quality maternity care, or has improved your ability to give good quality maternity care? | | | HAS MADE IT HARDER TO GIVE GOOD QUALITY CARE….1  HAS NOT CHANGED ABILITY TO GIVE GOOD QUALITY  CARE…………………………………………………………………………….2  HAS IMPROVED ABILITY TO GIVE GOOD QUALITY CARE.3  DON’T KNOW………………………….……..…………………………….8 | | | | | | | | |  |
| 3.5F | Would you say that allowing companions in labor has not met your expectations, met your expectations, or exceeded your expectations? | | | NOT MET EXPECTATIONS…………………………………….……….1  MET EXPECTATIONS……………………………………………………..2  EXCEEDED EXPECTATIONS…………………………………………...3  DON’T KNOW………………………….……..………………….…………8 | | | | | | | | | 3.8 |
| 3.7 | Why do you not allow a woman to bring a companion / support person into the labor room?  ***CIRCLE ALL SPONTANEOUS ANSWERS AND PROBE:* Anything else?** | | |  | | | | | | **NO** | | **YES** |  |
|  |  |  |  | A. ROOM IS TOO SMALL | | | | | | 0 | | 1 |  |
|  |  |  |  | B. PROBLEMS WITH PRIVACY | | | | | | 0 | | 1 |  |
|  |  |  |  | C. DON’T WANT COMPANION TO BRING HERBS | | | | | | 0 | | 1 |  |
|  |  |  |  | D. GOVERNMENT POLICY DOES NOT ALLOW IT | | | | | | 0 | | 1 |  |
|  |  |  |  | E. FACILITY POLICY DOES NOT ALLOW IT | | | | | | 0 | | 1 |  |
|  |  |  |  | F. WANT TO KEEP THE ROOM CLEAN | | | | | | 0 | | 1 |  |
|  |  |  |  | G. IT IS DISTRACTING TO THE WOMAN IN LABOR | | | | | | 0 | | 1 |  |
|  |  |  |  | H. COMPANION WILL HARM OR BE RUDE TO PROVIDER | | | | | | 0 | | 1 |  |
|  |  |  |  | I. OTHER (SPECIFY) ________________ | | | | | | 0 | | 1 |  |
|  |  |  |  | J. DON’T KNOW / REMEMBER | | | | | | 0 | | 1 |  |
| ***READ:*** Now I am going to ask you questions about birth companionship at the moment of delivery. | | | | | | | | | | | | | |
| 3.8 | Do you allow women to bring a companion / support person into the delivery room? | | | NO…………………………………………………………………….….…..0  YES…………………………………………………………………………….1 DON’T KNOW / REMEMBER………………….……………..……8 | | | | | | | | | 3.11  3.11 |
| 3.9 | Do you allow a female companion/support person, a male companion/support person, or both? | | | FEMALE ONLY…………………………………………………………….1  MALE ONLY…………………………………………………………..……2  BOTH MALE AND FEMALE………………………………………….3  DON’T KNOW / REMEMBER……………….……………….…..…8 | | | | | | | | |  |
| 3.9A | At the time of delivery, do you allow women to have: | | |  | | | | **NO** | | | **YES** | **DK** |  |
|  | A. A companion or support person who the woman knows and brought from the community, who received a birth companionship orientation and ID badge | | | A. BROUGHT FORM HOME WITH ORIENTATION AND BADGE | | | | 0 | | | 1 | 8 |  |
|  | **ONLY ASK THIS QUESTION IF THE FACILITY HAS THE BIRTH COMPANION PROJECT**   B. A companion or support person who the woman knows and brought from the community, but who did not receive a birth companionship orientation or ID badge | | | B. BROUGHT FORM HOME WITHOUT ORIENTATION AND BADGE | | | | 0 | | | 1 | 8 |  |
|  | C. An on-call companion, based at your facility, who wears a grey uniform | | | C. ON-CALL WITH GRAY UNIFORM | | | | 0 | | | 1 | 8 |  |
|  | D. Other | | | D. OTHER SPECIFY: _________________________ | | | | 0 | | | 1 | 8 |  |
|  |  | | |  | | | | | |  | |  |  |
| 3.9B | Why do you allow a woman to bring a companion / support person into the delivery room?  ***CIRCLE ALL SPONTANEOUS ANSWERS AND PROBE:* Anything else?** | | |  | | | | | | **NO** | | **YES** |  |
|  |  |  |  | A. HELPS CLIENT FEEL MORE COMFORTABLE | | | | | | 0 | | 1 |  |
|  |  |  |  | B. ALLOWS PROVIDER TO BE WITH OTHER CLIENTS | | | | | | 0 | | 1 |  |
|  |  |  |  | C. HELPS PROIVDER WITH WORKLOAD | | | | | |  | |  |  |
|  |  |  |  | D. TELLS PROVIDERIF THERE IS A CHANGE OR A PROBLEM | | | | | | 0 | | 1 |  |
|  |  |  |  | E. GOVERNMENT POLICY ALLOWS IT | | | | | | 0 | | 1 |  |
|  |  |  |  | F. FACILITY POLICY ALLOWS IT | | | | | | 0 | | 1 |  |
|  |  |  |  | G. GETS THE CLIENT WHAT SHE NEEDS (EG, FOOD, DRINK) | | | | | | 0 | | 1 |  |
|  |  |  |  | H. GIVES CLIENT EMOTIONAL SUPPORT | | | | | | 0 | | 1 |  |
|  |  |  |  | I. GIVES CLIENT ADVICE | | | | | | 0 | | 1 |  |
|  |  |  |  | J. OTHER (SPECIFY) ________________ | | | | | | 0 | | 1 |  |
|  |  |  |  | K. DON’T KNOW / REMEMBER…………… | | | | | | 0 | | 1 |  |
| 3.9C | A. Would you say that allowing companions at the time of delivery has been satisfying or dissatisfying for you as a provider? | | | DISSATISFYING………………………………………………….………….1  SATISFYING…………………………………………………………………..2  NEITHER DISSATISFYING NOR SATISFYING…………………...3  DON’T KNOW………………………….………………………………...…8 | | | | | | | | | 3.9D  3.9D |
|  | B. Would you say it has been very *[…]* or a little *[…]*?   ***USE ANSWER FROM 3.9C_A IN PLACE OF […] (DISSATISFIED OR SATISFIED)*** | | | VERY DISSATISFIED………….…….…………………...……………….1  A LITTLE DISSATISFIED……………………..………..….…………....2  A LITTLE SATISFIED……………………………………………….…..…4  VERY SATISFIED………………………………………….………….….…5  DON’T KNOW………………………….…………………………….….…8 | | | | | | | | |  |
| 3.9D | A. Would you say that allowing companions at the time of delivery has been unhelpful to you as a provider or helpful to you? | | | UNHELPFUL………………………………………………….…………..….1  HELPFUL……………………………………………………………….……..2  NEITHER UNHELPFUL NOR HELPFUL………………….………...3  DON’T KNOW………………………….……………………………………8 | | | | | | | | | 3.9E  3.9E |
|  | B. Would you say it has been very *[…]* or a little *[…]*?   ***USE ANSWER FROM 3.9D_A IN PLACE OF […] (UNHELPFUL OR HELPFUL)*** | | | VERY UNHELPFUL………….………….…………………...…………….1  A LITTLE UNHELPFUL……………………..…..………..….…………..2  A LITTLE HELPFUL………………………………..………………….……4  VERY HELPFUL…………………………………………..….………………5  DON’T KNOW……………………………………………..………………..8 | | | | | | | | |  |
| 3.9E | Would you say that allowing companions at the time of delivery has made it harder for you to give good quality maternity care, has not changed your ability to give good quality maternity care, or has improved your ability to give good quality maternity care? | | | HAS MADE IT HARDER TO GIVE GOOD QUALITY CARE….1  HAS NOT CHANGED ABILITY TO GIVE GOOD QUALITY  CARE……………………………………………………………………………..2  HAS IMPROVED ABILITY TO GIVE GOOD QUALITY CARE..3  DON’T KNOW………………………………………………………………..8 | | | | | | | | |  |
| 3.9F | Would you say that allowing companions at the time of delivery has not met your expectations, met your expectations, or exceeded your expectations? | | | NOT MET EXPECTATIONS…………………………………….………..1  MET EXPECTATIONS……………………………………………………...2  EXCEEDED EXPECTATIONS……………………………………………..3  DON’T KNOW………………………….……..…………………………..…8 | | | | | | | | | 3.11A |
| 3.11 | Why do you not allow a woman to bring a companion/support person into the delivery room?  ***CIRCLE ALL SPONTANEOUS ANSWERS AND PROBE:* Anything else?** | | |  | | | | | | **NO** | | **YES** |  |
|  |  |  |  | A. ROOM IS TOO SMALL | | | | | | 0 | | 1 |  |
|  |  |  |  | B. PROBLEMS WITH PRIVACY | | | | | | 0 | | 1 |  |
|  |  |  |  | C. DON’T WANT COMPANION TO BRING HERBS | | | | | | 0 | | 1 |  |
|  |  |  |  | D. GOVERNMENT POLICY DOES NOT ALLOW IT | | | | | | 0 | | 1 |  |
|  |  |  |  | E. FACILITY POLICY DOES NOT ALLOW IT | | | | | | 0 | | 1 |  |
|  |  |  |  | F. WANT TO KEEP THE ROOM CLEAN | | | | | | 0 | | 1 |  |
|  |  |  |  | G. IT IS DISTRACTING TO THE WOMAN IN LABOR | | | | | | 0 | | 1 |  |
|  |  |  |  | H. COMPANION WILL HARM OR BE RUDE TO PROVIDER | | | | | | 0 | | 1 |  |
|  |  |  |  | I. OTHER (SPECIFY) ________________ | | | | | | 0 | | 1 |  |
|  |  |  |  | J. DON’T KNOW / REMEMBER | | | | | | 0 | | 1 |  |
| ***READ:*** Now I am going to ask you about postpartum companionship in the time after the baby is born. | | | | | | | | | | | | | |
| 3.11A | Do you allow women to have a companion / support person with them in the postpartum area after the baby is born? | | | NO………………………………………………………………….……..…..0  YES………………………………………………………………….………….1 DON’T KNOW / REMEMBER……………….…………….…..……8 | | | | | | | | | 3.11I  3.11I |
| 3.11B | Do you allow a female companion / support person, a male companion / support person, or both with them after the baby is born? | | | FEMALE ONLY…………………………………………………….……….1  MALE ONLY………………………………………………………..….……2  BOTH MALE AND FEMALE……………………………………….….3  DON’T KNOW / REMEMBER……………….……………….………8 | | | | | | | | |  |
| 3.11C | After the baby is born, do you allow women to have: | | |  | | | | **NO** | | | **YES** | **DK** |  |
|  | A. A companion or support person who the woman knows and brought from the community, who received a birth companionship orientation and ID badge | | | A. BROUGHT FORM HOME WITH ORIENTATION AND BADGE | | | | 0 | | | 1 | 8 |  |
|  | **ONLY ASK THIS QUESTION IF THE FACILITY HAS THE BIRTH COMPANION PROJECT**   B. A companion or support person who the woman knows and brought from the community, but who did not receive a birth companionship orientation or ID badge | | | B. BROUGHT FORM HOME WITHOUT ORIENTATION AND BADGE | | | | 0 | | | 1 | 8 |  |
|  | C. An on-call companion, based at your facility, who wears a grey uniform | | | C. ON-CALL WITH GRAY UNIFORM | | | | 0 | | | 1 | 8 |  |
|  | D. Other | | | D. OTHER SPECIFY: _________________________ | | | | 0 | | | 1 | 8 |  |
| 3.11D | Why do you allow a woman to have a companion / support person in the postpartum area after the baby is born?  ***CIRCLE ALL SPONTANEOUS ANSWERS AND PROBE:* Anything else?** | | |  | | | | | | **NO** | | **YES** |  |
|  |  |  |  | A. HELPS CLIENT FEEL MORE COMFORTABLE | | | | | | 0 | | 1 |  |
|  |  |  |  | B. ALLOWS PROVIDER TO BE WITH OTHER CLIENTS | | | | | | 0 | | 1 |  |
|  |  |  |  | C. HELPS PROVIDER WITH WORKLOAD | | | | | | 0 | | 1 |  |
|  |  |  |  | D. TELLS PROVIDER IF THERE IS A CHANGE OR A PROBLEM WITH CLIENT | | | | | | 0 | | 1 |  |
|  |  |  |  | E. TELLS PROVIDER IF THERE IS A CHANGE OR A PROBLEM WITH BABY | | | | | |  | |  |  |
|  |  |  |  | F. GOVERNMENT POLICY ALLOWS IT | | | | | | 0 | | 1 |  |
|  |  |  |  | G. FACILITY POLICY ALLOWS IT | | | | | | 0 | | 1 |  |
|  |  |  |  | H. GETS THE CLIENT WHAT SHE NEEDS (EG, FOOD, DRINK) | | | | | | 0 | | 1 |  |
|  |  |  |  | I. GIVES CLIENT EMOTIONAL SUPPORT | | | | | | 0 | | 1 |  |
|  |  |  |  | J. GIVES CLIENT ADVICE | | | | | | 0 | | 1 |  |
|  |  |  |  | K. HELPS CARE FOR BABY | | | | | | 0 | | 1 |  |
|  |  |  |  | L. HELPS WITH BREASTFEEDING | | | | | | 0 | | 1 |  |
|  |  |  |  | M. OTHER (SPECIFY) ________________ | | | | | | 0 | | 1 |  |
|  |  |  |  | N. DON’T KNOW / REMEMBER | | | | | | 0 | | 1 |  |
|  |  | | |  | | | | | | | | |  |
| 3.11E | A. Would you say that allowing companions into the postpartum area has been satisfying or dissatisfying for you as a provider? | | | DISSATISFYING………………………………………………….………….1  SATISFYING…………………………………………………………………..2  NEITHER DISSATISFYING NOR SATISFYING…………………...3  DON’T KNOW………………………….……………………………………8 | | | | | | | | | 3.11F  3.11F |
|  | B. Would you say it has been very *[…]* or a little *[…]*?   ***USE ANSWER FROM 3.11E_A IN PLACE OF […] (DISSATISFIED OR SATISFIED)*** | | | VERY DISSATISFIED………….…….……………………...…………….1  A LITTLE DISSATISFIED……………………..………..…….…………..2  A LITTLE SATISFIED………………………………………………….……4  VERY SATISFIED………………………………………….…………………5  DON’T KNOW………………………….……………………………………8 | | | | | | | | |  |
| 3.11F | A. Would you say that allowing companions into the postpartum area has been unhelpful to you as a provider or helpful to you? | | | UNHELPFUL………………………………………………….….…….…….1  HELPFUL………………………………………………………….…….……..2  NEITHER UNHELPFUL NOR HELPFUL…………………….……...3  DON’T KNOW………………………….………………………….……..…8 | | | | | | | | | 3.11G  3.11G |
|  | B. Would you say it has been very *[…]* or a little *[…]*?   ***USE ANSWER FROM 3.11F_A IN PLACE OF […] (UNHELPFUL OR HELPFUL)*** | | | VERY UNHELPFUL………….…….…………………...………..……….1  A LITTLE UNHELPFUL……………………..………..….…….………..2  A LITTLE HELPFUL…………………………………………….…….……4  VERY HELPFUL………………………………………….…….……………5  DON’T KNOW……………………………………………….……………..8 | | | | | | | | |  |
| 3.11G | Would you say that allowing companions into the postpartum area after the baby is born has made it harder for you to give good quality maternity care, has not changed your ability to give good quality maternity care, or has improved your ability to give good quality maternity care? | | | HAS MADE IT HARDER TO GIVE GOOD QUALITY CARE…1  HAS NOT CHANGED ABILITY TO GIVE GOOD QUALITY  CARE…………………………………………………………………………….2  HAS IMPROVED ABILITY TO GIVE GOOD QUALITY CARE.3  DON’T KNOW……………………………………………………………….8 | | | | | | | | |  |
| 3.11H | Would you say that allowing companions into the postpartum area after the baby is born has not met your expectations, met your expectations, or exceeded your expectations? | | | NOT MET EXPECTATIONS…………………………………….……….1  MET EXPECTATIONS……………………………………………………..2  EXCEEDED EXPECTATIONS…………………………………………....3  DON’T KNOW………………………….……..………………………….…8 | | | | | | | | | 3.12 |
| 3.11I | Why do you not allow a woman to have a companion/support person in the postpartum area after the baby is born?  ***CIRCLE ALL SPONTANEOUS ANSWERS AND PROBE:* Anything else?** | | |  | | | | | | **NO** | | **YES** |  |
|  |  |  |  | A. ROOM IS TOO SMALL | | | | | | 0 | | 1 |  |
|  |  |  |  | B. PROBLEMS WITH PRIVACY | | | | | | 0 | | 1 |  |
|  |  |  |  | C. DON’T WANT COMPANION TO BRING HERBS | | | | | | 0 | | 1 |  |
|  |  |  |  | D. GOVERNMENT POLICY DOES NOT ALLOW IT | | | | | | 0 | | 1 |  |
|  |  |  |  | E. FACILITY POLICY DOES NOT ALLOW IT | | | | | | 0 | | 1 |  |
|  |  |  |  | F. WANT TO KEEP THE ROOM CLEAN | | | | | | 0 | | 1 |  |
|  |  |  |  | G. IT IS DISTRACTING TO THE WOMAN | | | | | | 0 | | 1 |  |
|  |  |  |  | H. COMPANION WILL HARM OR BE RUDE TO PROVIDER | | | | | | 0 | | 1 |  |
|  |  |  |  | I. OTHER (SPECIFY) ________________ | | | | | | 0 | | 1 |  |
|  |  |  |  | J. DON’T KNOW / REMEMBER | | | | | | 0 | | 1 |  |
| 3.12 | After a normal delivery, do you ask a client to return to the health facility? | | | NO………………………………………………………………………..…..0  YES…………………………………………………………………………….1 DON’T KNOW / REMEMBER………………….………………..…8 | | | | | | | | | 3.15  3.15 |
| 3.13 | When do you usually ask her to return?  ***CIRCLE ALL SPONTANEOUS ANSWERS AND PROBE:* Any other time?** | | | DURING THE FIRST 48 HOURS (2 DAYS)………………………1  DURING THE FIRST WEEK……………………………………………2  DURING THE FIRST 6 WEEKS…………………………………..….3  IF SHE IS ILL / IN CASE OF PROBLEM…………….…………….4  IF BABY IS ILL / IN CASE OF BABY PROBLEM……………….5  OTHER (SPECIFY) _________________________….……7  DON’T KNOW / REMEMBER………………….…………….….….8 | | | | | | | | |  |
| 3.15 | When was the last time that you encountered a woman with…  ***READ LIST*** | **NEVER** | **IN THE PAST WEEK** | | **IN THE PAST MONTH** | **IN THE PAST 3 MONTHS** | **IN THE PAST 6 MONTHS** | | **MORE THAN 6 MONTHS AGO** | | | **DK** |  |
|  | A. Postpartum hemorrhage? | 0 | 1 | | 2 | 3 | 4 | | 5 | | | 8 |  |
|  | B. Obstructed labor? | 0 | 1 | | 2 | 3 | 4 | | 5 | | | 8 |  |
|  | C. Puerperal sepsis? | 0 | 1 | | 2 | 3 | 4 | | 5 | | | 8 |  |
|  | D. Eclampsia? | 0 | 1 | | 2 | 3 | 4 | | 5 | | | 8 |  |
| 3.16 | For a woman in labor, what observations do you make as you monitor her progress?  ***CIRCLE ALL SPONTANEOUS ANSWERS AND ASK:*Anything else?** | | |  | | | | | **NO** | | | **YES** |  |
|  |  |  |  | A. FETAL HEARTBEAT | | | | | 0 | | | 1 |  |
|  |  |  |  | B. COLOR OF AMNIOTIC FLUID | | | | | 0 | | | 1 |  |
|  |  |  |  | C. DEGREE OF MOLDING | | | | | 0 | | | 1 |  |
|  |  |  |  | D. DILATATION OF THE CERVIX | | | | | 0 | | | 1 |  |
|  |  |  |  | E. DESCENT OF THE HEAD | | | | | 0 | | | 1 |  |
|  |  |  |  | F. STRENGTH OF UTERINE CONTRACTIONS | | | | | 0 | | | 1 |  |
|  |  |  |  | G. FREQUENCY OF UTERINE CONTRACTIONS | | | | | 0 | | | 1 |  |
|  |  |  |  | H. MATERNAL BLOOD PRESSURE | | | | | 0 | | | 1 |  |
|  |  |  |  | I. MATERNAL TEMPERATURE | | | | | 0 | | | 1 |  |
|  |  |  |  | J. MATERNAL PULSE | | | | | 0 | | | 1 |  |
|  |  |  |  | K. FETAL BODY POSITION (BREECH, VERTEX) | | | | | 0 | | | 1 |  |
|  |  |  |  | L. FETAL HEAD POSITION | | | | | 0 | | | 1 |  |
|  |  |  |  | M. OTHER (SPECIFY) ______________ | | | | | 0 | | | 1 |  |
|  |  |  |  | N. DON’T KNOW / REMEMBER | | | | | 0 | | | 1 |  |
| 3.17 | Where do you register these observations?  ***CIRCLE ALL SPONTANEOUS ANSWERS AND ASK:*Anything else?** | | |  | | | | | **NO** | | | **YES** |  |
|  |  |  |  | A. ON A PARTOGRAM | | | | | 0 | | | 1 |  |
|  |  |  |  | B. IN THE PATIENT’S CLINICAL RECORD | | | | | 0 | | | 1 |  |
|  |  |  |  | C. IN THE PRENATAL CARD | | | | | 0 | | | 1 |  |
|  |  |  |  | D. ON A PIECE OF PAPER | | | | | 0 | | | 1 |  |
|  |  |  |  | E. IN THE MATERNITY REGISTER | | | | | 0 | | | 1 |  |
|  |  |  |  | F. OTHER (SPECIFY) _______________ | | | | | 0 | | | 1 |  |
|  |  |  |  | G. DON’T KNOW / REMEMBER | | | | | 0 | | | 1 |  |
| **SECTION 5. WORK ENVIRONMENT AND SUPERVISION** | | | | | | | | | | | | | |
| 5.1 | In an average week, how many hours do you work in this facility?  ***IF WEEKS ARE INCONSISTENT, ASK RESPONDENT TO AVERAGE NUMBER OF HOURS PER MONTH AND DIVIDE BY 4.*** | | | AVERAGE HOURS PER WEEK WORKING …….. [ __ ] [ __ ]  DON’T KNOW / REMEMBER…………………….……….………88 | | | | | | | | |  |
| 5.2 | A. How would you rate your overall satisfaction with your work situation? Would you say you are dissatisfied or satisfied? | | | DISSATISFIED………………………………………………….………….1  SATISFIED…………………………………………………………………..2  NEITHER DISSATISFIED NOR SATISFIED…………………..….3  DON’T KNOW………………………….…………………………………8 | | | | | | | | | 5.5  5.5 |
|  | B. Would you say you are very *[…]* or a little *[…]*?   ***USE ANSWER FROM 5.2A IN PLACE OF […] (DISSATISFIED OR SATISFIED)*** | | | VERY DISSATISFIED………….…….…………………...…………….1  A LITTLE DISSATISFIED……………………..………..….…………..2  A LITTLE SATISFIED……………………………………………….……4  VERY SATISFIED………………………………………….………………5 | | | | | | | | |  |
|  |  | | |  | | | | | |  | |  |  |
| 5.5 | Among the various things related to your working situation that you would like to see improved, can you tell me the three that you think would most improve your ability to provide good quality of care services? Please rank them in order of importance, with 1 being the most important.  ***ENTER THE LETTER CORRESPONDING WITH THE 1^ST^ MENTIONED IN THE 1ST SPACE BELOW, AND REPEAT WITH THE 2^ND^ AND 3^RD^***  ***IF THE PROVIDER ONLY MENTIONS 1 OR 2 ITEMS THEN LEAVE THE REMAINING BOXES EMPTY. THERE MUST BE AT LEAST 1 ENTRY.***  *________ ________ ________*  *1^st^ 2^nd^ 3^rd^* | | |  | | | | | | **NO** | | **YES** |  |
|  |  |  |  | A. MORE SUPPORT FROM SUPERVISOR | | | | | | 0 | | 1 |  |
|  |  |  |  | B. MORE KNOWLEDGE / UPDATES TRAINING | | | | | | 0 | | 1 |  |
|  |  |  |  | C. MORE SUPPLIES / STOCK | | | | | | 0 | | 1 |  |
|  |  |  |  | D. BETTER QUALITY EQUIPMENT / SUPPLIES | | | | | | 0 | | 1 |  |
|  |  |  |  | E. LESS WORKLOAD (E.G., MORE STAFF) | | | | | | 0 | | 1 |  |
|  |  |  |  | F. BETTER WORKING HOURS / FLEXIBLE TIMES | | | | | | 0 | | 1 |  |
|  |  |  |  | G. MORE INCENTIVES (SALARY, PROMOTION, HOLIDAYS) | | | | | | 0 | | 1 |  |
|  |  |  |  | H. TRANSPORTATION FOR REFERRAL PATIENTS | | | | | | 0 | | 1 |  |
|  |  |  |  | I. PROVIDING ART | | | | | | 0 | | 1 |  |
|  |  |  |  | J. PROVIDING POST EXPOSURE PROPHYLAXIS (PEP) | | | | | | 0 | | 1 |  |
|  |  |  |  | K. INCREASED SECURITY | | | | | | 0 | | 1 |  |
|  |  |  |  | L. BETTER FACILITY INFRASTRUCTURE | | | | | | 0 | | 1 |  |
|  |  |  |  | M. MORE AUTONOMY / INDEPENDENCE | | | | | | 0 | | 1 |  |
|  |  |  |  | N. EMOTIONAL SUPPORT FOR STAFF (COUNSELING/SOCIAL ACTIVITIES) | | | | | | 0 | | 1 |  |
|  |  |  |  | O. PAID LEAVE TO ATTEND TRAINING | | | | | | 0 | | 1 |  |
|  |  |  |  | P. OTHER (SPECIFY) __________________ | | | | | | 0 | | 1 |  |
|  |  |  |  | Q. DON’T KNOW | | | | | | 0 | | 1 |  |
| 5.6 | Do you feel you are paid fairly for the job duties you perform? | | | NO………………………………………………………………….………….0  YES……………………………………………..……………………………..1  DON’T KNOW / REMEMBER………………………………….…..8 | | | | | | | | |  |

**SECTION 6. TRAINING**

*I am going to read a list of services. For each one I would like you to tell me if you’ve been trained (during pre-service or in-service training) to provide the service, and if you have provided the service in the last 3 months.****ASK QUESTIONS ROW BY ROW.***

|  | | **Service** | a. Did you receive pre-service training on how to...?  ***PLACE CHECK (****✓****)*** | | b. Have you received in-service training on how to…?  ***PLACE CHECK (****✓****)*** | | c. When was the most recent time you received this training?  ***ENTER YEAR OF TRAINING*** | | d. If received training, who provided this training?  ***ENTER NAME OF ORGANIZATION*** | | | | e. Have you provided this service in the past  3 months?  ***PLACE CHECK (****✓****)*** | | |
| --- | --- | --- | --- | --- | --- | --- | --- | --- | --- | --- | --- | --- | --- | --- | --- |
|  | |  | **Yes** | **No** | **Yes** | **No** | **YEAR** | | **ORGANIZATION** | | | | **Yes** | | **No** |
| **Delivery Services** | | | | | | | | | | | | | | | |
| 6.2 | | Provide routine labor and delivery care |  |  |  |  |  | |  | | | |  | |  |
| 6.2.0 | | Practice delivery using PRONTO Pack simulation |  |  |  |  |  | |  | | | |  | |  |
| 6.2.1 | | Provide respectful maternity care |  |  |  |  |  | |  | | | |  | |  |
| 6.2.2 | | Allow women to have a companion during labor |  |  |  |  |  | |  | | | |  | |  |
| 6.2.3 | | Allow women to have a companion at delivery |  |  |  |  |  | |  | | | |  | |  |
| **General Training** | | | | | | | | | | | | | | | |
| 6.45 | Do you feel you receive adequate training to perform your job duties well? | | | | | | | **NO** | | **YES** | **NA** | **DK** | |  | |
|  |  |  |  |  |  |  |  | 0 | | 1 | 6 | 8 | |  |  |
| 6.46 | Has the in-service training you have received helped you to improve how you perform your job duties? | | | | | | | **NO** | | **YES** | **NA** | **DK** | |  | |
|  |  |  |  |  |  |  |  | 0 | | 1 | 6 | 8 | |  |  |
| 6.47 | Have you ever used the following clinical support and mentoring opportunities…  ***READ LIST*** | | | | | | | **NO** | | **YES** | **NA** | **DK** | |  | |
|  | A. A weekly teleconference to have a discussion with an OB/GYN consultant? | | | | | | | 0 | | 1 | 6 | 8 | |  | |
|  | B. An emergency call system to get advice on how to manage an emergency case? | | | | | | | 0 | | 1 | 6 | 8 | |  | |
|  | C. An E-learning platform, such as a computer, smartphone, or tablet, at your facility to access continuing education courses? | | | | | | | 0 | | 1 | 6 | 8 | |  | |

Thank you for taking the time to speak to us today.
